# Third-generation genome sequencing implicates medium-sized structural variants in chronic schizophrenia

**DOI:** 10.3389/fnins.2022.1058359

**Published:** 2023-01-11

**Authors:** Chi Chiu Lee, Rui Ye, Justin D. Tubbs, Larry Baum, Yuanxin Zhong, Shuk Yan Joey Leung, Sheung Chun Chan, Kit Ying Kitty Wu, Po Kwan Jamie Cheng, Lai Ping Chow, Patrick W. L. Leung, Pak Chung Sham

**Affiliations:** ^1^Department of Psychiatry, Kwai Chung Hospital, Hong Kong, Hong Kong SAR, China; ^2^Department of Psychiatry, The University of Hong Kong, Hong Kong, Hong Kong SAR, China; ^3^State Key Laboratory of Brain and Cognitive Sciences, The University of Hong Kong, Hong Kong, Hong Kong SAR, China; ^4^Department of Psychiatry, Tai Po Hospital, Hong Kong, Hong Kong SAR, China; ^5^Kowloon West Cluster, Hospital Authority, Hong Kong, Hong Kong SAR, China; ^6^Department of Clinical Psychology, Yan Chai Hospital, Hong Kong, Hong Kong SAR, China; ^7^Department of Psychology, The Chinese University of Hong Kong, Hong Kong, Hong Kong SAR, China; ^8^Centre for PanorOmic Sciences, The University of Hong Kong, Hong Kong, Hong Kong SAR, China

**Keywords:** schizophrenia, chronic and negative symptoms, third generation sequencing, multiplex families, intronic, structural variants, biological pathways, striatum

## Abstract

**Background:**

Schizophrenia (SCZ) is a heterogeneous psychiatric disorder, with significant contribution from genetic factors particularly for chronic cases with negative symptoms and cognitive deficits. To date, Genome Wide Association Studies (GWAS) and exome sequencing have associated SCZ with a number of single nucleotide polymorphisms (SNPs) and copy number variants (CNVs), but there is still missing heritability. Medium-sized structural variants (SVs) are difficult to detect using SNP arrays or second generation sequencing, and may account for part of the missing heritability of SCZ.

**Aims and objectives:**

To identify SVs associated with severe chronic SCZ across the whole genome.

**Study design:**

10 multiplex families with probands suffering from chronic SCZ with negative symptoms and cognitive deficits were recruited, with all their affected members demonstrating uni-lineal inheritance. Control subjects comprised one affected member from the affected lineage, and unaffected members from each paternal and maternal lineage.

**Methods:**

Third generation sequencing was applied to peripheral blood samples from 10 probands and 5 unaffected controls. Bioinformatic tools were used to identify SVs from the long sequencing reads, with confirmation of findings in probands by short-read Illumina sequencing, Sanger sequencing and visual manual validation with Integrated Genome Browser.

**Results:**

In the 10 probands, we identified and validated 88 SVs (mostly in introns and medium-sized), within 79 genes, which were absent in the 5 unaffected control subjects. These 79 genes were enriched in 20 biological pathways which were related to brain development, neuronal migration, neurogenesis, neuronal/synaptic function, learning/memory, and hearing. These identified SVs also showed evidence for enrichment of genes that are highly expressed in the adolescent striatum.

**Conclusion:**

A substantial part of the missing heritability in SCZ may be explained by medium-sized SVs detectable only by third generation sequencing. We have identified a number of such SVs potentially conferring risk for SCZ, which implicate multiple brain-related genes and pathways. In addition to previously-identified pathways involved in SCZ such as neurodevelopment and neuronal/synaptic functioning, we also found novel evidence for enrichment in hearing-related pathways and genes expressed in the adolescent striatum.

## Introduction

Schizophrenia is a complex psychiatric disorder, with a median lifetime morbid risk of 7.2 in 1,000 persons (Saha et al., 2005), which incurs enormous economic, personal and social costs (Whiteford et al., 2013). Genetic factors contribute substantially to the variation in liability to the disorder, with twin and family studies estimating a heritability of 0.6–0.8 (Sullivan et al., 2003; Lichtenstein et al., 2009). The risk of being affected by schizophrenia is at least 18 fold greater in first-degree relatives than in the general population (Kendler et al., 1985).

Schizophrenia has multiple manifestations, which have been classified into independent yet co-existing symptom dimensions (MacDonald and Schulz, 2009). Nevertheless, there have been attempts to classify schizophrenia into distinct syndromes, according to positive-negative symptoms and chronicity of illness – negative symptoms are usually chronic while positive symptoms are characteristic of acute schizophrenic episodes (Crow, 1980; Liddle and Barnes, 1990). Symptoms may reflect underlying pathophysiological mechanisms, as they appear to be partially familial and heritable (McGrath et al., 2009).

Negative symptoms are so named because there is an absence or deficit of certain functions and occur in 28–36% of schizophrenia patients (Blanchard et al., 2005). Negative symptoms can be primary, when they are etiologically related to the core pathophysiology of schizophrenia, or secondary, when they are derivatives of other comorbid disease processes such as depression, medication side effects, or impoverished environments. Chronic schizophrenia with negative symptoms is more difficult to treat than acute psychosis (Usall et al., 2014) and leads to high levels of burden reported by caregivers.

Similar to negative symptoms, cognitive deficits in domains such as working memory, attention, learning, problem solving, processing speed, and social cognition (August et al., 2012) are also related to poor outcomes and little influenced by antipsychotic treatment (Carpenter et al., 1999; Kahn and Keefe, 2013). Studies revealed that 75% of patients with schizophrenia had impairment in at least two cognitive domains (O’Carroll, 2000). Cognitive impairment is often present prior to the onset of psychotic symptoms. Cognitive deficits were noted in unaffected first-degree relatives of schizophrenic patients, suggesting a genetic relationship between cognitive deficits and schizophrenia (Ripke et al., 2014). This genetic overlap is further supported by converging evidence from twin (Toulopoulou et al., 2007) and genome-wide (Toulopoulou et al., 2019; Schizophrenia Working Group of the PGC et al., 2020) approaches. The presence of both negative symptoms and cognitive deficiencies represents a severe subgroup of chronic schizophrenia patients, forming a more homogeneous, stable and restrictive phenotype that affords greater power for discovering genetic variants that contribute large effects on schizophrenia risk.

Recent efforts to identify genetic risk factors for schizophrenia have relied on two methodological advances in human genetics, Genome-Wide Association Studies (GWAS) and DNA sequencing. GWAS aims to identify common variants (CVs) associated with a disease by genotyping representative single nucleotide polymorphisms (SNPs) spanning the whole human genome. In a landmark 2014 GWAS analysis, the Psychiatric Genomics Consortium (PGC) identified 128 independent associations in 108 loci (Ripke et al., 2014). With an expanded sample size, the number of associated loci has more than doubled to 270 (Schizophrenia Working Group of the PGC et al., 2020). These CV associations were shown to be enriched among genes expressed in brain tissues (Zhao and Nyholt, 2016), and have been instrumental for elucidating biological pathways, including neuronal signaling, development, and differentiation (Schizophrenia Working Group of the PGC et al., 2020).

The success of GWAS is based on the“common disease, common variant” (CDCV) hypothesis, which proposes that genetic influences on common diseases are largely attributable to allelic variants that are common (frequency above 1%) in the population. However, CVs detected for schizophrenia have been estimated to account for only 24% of the total variance in liability. This shortfall from the overall heritability of 70–80% estimated by twin studies (missing heritability) implies a contribution from other types of genetic variants (Maher, 2008; Purcell et al., 2014).

The study of Rare Variants (RVs) which have allele frequencies of less than 0.5% in schizophrenia is important for a number of reasons. Firstly, schizophrenic patients have substantially reduced reproductive fecundity, so genetic variants with large effect size are expected to be kept at a very low frequency in the population as a result of continuing purifying selection. Secondly, the small effect sizes of CVs identified by GWAS make it difficult to interpret their roles in the pathophysiological mechanisms of schizophrenia. In contrast, RVs, which are either inherited from parents or arise *de novo*, have larger effect sizes and more readily interpretable functional consequences on their respective molecular pathways. The study of RVs, including both single nucleotide variants (SNVs) and Structural Variants (SVs), is thus likely to provide greater biological insight on schizophrenia.

With the advent of next generation sequencing (NGS) technology, the cost of sequencing has dropped precipitously, allowing whole exomes or even whole genomes to be examined for the characterization of RVs. Early sequencing studies identified *de novo* CNVs and rare SVs (Rujescu et al., 2009; Vacic et al., 2011), while later studies have reported an excess of rare coding mutations (Genovese et al., 2016) and an enrichment of non-synonymous ultra-rare variants (URVs) (Gratten, 2016). Taken together, the findings from sequencing studies and GWAS suggest that schizophrenia is the result of the cumulative effects of both rare and common variants that disrupt the function of a large number of genes. Thus, polygenicity, well established for CVs in schizophrenia and other neuropsychiatric diseases, also firmly applies to RV and URV coding mutations, and provides a plausible explanation for the high global incidence and persistence of schizophrenia (Walsh et al., 2008).

Whole exome sequencing (WES) with NGS technology is best for detecting SNVs within protein-coding sequences, including stop-gains and frameshifts, which are likely to lead to the complete loss of function of the protein product. Many specific *de novo* and inherited RVs in coding regions have been associated with schizophrenia (Degenhardt et al., 2016; Giacopuzzi et al., 2017; Xue et al., 2019; Rees et al., 2020). Recently, a large collaborative study has identified rare coding variants in 10 genes that confer substantial risk for schizophrenia (Singh et al., 2022). However, despite these discoveries, the diagnostic yield from exome sequencing in schizophrenia is low (Balakrishna and Curtis, 2020) and disease-related RV can only be detected in a small proportion of schizophrenic patients. In addition, mutations in non-coding regions can still have significant impacts on gene expression, variants in non-coding regulatory genome elements may be involved in schizophrenia and account for part of the missing heritability.

Large CNVs, which can be detected by SNP array, have been consistently reported to be associated with schizophrenia. They have much larger effect sizes than common SNPs, with risk ratios ranging from 3 to 20 (Rees et al., 2014; Bergen et al., 2019). However, many SVs are not long enough for SNP arrays to detect, and whole genome sequencing (WGS) with NGS has been used to identify an increased burden of rare, exonic CNVs in the probands of 91 multiplex schizophrenia families (Khan et al., 2018). NGS typically has a read length of only 100–150 bases, sufficient for detecting SVs under 50 bps, but not for identifying most medium sized SVs, which are in the range of 50–2,000 bp. These medium-size SVs are more common in the human genome than large CNVs, but are nevertheless likely to have a larger effect on gene expression than single base pair changes affecting regulatory elements. Third-generation-sequencing (TGS) technology like PacBio Sequel can generate continuous sequences ranging from 10 kb to several megabases directly from native DNA, allowing accurate characterization of medium size SVs.

While the case-control design has proved to be highly effective for GWAS of common variants, family-based designs are necessary for delineating *de novo* variation, and may be advantageous for studying ultra-rare, high-penetrance variants. Simplex pedigrees (with only a single affected individual per family) are ideal for studying *de novo* mutations, which may have large effects on disease risk and reproductive fitness. For variants with modest penetrance and therefore weaker effect on fecundity, a multiple pedigree design may be more appropriate, as such families may be more likely than simplex families to segregate variants with moderately strong effect size. Amongst multiplex pedigrees, those that are unilineal, with all affected members coming from one side of the family (either paternal or maternal), are more likely to harbor high-penetrance dominant variants. The smaller sample size requirement of the pedigree design than of the case-control design makes it an ideal start for exploiting the greater power of TGS to detect medium-sized structural variants given its current high cost.

The present study is an initial examination of medium-sized SVs in schizophrenia using third generation sequencing. A multiplex family design was adopted, based on the hypothesis that medium size SVs have moderately large effect sizes, larger than those for SNPs but smaller than those for large CNVs, and assuming that multiplex families are enriched for such SVs. The affected probands were selected to have severe disorder characteristics with negative symptoms and cognitive impairment, in an attempt to further enrich the families for SVs. If SVs of moderately large effect size are indeed prevalent in multiplex families, we can expect to detect many such SVs even in a small number of families. The demonstration of a role for medium-sized SVs in schizophrenia would have a major impetus to revise the current thinking regarding the etiology, pathophysiology, prevention and treatment of the common yet devastating disorder of schizophrenia.

## Materials and methods

### Participants

The participants were 10 Chinese unilineal multiplex schizophrenia families recruited from a psychiatric hospital in Hong Kong. The probands were chronic schizophrenic patients with negative symptoms. A restrictive phenotype of chronic schizophrenia with negative symptoms in unilineal multiplex pedigrees was chosen in accordance with our hypothesis.

### Methodology

#### Phenotyping

All probands had ages between 18 and 70 years, onset of psychotic symptoms before age 30, IQ higher than 80, negative symptoms, and symptoms that persisted longer than 5 years not responding to treatment with at least two types of antipsychotic medications. Exclusion criteria included the presence of neurological diseases, history of substance-induced psychosis, or co-morbidity with bipolar disorder, depressive disorder, attention deficit/hyperactivity disorder or autism spectrum disorder.

One additional affected member in the family and one unaffected member from one or both sides of the family, chosen among the first or second degree relatives of the affected proband, were recruited. The unaffected family members were adults who had never reported any schizophrenic symptoms so far, and served as controls.

The Chinese version of the Structured Clinical Interview for DSM-IV Axis I Disorders (SCID-1) (So et al., 2003) was used to confirm the diagnosis of schizophrenia in cases and to screen for past or current undiagnosed psychiatric disorder in controls. A battery of neurocognitive tests was also performed for all recruited subjects. These included the intellectual assessment using Wechsler Adult Intelligence Scale 4th Edition (WAIS-IV; Wechsler, 2008); the Tower of London (Krikorian et al., 1994), Stroop Test (Stroop, 1935; Golden and Freshwater, 2002) and Digit Vigilance Test (DVT; Lewis, 1995) to measure executive functioning (EF) and attention; the Rey Complex Figure Test (RCFT; Bernstein and Waber, 1996) and Hong Kong List Learning Test – 2nd Edition (HKLLT; Chan, 2006) to assess learning and memory; the Verbal Fluency Test (Lezak et al., 2012); and the Colour Trail Making Test (D’Elia et al., 1996).

Affected individuals were subjected to an additional battery of tests to identify those who fit the restrictive phenotype definition of schizophrenia with primary negative symptoms. The Scale for the Assessment of Positive Symptoms (SAPS) (Andreasen, 1984) and Scale for the Assessment of Negative Symptoms (SANS) (Andreasen, 1989) were used to measure the symptom subgroups of schizophrenia. The Global Assessment of Functioning Scale (GAF) of Diagnostic and Statistical Manual IV (DSM-IV TR) was used to rate the social, occupational, and psychological functioning of patients. The Calgary Depression Scale for Schizophrenia (CDSS) (Addington et al., 1990) was used to separately assess depressive symptoms from positive, negative and extrapyramidal symptoms in patients.

The project proposal was endorsed by the IRB of the Hospital Authority of Hong Kong. The SCID-1, SAPS, SANS, GAF and CDSS were administered by trained psychiatrists, and the neurocognitive tests by clinical psychologists associated with the research team.

#### Workflow

**FIGURE 1:**
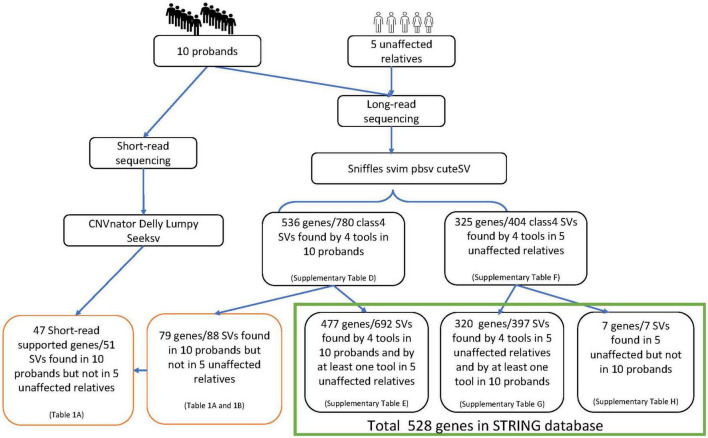
Workflow of the project.

Blood samples were collected after informed consent. DNA extraction, library preparation and sequencing were performed by the Centre for PanorOmic Sciences (CPOS) in the University of Hong Kong. Long-read PacBio sequencing (PacBio) was used to detect structural variants in the 10 probands. The SVs were subsequently validated with NGS (using Illumina NovaSeq6000) and Sanger sequencing. Five additional unaffected subjects from the affected lineages who were genetically closest to the probands were also selected for sequencing with PacBio, to help filter out SVs that may not play a role in schizophrenia. Multiple programs, namely sniffles, svim, pbsv and cuteSV, were used to maximize the specificity of SV detection and filtering from PacBio data. Similarly, CNVnator, delly, lumpy and seeksv were used for calling SVs from Illumina data (Figure 1).

#### PacBio WGS

Large-insert (>30 kb) PacBio libraries were constructed from DNA samples using the SMRTbell^®^ Express Template Preparation Kit 2.0 [PN 101-693-800 Version 01 (January 2019)]. After library QC, each library was sequenced on the PacBio Sequel system. For sequencing preparation, primer annealing and polymerase binding were performed based on the binding calculator as instructed by PacBio. At least 5 Sequel SMRT Cell 1M were run per sample to generate approximately 10X coverage for structural variation profiling.

##### Alignment and quality control of PacBio data

Raw reads were aligned to human genome reference hg19 using NGMLR (Sedlazeck et al., 2018) version 0.2.7. Then, the aligned reads were sorted according to their genome positions, and quality control statistics were generated, by samtools (Li et al., 2009b) version 1.9. All samples passed quality control filtering, as detailed in the Supplementary Figures A–F.

##### Validation and further analyses

Structural variants found to be potentially pathogenic (as described in the *Annotation and Pathway Enrichment* section below) were validated with short read whole genome sequencing using Illumina and Sanger sequencing. Those that are confirmed to be present in the patient and affected family members, but absent in unaffected relatives, are considered candidate high-penetrance rare structural variants for schizophrenia.

#### Short read Illumina whole genome sequencing (Illumina)

All libraries were prepared based on the protocols of KAPA Hyper Prep Kit (KR0961-V1.14). The PCR-free libraries were quantified by qPCR, denatured, and diluted to optimal concentration. Illumina NovaSeq 6000 (Illumina) was used for Pair-End 151 bp sequencing.

##### Alignment and quality control of Illumina data

The raw fastq files were screened by fastQC (Andrews, 2010) version 0.10.1 to check whether they meet the basic quality thresholds: per base sequence quality, per sequence quality scores, per base sequence content, per base GC content, per sequence GC content, per base N content, and kmer content. The Lafiltered reads were subsequently aligned to hg19 using BWA mem (Li and Durbin, 2009a) version 0.7.12-r1039, then sorted according to their genome positions using samtools (Li et al., 2009b) version v1.1. VerifyBamID (Jun et al., 2012) version 1.1.3 was then used to check for cross-contamination of samples. Picard (Broad Institute, 2016) version 1.127 MarkDuplicates was used to remove PCR duplicate reads.

#### SV calling on PacBio and Illumina data

**FIGURE 2:**
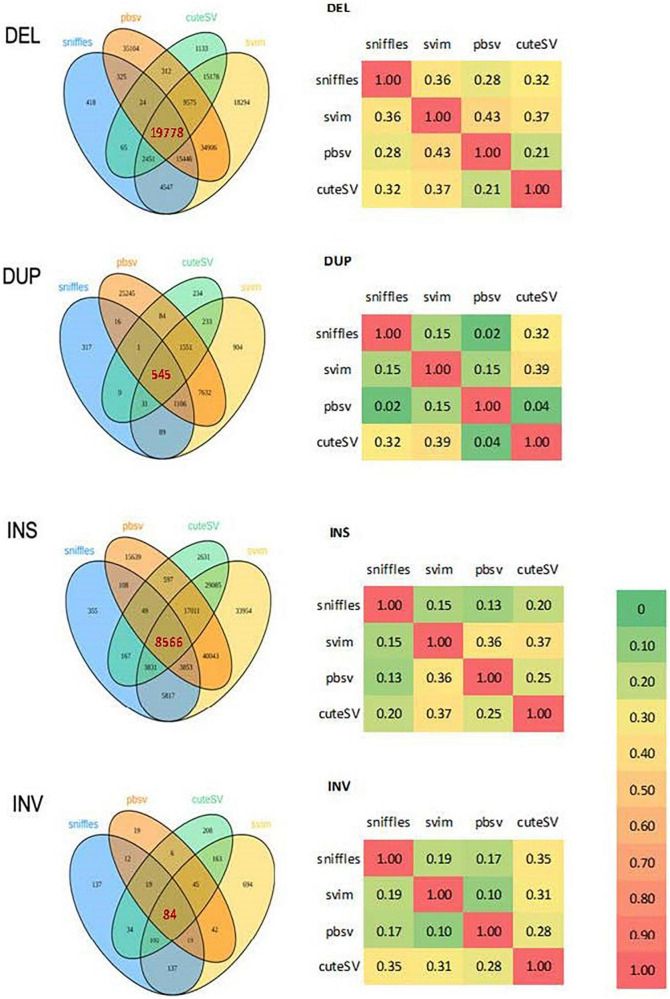
Venn diagrams and correlation matrices of structural variations detected by 4 programs from PacBio in 10 probands. DEL, deletion; DUP, duplication; INS, insertion; INV, inversion.

Four tools were used to detect SVs from PacBio data: Sniffles (Sedlazeck et al., 2018) v1.0.11, svim (Heller and Vingron, 2019) v1.4.1, pbsv (Wenger et al., 2021) v2.3.0 and cuteSV (Jiang et al., 2020) 1.0.8. Consensus SVs found by multiple tools with a reciprocal overlap rate ≥ 0.7 were selected for subsequent annotation and pathway enrichment analyses (Figure 2). Default parameters were used for all tools except for the minimum SV length, which was set to 30 bp.

Four tools were used for SV calling using Illumina data: CNVnator v0.3 (Abyzov et al., 2011), delly v0.7.7 (Rausch et al., 2012), lumpy v0.2.12 (Layer et al., 2014) and seeksv v1.2.2 (Liang et al., 2017). All tools used default parameters, except that a bin size of 50 bp was set for CNVnator.

#### Sanger sequencing

To confirm 8 deletions and 29 insertions which were not confirmed by SV calling on Illumina data, touchdown PCR and Sanger sequencing was performed in probands. Mono- or di-nucleotide repeats in most of the amplicons often led to messy sequences. Nested PCR was used for some mutations that initially could not be clearly sequenced.

#### Annotation and pathway enrichment

The detected SVs were annotated by AnnotSV (Geoffroy et al., 2018) v2.4 and classified as benign (class 1), likely benign (class 2), uncertain significance (class 3), likely pathogenic (class 4) or pathogenic (class 5), according to ACMG guidelines (Richards et al., 2015). To shorten the candidate gene list, only the most likely pathogenic SVs of class 4 or above were selected. No class 5 pathogenic SVs were identified in this study.

We used a web-based tool, STRING (Szklarczyk et al., 2019) v11.5, to perform pathway enrichment on each set of SV-containing genes. We applied the default hypergeometric test for pathway over-representation, using all Homo sapiens genes as the background set, and employed the Benjamini-Hochberg procedure for calculating the false-discovery rate (FDR) for each pathway. We retained only those Gene Ontology (GO) pathways with background gene counts between 30 and 1,000, an FDR < 0.05 and strength > 0.5.

#### Longitudinal brain region enrichment analysis

To test for enrichment of brain regions across developmental stages, we used the ABAEnrichment package (Grote et al., 2016) in R version 4.1 (R Core Team, 2020). Briefly, this package tests for enrichment among 16 brain regions across each of 5 developmental stages using RNAseq data available through the Brainspan project (Sunkin et al., 2013). For each region-stage combination, ABAEnrichment classifies each gene into high and low expression groups using a quantile-based cutoff. The package then tests for enrichment of a user-provided gene list in the highly expressed genes versus a background gene set using a hypergeometric test. Finally, ABAEnrichment also calculates a family-wise error rate (FWER) by permuting the target gene set and testing for enrichment across many iterations.

Thus, we used ABAEnrichment to perform competitive tests for enrichment of the set of genes containing case-specific SVs across brain development. Five equally-spaced quantile cutoff values were considered between 0.5 and 0.9. The background gene set included those which we found to have biased expression in brain tissue compared to other body tissues either pre or post-natally. Specifically, we calculated the log base 2 fold-change (FC) in brain versus all other tissues in two publicly available datasets, Descartes (Cao et al., 2020) and GTex (Carithers et al., 2015), and considered those with a log2 FC greater than 0.5 in either dataset to have biased brain expression. This brain-biased set of genes was then used in the ABAEnrichment analysis as the background gene set.

## Results

### Description of subjects

**FIGURE 3:**
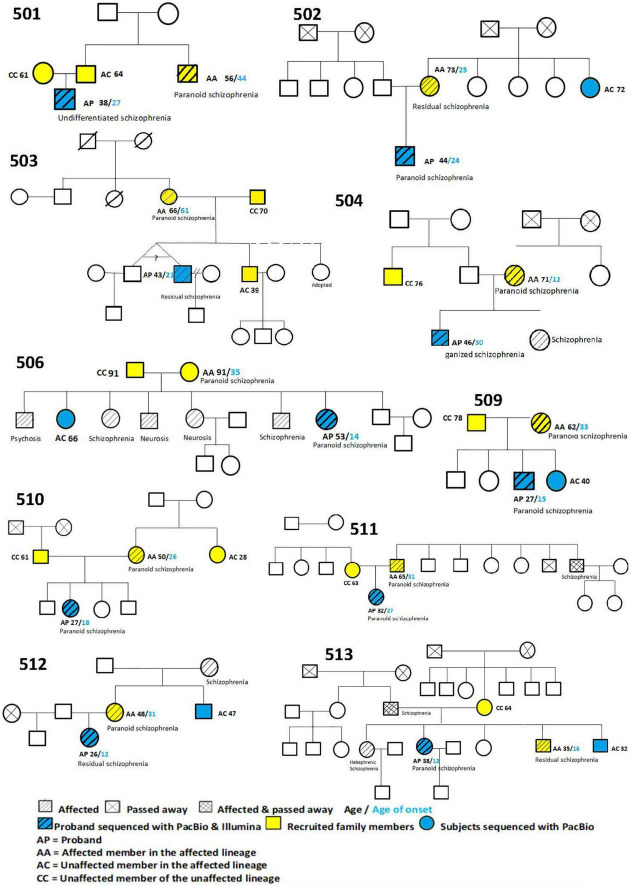
Pedigree tree of 10 probands.

We recruited 10 Chinese multiplex schizophrenia families, with 10 probands (AP), 10 affected controls from the affected lineage (AA), 8 unaffected controls from the affected lineage (AC), and 8 controls from the unaffected lineage (CC). Their pedigree trees are described in Figure 3.

### Results of symptom rating and neurocognitive tests

Symptom rating scales were administered to all recruited participants. As expected, the four groups differed significantly in both positive and negative symptoms (SAPS: *F*(3,27) = 9.85, *p* < 0.001; SANS: *F*(3,27) = 12.03, *p* < 0.001), with the AP group having significantly higher scores than the AA group, while the AC and CC groups had the lowest scores. The four groups also differed significantly in overall global functioning indicated in GAF (*F*(3,30) = 20.30, *p* < 0.001), with AC and CC groups functioning significantly better than AP and AA groups. The four groups did not differ significantly in depressive symptoms from CDSS, although there was a suggestive trend for the AA group to have higher scores than the other three groups (see details in Supplementary Table A1). One possible reason for the low scores in the AP group is the exclusion of comorbid depression in the probands.

A battery of neurocognitive tests (assessing overall cognitive functioning, auditory memory, visual memory and executive functioning, including sustained attention, divided attention, switching attention, planning and execution abilities) was administered to 22 participants, including 7 APs, 6 AAs, 6 ACs, and 3 CCs. The other participants were either too frail and/or demented to undergo or complete the testing successfully. The CC group was excluded from the analysis given the small sample size (*n* = 3). In general, AP and AA groups performed poorer than the AC group in most neurocognitive tests, as expected. However, given small group sizes and low statistical power, only performance in Processing Speed Index (assessing general cognitive efficiency) and in Colour Trail Making Interference Index (assessing sustained/divided attention) was found to be significantly different in the expected direction between the three group (*F*(2,14) = 4.70, *p* < 0.05; *F*(2,14) = 5.95, *p* < 0.05; see details in Supplementary Table A2).

### Sequencing, alignment and quality control of 15 subjects with PacBio data

PacBio sequence data on each subject contained 34.5–46.9 billion bases, of which 26.8–42.1 billion bases were mapped (Supplementary Table B). Sequencing data was judged to be of good quality, with most samples having a mapping rate above 80% and an average maximum read length above 10,000 bases. Plot-bamstats was used to parse the output statistics and call gnuplot to generate Supplementary Figures A–D.

The average coverage of all samples was greater than 10X (Supplementary Figure A). The base content of most samples is stable up to 50,000 read cycles but becomes increasingly variable beyond that, as shown in Supplementary Figure B. However, for AP2 and AC12, the base content variability begins to increase earlier, at around 40,000 read cycles, possibly because of differences in DNA quality.

All samples showed a similar distribution of indel length (Supplementary Figure C) and had GC content around 40% (Supplementary Figure D), which is consistent with the known GC content of the human genome.

#### Sequencing, alignment and quality control of 10 probands with Illumina data

Over 90% of the sequenced genome territory (2.9 billion base pairs) achieved coverage above 15X in all samples and above 20X in 6 samples (Supplementary Table C and Supplementary Figure E). Alignment quality was good, with 16.5–23.8% of the aligned bases being filtered out due to low mapping quality, low base quality, duplicates, lack of a mapped mate pair, overlapping reads or being above the capped value (Supplementary Figure F).

### Structural variation calling of 15 samples with PacBio data

**FIGURE 4:**
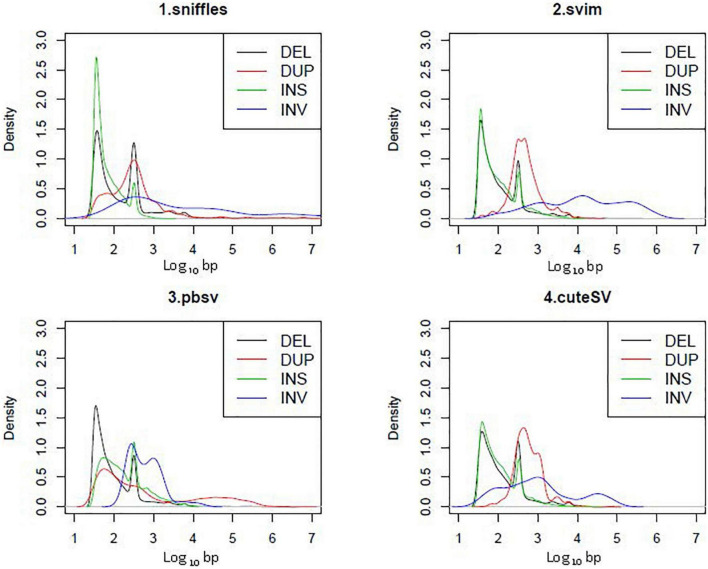
Kernel density plot in length distribution of SVs from PacBio in 15 samples.

Except for deletions, there was relatively low consistency among the four tools used for SV calling (Figure 2). The kernel density plot in Figure 4 shows the length distributions of insertions and deletions to be quite similar across samples. The insertions, deletions and duplications share the same peak at ∼300 bp (∼2.5 on the log10-scale), corresponding to the Arthrobacter luteus (Alu) retrotransposons. The deletions and duplications share another peak at ∼6,000 bp (3.8 on the log10-scale), corresponding to the L1 Homo sapiens (L1Hs) retrotransposons. The results are consistent with previous studies that the retrotransposons contribute to a large proportion of insertions, deletions and duplications in the human genome (Bennett et al., 2008; Chen et al., 2008; Cordaux and Batzer, 2010; Deininger, 2011; Sultana et al., 2019). Therefore, the SVs called with PacBio sequencing were determined to be of good quality.

### Annotation of SVs to affected genes

Applying annotSV to the consensus SVs called in the 10 probands (Figure 1), we identified 780 class-4 SVs mapped to 536 genes (Supplementary Table D). Of the SVs identified in probands, we found 692 class-4 SVs (477 mapped genes) in the 5 unaffected relatives using at least one analytical tool (Supplementary Table E). The remaining set comprising 88 class-4 SVs (79 mapped genes) was therefore identified exclusively in the 10 probands (Tables 1A, 1B). Nine of these 79 genes (*SCAPER, WWOX, ERBB4, NDUFA10, PRLR, PRKN, CHN2, CTNAP2* and *ATRX)* had SVs identified in 2 different locations within the gene. The number of SVs in each proband varied from 1 to 15 (mean 9.0). The size of SVs ranged from 32 to 12,345 bp, with a mean of 465 and median of 60 bp. By a similar workflow, we identified 404 consensus class-4 SVs in PacBio analysis of 5 unaffected relatives, which were mapped to 325 genes (Supplementary Table F). Of these, 397 SVs (320 mapped genes) were identified by at least one analytical tool in the 10 probands (Supplementary Table G). The remaining 7 SVs/genes were present in the unaffected relatives but not in the probands (Supplementary Table H).

**TABLE 1A:** 51 SVs/47 genes identified from PacBio, confirmed with Illumina, and found in 10 probands but not in 5 unaffected relatives.

Chromosome	Start	Length	Type	Sample	Gene	Location
1	55334342	52	DEL	AP2	DHCR24	intron5
1	165317168	86	DEL	AP10	LMX1A	intron3
1	197323945	41	DEL	AP10	CRB1	intron6
2	71894430	32	DEL	AP4	DYSF	intron45
2	212771238	3918	DEL	AP4	ERBB4	intron3
2	213311309	36	DEL	AP3	ERBB4	intron1
2	240861056	40	DEL	AP10	NDUFA10	intron7
2	240865209	2291	DEL	AP13	NDUFA10	intron7
3	32172207	42	DEL	AP13	GPD1L	intron2
4	26290467	4918	DEL	AP3	RBPJ	intron1
4	55577813	76	DEL	AP3	KIT	intron7
5	497823	133	DEL	AP10	SLC9A3	intron1
5	35083885	51	DEL	AP3	PRLR	intron4
5	35734503	38	DEL	AP3	SPEF2	intron21
5	138111845	193	DEL	AP4	CTNNA1	intron1
5	148385841	99	DEL	AP3	SH3TC2	intron16
6	5738544	38	DEL	AP4	FARS2	intron6
6	16367939	44	DEL	AP11	ATXN1	intron7
6	162316509	220	DEL	AP12	PRKN	intron6
7	29303576	43	DEL	AP12	CHN2	intron2
7	29399723	38	DEL	AP10	CHN2	intron3
7	33348898	99	DEL	AP10	BBS9	intron9
7	117252063	42	DEL	AP3	CFTR	intron20
7	146883956	213	DEL	AP13	CNTNAP2	intron7
7	147044513	59	DEL	AP12	CNTNAP2	intron8
9	707446	571	DEL	AP12	KANK1	intron6
9	14498762	58	DEL	AP11	NFIB	intron1
10	271571	49	DEL	AP11	ZMYND11	intron3
10	26999004	3149	DEL	AP11	PDSS1	intron5
10	57161852	38	DEL	AP6	PCDH15	intron2
10	89676313	32	DEL	AP6	PTEN	intron2
11	67965907	35	DEL	AP11	KMT5B	intron1
11	102471852	311	DEL	AP9	MMP20	intron6
11	132995568	36	DEL	AP13	OPCML	intron1
11	133964510	61	DEL	AP2	JAM3	intron1
12	21737302	34	DEL	AP12	GYS2	intron1
12	57951986	269	DEL	AP3	KIF5A	intron1
13	39379287	4132	DEL	AP11	FREM2	intron6
14	67407796	59	DEL	AP11	GPHN	intron7
15	27137546	54	DEL	AP10	GABRA5	intron6
15	38835026	39	DEL	AP4	RASGRP1	intron2
15	59431438	34	DEL	AP6	MYO1E	intron26
15	76884596	12345	DEL	AP3	SCAPER	intron23
16	21718916	39	DEL	AP4	OTOA	intron12
16	23344422	54	DEL	AP6	SCNN1B	intron1
16	78653040	57	DEL	AP9	WWOX	intron7
17	11557383	320	DEL	AP6	DNAH9	intron14
18	10944496	33	DEL	AP11	PIEZO2	intron3
18	46816093	67	DEL	AP12	DYM	intron8
X	29821294	146	DEL	AP12	IL1RAPL1	intron6
X	76881497	62	DEL	AP1	ATRX	intron19

DEL, deletion.

**TABLE 1B:** The remaining 37 SVs identified by PacBio by not confirmed by Illumina found in 10 probands but not in 5 unaffected relatives.

Chromo-some	Start	Length	Type	Sample	Gene	Location	Validated by		
							Illumina	Sanger sequencing	Integrated Genome Browser
2	55877990	138	DEL	AP6	PNPT1	intron18	UC	C	ND
6	13231402	240	DEL	AP10	PHACTR1	intron11	UC	C	ND
6	162801507	34	DEL	AP4	PRKN	intron2	UC	C	ND
7	824878	77	DEL	AP11	DNAAF5	intron12	UC	C	ND
9	138649895	133	DEL	AP11	KCNT1	intron8	UC	C	ND
10	53313464	60	DEL	AP10	PRKG1	intron3	UC	C	ND
14	100810939	38	DEL	AP4	WARS1	intron7	UC	C	ND
15	27009296	48	DEL	AP9	GABRB3	intron3	UC	C	ND
2	15519545	320	INS	AP3	NBAS	exon30-intron30	UC	C	ND
2	38913368	51	INS	AP11	GALM	intron3	UC	C	ND
2	72478232	42	INS	AP3	EXOC6B	intron20	UC	C	ND
2	74259596	74	INS	AP10	TET3	intron3	UC	C	ND
2	179621874	434	INS	AP9	TTN	exon45-intron45	UC	C	ND
3	33085270	83	INS	AP11	GLB1	intron10	UC	C	ND
3	158195587	40	INS	AP13	RSRC1	intron6	UC	C	ND
4	9952515	35	INS	AP13	SLC2A9	intron5	UC	C	ND
4	42922732	345	INS	AP12	GRXCR1	intron1	UC	C	ND
4	151293907	336	INS	AP11	LRBA	intron47	UC	C	ND
5	35074614	38	INS	AP3	PRLR	intron4	UC	UC	C
5	78253753	355	INS	AP13	ARSB	intron3	UC	C	ND
6	129828230	357	INS	AP12	LAMA2	intron61	UC	UC	C
6	157397677	346	INS	AP13	ARID1B	intron6	UC	C	ND
6	169024302	32	INS	AP10	SMOC2	intron9	UC	C	ND
7	78997174	58	INS	AP6	MAGI2	intron1	UC	C	ND
7	87098256	40	INS	AP12	ABCB4	intron3	UC	C	ND
9	97387410	326	INS	AP3	FBP1	intron1	UC	C	ND
11	66008249	329	INS	AP3	PACS1	intron21	UC	C	ND
11	77043398	173	INS	AP9	PAK1	intron14	UC	UC	C
15	51978918	123	INS	AP3	SCG3	intron3	UC	C	ND
15	76723499	93	INS	AP4	SCAPER	intron27	UC	C	ND
16	78561889	36	INS	AP11	WWOX	intron7	UC	C	ND
18	59713725	331	INS	AP3	PIGN	intron29	UC	C	ND
20	33537824	49	INS	AP4	GSS	intron2	UC	C	ND
21	39004547	350	INS	AP6	KCNJ6	intron3	UC	C	ND
22	26997611	335	INS	AP11	CRYBB1	exon5-intron5	UC	C	ND
X	32897127	39	INS	AP10	DMD	intron2	UC	C	ND
X	76906706	44	INS	AP10	ATRX	intron15	UC	C	ND

DEL, deletion; INS, insertion; C, confirmed; UC: unconfirmed; ND: not done. The sequencing data for Table 1A and this table is available in the repository: ClinVar with the accession numbers SCV002757903–SCV002757990.

To confirm that the SVs identified from PacBio are not false positives, we replicated with another sequencing platform, Illumina. We compared the identified 88 SVs which were present exclusively in the probands against the results from four SV-calling tools for Illumina data, confirming 51 SVs (47 mapped genes; Table 1A). The length of these 51 SVs has a mean of 685 and median of 57 bp. All the 29 insertion variants, and 8 of the 51 deletion variants were not confirmed by Illumina.

We set out to confirm these 29 insertions and 8 deletions by Sanger sequencing. One insertion was unable to be sequenced. All 8 deletions and 26 insertions were able to be confirmed as present by Sanger sequencing, with 2 insertions not confirmed as present. Since these SVs contain multiple repeat sequences, their confirmation by Sanger sequencing is challenging. Indeed, by performing visual manual validation with Integrated Genome Browser (IGB) on the raw PacBio and Illumina reads, we were able to confirm that all these 29 insertion and 8 deletion SVs showed evidence of an SV (Table 1B). Visualization of the one deletion and two insertions which were not confirmed by Sanger sequencing are shown in Supplementary Figure G.

### Pathway enrichment analysis

**FIGURE 5:**
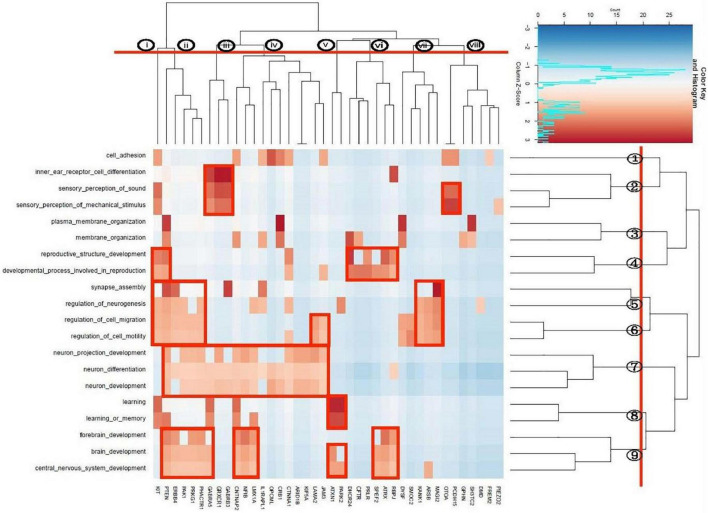
Bi-cluster analysis of enriched pathways.

We identified a total of 20 unique enriched pathways, 13 pathways in the 47-gene list annotated from case-only SVs called by both PacBio and Illumina, and 14 pathways in the 79-gene list annotated by PacBio only. Seven pathways were enriched in both lists of genes: brain development, central nervous system development, forebrain development, learning or memory, neuron development, neuron differentiation, sensory perception of mechanical stimulus. Apart from *CFTR, DHCR24, FREM2, GPHN, PRLR*, and *SH3TC2*, all genes in the enriched pathways from PacBio + Illumina were also present in the gene list from PacBio-only enriched pathways. Bi-cluster analysis was performed on these 20 unique pathways, representing the union of the two lists. This yielded a dendrogram which suggested grouping the pathways into 9 clusters (Figure 5). These were composed of a single pathway for cell adhesion (cluster 1), three hearing-related pathways (cluster 2), two pathways related to membrane organization (cluster 3), two reproduction-related pathways (cluster 4), two pathways related to synapse assembly and neurogenesis (cluster 5), two to neuronal migration (cluster 6), two to neuronal development/differentiation (cluster 7), two to learning/memory (cluster 8) and three linked to brain development (cluster 9). At a higher level of the dendrogram, clusters 5–9 coalesced to a central nervous system-related group before joining with a more peripheral-related group composed of clusters 1–4.

The gene-based dendrogram suggested a grouping of 8 gene clusters. The rectangular boxes in Figure 5 indicate bi-cluster groupings of genes and pathways. The gene cluster ii has sharing in most of the pathways related with the central nervous system. The gene cluster iii included the GABA receptors which, together with *OTOA* and *PCDH15* genes, were related to the hearing pathways. Gene cluster v (*ATXN1, PARK2*) is associated with learning and brain development, while cluster vi is related to reproduction. Gene cluster vii appears to be uniquely involved in synaptic assembly and in neuronal migration pathways.

### Longitudinal brain region enrichment analysis

**FIGURE 6:**
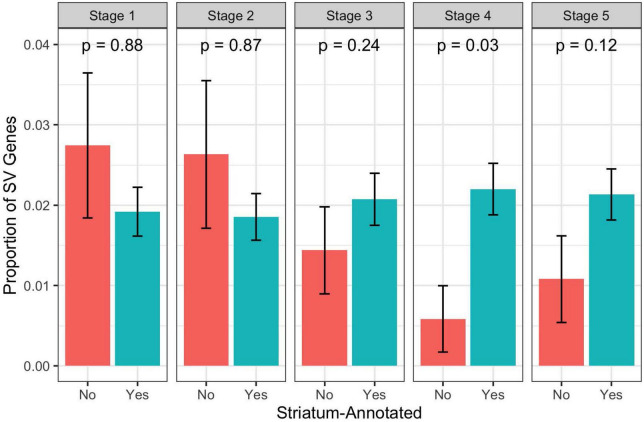
Proportion of brain expressed case-specific SV genes annotated to the striatum-expressed gene set vs non-striatum-expressed gene set across development. *P*-values are from a hypergeometric test for over-representation of case-specific SV genes in the striatum-annotated gene set. Note that these are not the competitive *p*-values as calculated by ABAEnrichment through permutation. Stage 1 = Prenatal, Stage 2 = Infant, Stage 3 = Child, Stage 4 = Adolescent, Stage 5 = Adult.

We used the ABAEnrichment package to test for enrichment of the 79 case-specific SV-containing gene set identified by PacBio among 16 human brain regions across each of 5 developmental stages sampled in the Brainspan project. From this analysis, we observed 8 region-stage combinations with significant enrichment of these case-specific SV genes, all having at least one family-wise error rate (FWER) less than 0.05. These results are summarized in Table 2. The strongest evidence for enrichment appeared in the striatum during adolescent development, with 3 out of 5 quantile cutoffs indicating a FWER less than 0.05 (minimum FWER = 0.0063, for quantile cutoff 0.5). Figure 6 shows the proportion of brain-expressed case-specific genes annotated to the striatum-expressed gene set vs non-striatum expressed across development using the 0.5 quantile cutoff identified by ABAEnrichment. The cerebral nuclei region was found to be enriched at one quantile cutoff for both prenatal and adolescent development. The remaining regions showed significant enrichment during prenatal development, each with a single quantile cutoff value resulting in an FWER < 0.05. These regions include the posterior superior temporal cortex, the hippocampus, the primary motor and somatosensory cortices, as well as the frontal neocortex.

**TABLE 2:** ABAEnrichment in region-stage combination for SV containing genes identified by PacBio sequencing.

			FWER From expression quantile				
Region	Stage	N signif.	0.5	0.6	0.7	0.8	0.9
Frontal neocortex	Prenatal	1	0.920	0.791	**0.050**	0.116	0.542
Primary motor cortex (area M1, area 4)	Prenatal	1	0.764	0.632	0.107	**0.044**	0.291
Primary somatosensory cortex (area S1, areas 3,1,2)	Prenatal	1	0.943	0.446	**0.046**	0.055	0.635
Posterior (caudal) superior temporal cortex (area 22c)	Prenatal	1	0.905	0.610	**0.018**	0.229	0.961
Hippocampus (hippocampal formation)	Prenatal	1	0.732	0.108	0.664	**0.036**	0.960
Cerebral nuclei	Prenatal	1	0.855	0.294	0.179	**0.020**	0.947
Cerebral nuclei	Adolescent	1	0.326	0.442	**0.037**	0.836	0.886
Striatum	Adolescent	3	**0.006**	**0.017**	**0.012**	0.689	0.757

This table details the 8 region-stage combinations identified by ABAEnrichment as being significantly enriched for the case-specific SV genes identified by PacBio sequencing. Regions significant with a familywise-error rate (FWER) less than 0.05 using a given expression quantile cutoff for annotating genes to brain regions are bolded. N signif. represents the number of expression quantile cutoffs for which the FWER for a given region-stage combination was less than 0.05.

## Discussion

### On phenotype and research design

Schizophrenia is a polygenic disorder influenced by both genetic and environmental factors, with most studies to date focused on identifying CVs, rare variants, and large SVs. By using PacBio sequencing, we increase our likelihood of detecting rare medium-sized SVs with moderate- to high-penetrance that have arisen relatively recently in evolutionary time.

We recruited a relatively homogeneous group of patients suffering from chronic schizophrenia, having negative symptoms indicated by a high SANS score and impairment of psychosocial functioning evidenced by a low GAF score. It is unlikely that patients’ negative symptoms were the result of depressive symptoms, given low ratings on the CDSS. The neurocognitive battery of tests also indicated that affected subjects performed poorly compared to their unaffected family members. By selecting patients at the severe end of the schizophrenia spectrum in multiplex families, we increased the power to detect genetic risk variants.

### On genes and SVs

To ensure that the SVs identified from PacBio are replicable, we performed Illumina sequencing on the 10 probands as a technical confirmation. Illumina validated 51 of the 88 SVs (or 47 of the 79 annotated genes) that were detected exclusively in probands from PacBio sequencing. These 51 SVs represent the highest-confidence set of SVs conferring genetic risk for schizophrenia. However, considering that Illumina is less sensitive than PacBio in detecting SVs, the set of 51 SVs may be overly conservative. Indeed, Sanger sequencing has validated 34 (all deletions and 26 of 29 insertions) and manual inspection of PacBio data confirmed all the remaining SVs.

The majority of SVs identified were located in introns. Only 3 of the 88 SVs in the probands involved exons, specifically, those of the *NBAS, TTN* and *CRYBB1* genes. Similarly, we found that exons were involved in only 15 genes from the list of 780 SVs (Supplementary Table D), and 7 genes from the 404-SV list (Supplementary Table F). Thus, most of the identified SVs do not change the primary protein structure, but may instead regulate expression of the identified genes. Other epigenomic studies (Keverne et al., 2015; Focking et al., 2019; Kuehner et al., 2019) have identified many non-coding elements involved in regulating gene expression underlying neurogenesis, cell differentiation and neurodevelopment.

17 of the list of 79 genes that were found in 10 probands but not in 5 unaffected relatives (Tables 1A, B) have been reported in the list of 1,179 genes associated with the schizophrenia phenotype in GWASdb (Rouillard et al., 2016). These include *ATXN1, CHN2, CNTNAP2, CRYBB1, DMD, DYSF, ERBB4, GABRA5, GABRB3, IL1RAPL1, K1F5A, MAGI2, OPCML, RASGRP1, RBP1, SLC2A9 and WWOX.* Additionally, different members of some gene families are present in the two lists. For example, genes from the protocadherin and dynein-axonemal heavy chain families were present in both our 79 gene list (*PCDH15* and *DNAH9)* and in the GWASdb list *(PCDH7/PCDH9* and *DNAH5).*

The list of 528 genes, which includes genes found in probands and unaffected relatives, are more difficult to interpret as many of these are likely to be unrelated to schizophrenia in the families, as explained in the Limitations section. Nevertheless, we found a number of genes in this list that have a reported association to schizophrenia or other neuropsychiatric disorders, for example, *TENM4* (Xue et al., 2019), *NXN* (Lachman, 2009), *NRXN1* (Kirov et al., 2009), *GALNT5* (Narayan et al., 2009), *SHANK3* (de Sena Cortabitarte et al., 2017) and *RELN* (Abdolmaleky et al., 2005). This list also contains the *FOXP1* (Lozano et al., 2015) and *FOXP2* (Lai et al., 2003) genes, which are required for development of speech and language in humans and interact with *CNTNAP2* (Ji et al., 2013), a member of the 47-gene list which is in the neurexin family of genes having known association with schizophrenia. Thus, it is possible that some of these 528 genes are contributing to Schizophrenia risk in these families, but their effect sizes are likely to be modest given that they were also observed in unaffected relatives.

### On biological pathways

We have identified multiple genes containing SVs that are possibly related to schizophrenia. Genes work together in synchronized biological processes, thus we sought to identify the biological pathways in which these genes operate. Previous GWAS have implicated histone methylation process, multiple immune and neuronal signaling pathways and postsynaptic density in schizophrenia, major depression and bipolar disorder (O’Dushlaine et al., 2015) and the postsynptic signaling processes particularly dopaminergic and cholinergic synapses (Schijven et al., 2018).

Consistent with these previous findings, we identified brain-related pathways that were significantly enriched in the SV-linked genes present in the probands, including neuron differentiation, migration and brain development. These findings lend further support to the neurodevelopmental hypothesis of schizophrenia including both prenatal development and later maturation of the brain.

Three pathways involving hearing (inner ear receptor cell differentiation, sensory perception of sound and sensory perception of mechanical stimulus) were enriched in our identified SV gene set. Previous epidemiological evidence has shown hearing impairment to be associated with auditory hallucinations, cognitive dysfunction, and psychosis (Linszen et al., 2019). Therefore genetic factors related to hearing impairment may also contribute to risk of schizophrenia. Some previous studies have identified genes associated with auditory hallucinations, but none overlap with the proband-specific SV-containing genes identified in this study (Hugdahl et al., 2008; Shao et al., 2021). Furthermore, GWAS of common variants have found no evidence for enrichment of auditory-related pathways in schizophrenia (Trubetskoy et al., 2022).

Interestingly, we did not find evidence for enrichment of immunological pathways. This may be because our phenotype is targeted at severe and familial cases. Indeed, the genetic correlations between schizophrenia and immunological disorders are inconsistent and often contradictory (Birnbaum and Weinberger, 2020). Some have hypothesized that there is heterogeneity in the etiology of schizophrenia such that some severe early-onset cases may be more neurodevelopmentally-related, whereas late-onset cases are likely to be more highly influenced by immunological processes affecting microglial functions (Monji et al., 2013). If a pathway is found enriched for the identified genes, other genes bundled in the same pathway are also candidates for being involved in the biological mechanisms of schizophrenia. We suggest that further research into the role of other genes in each pathway is needed to elucidate the pathophysiological mechanisms, and the symptoms that each gene/pathway confers. We also suggest that further work would be needed to identify the regulatory regions of the identified genes, and their effect on gene expression in a spatiotemporal manner, in specific cell types at various developmental periods in different brain locations. Given evidence of high comorbidity and genetic correlation across psychiatric disorders, we might expect a high degree of overlap between our identified risk gene list and the set of highly pleiotropic loci identified in a recent analysis from the PGC (Cross-Disorder Group of the Psychiatric Genomics Consortium, 2019). There were no genes which overlapped with our proband-specific gene list, although there were genes among the highly pleiotropic loci from the same gene family (CTNN and KCN) as contained in our proband-specific set of risk genes. However, these gene families are very large, which increases the chance of random overlap. This relatively low overlap suggests that our approach of sampling severe familial schizophrenia cases has identified risk variants that are likely to be more specific to schizophrenia, rather than conferring general risk for psychiatric disorders.

### On brain region enrichment

Our ABA Enrichment analysis indicated strong evidence for enrichment of case-only SV genes in those highly expressed in the striatum during adolescent development. This is highly consistent with decades of convergent evidence supporting a role for both structural and functional striatum abnormalities in the pathophysiology of SCZ (McCutcheon et al., 2019). Specifically, dysregulated striatal dopaminergic signaling is strongly linked with positive symptoms, but has also been suggested to contribute to cognitive deficits as well. Furthermore, adolescence has long been hypothesized as a pivotal developmental period in the etiology of SCZ (Gomes et al., 2016), with adolescents of high genetic risk for psychosis showing specific deficits in striatal activation (Diwadkar et al., 2012; Vink et al., 2016; Hubl et al., 2018).

Recently, large GWAS of SCZ have also provided some evidence of enrichment for common risk variants in striatal interneurons (Ripke et al., 2020). Additional studies using SCZ polygenic risk scores (PGS) have demonstrated associations between PGS and diminished striatal activity during reward processing in healthy adults (Lancaster et al., 2019) and adolescents (Lancaster et al., 2016). One previous WGS study of SCZ patients identified a handful of large SVs in genes which are significantly differentially expressed in the striatum (Tang et al., 2017). Thus, our results indicate that in addition to CV and large SV contributions to SCZ through effects on striatal function, rare medium-sized SVs may also contribute to striatal deficits, specifically during adolescence.

### Limitations and future directions

Our sample size is necessarily small, given the current high cost of third-generation sequencing. Therefore, our findings should be viewed with caution, and require replication and confirmation by others. Nevertheless, our initial results are encouraging, working to fill the knowledge gap between small and very large SVs conferring risk for schizophrenia. Larger studies using long-read sequencing on both affected and unaffected family members are likely to identify or confirm additional medium-sized risk SVs, providing a more comprehensive view of the genetic risk landscape of schizophrenia.

The gene set of only 7 genes in the unaffected controls is too small for comparison with the gene set of 79 genes in the probands. Thus we structured our analysis on PacBio data by generating multiple lists with different combinations of tools on either the probands or the relatives. In the future, statistical power can be improved by sequencing more unaffected controls. In the present analysis, we can only contrast the SVs which were present in the probands to SVs present in both affected and unaffected subjects. This comparison is not ideal, since some SVs identified in the controls may also be related to the disorder; conversely, some of the SVs identified in the probands may be false positives. Although common variants explain only up to 20% of the variability of schizophrenia (Bigdeli et al., 2020), our sample of 10 families is not big enough to estimate the effect sizes of medium-size structural variants or their contribution to missing heritability.

Further bioinformatic and molecular studies of the identified genome regions are needed to confirm the effects of SVs on gene expression and elucidate the biological mechanisms involved in the development of schizophrenia. While there have been systematic studies of the impact of SVs on gene expression (Chiang et al., 2017), bioinformatic functional analysis of SVs is hampered by the lack of publicly available databases linking SVs to gene expression on a genome wide scale.

We have not performed detailed functional analyses of the impact of identified SVs on their target genes and pathways. Possible functional analyses include knock-out, knock-in or disruption of the implicated genes in a cell system or animal model, to look for changes in gene expression levels in biological pathways, and downstream phenotypic changes. Our enrichment analysis has provided clues to the pathways involved in the development of schizophrenia. Future studies on the effects of dysregulation of these pathways on brain development and neuronal function may provide biological insights into the etiology of chronic schizophrenia, and identify biomarkers and targets for interventions.

## Conclusion

Medium-sized intronic SVs were detected in schizophrenia patients with positive family history and chronic deficit symptoms using long-read sequencing. Identified SVs implicate multiple genes and pathways important in brain development and function, with likely involvement in schizophrenia pathogenesis. Until now, such medium-sized SVs have been under-studied, as they are difficult to detect by conventional SNP arrays or sequencing technology, but nevertheless may explain part of the missing heritability of schizophrenia.

## Data availability statement

The datasets presented in this study can be found in online repositories. The names of the repository/repositories and accession number(s) can be found in the article/Supplementary material.

## Ethics statement

The studies involving human participants were reviewed and approved by Kowloon West Cluster Research Ethics Committee, Hospital Authority, Hong Kong. The patients/participants provided their written informed consent to participate in this study.

## Author contributions

CL, SL, SC, KW, PC, and LC: fieldwork. CL, PL, PS, RY, JT, LB, and YZ: data analysis and drafting of manuscript. All authors the initial conceptualization and design/planning of the study and editing and final approval of manuscript.
