# Supplementary material for: Third-generation genome sequencing implicates medium-sized structural variants in chronic schizophrenia
Source: Front Neurosci. 2023 Jan 11;16:1058359. doi: 10.3389/fnins.2022.1058359 (PMC9874699; doi:10.3389/fnins.2022.1058359)
Supplement: Supplementary file 1 [file Data_Sheet_1.pdf]

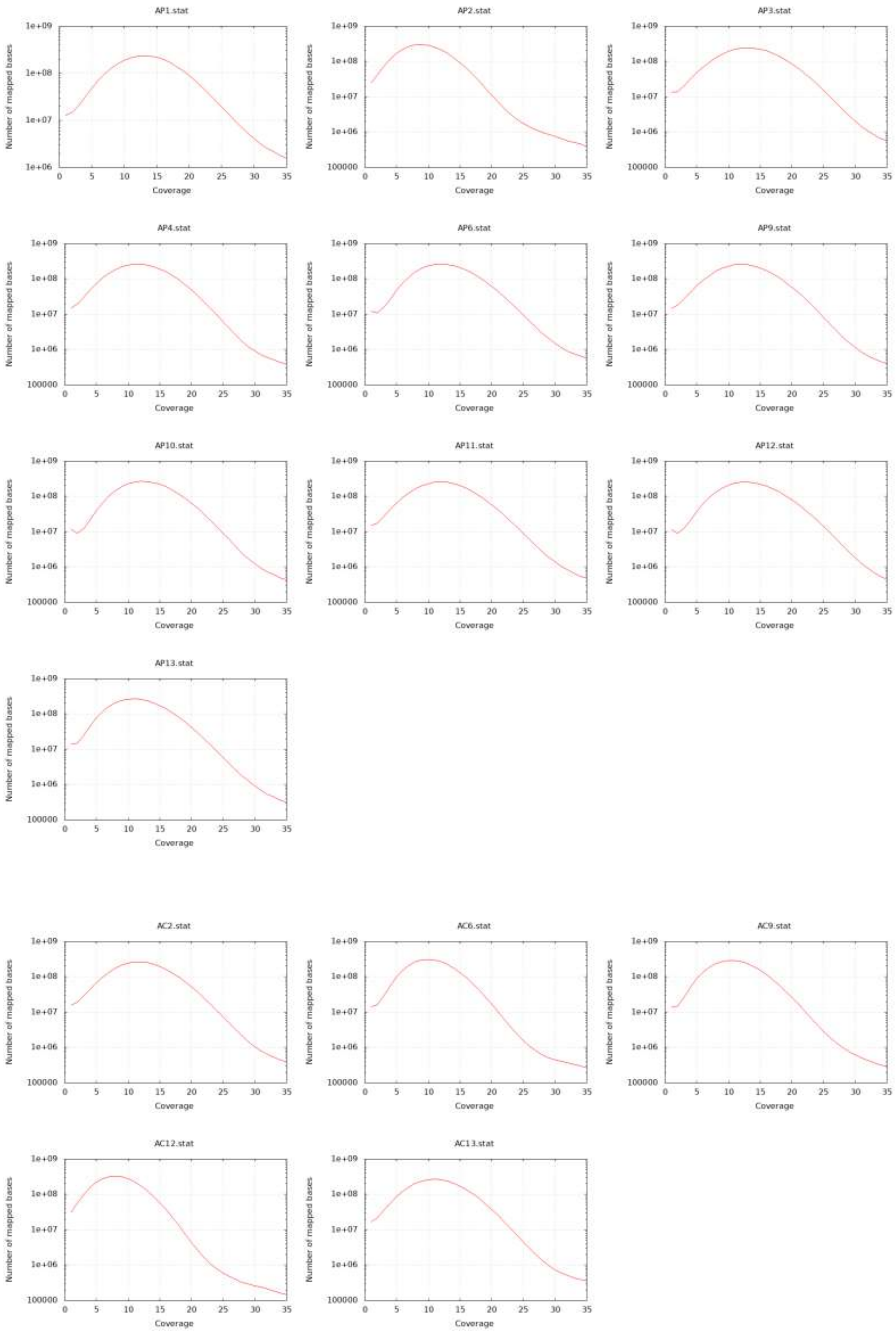

Supplementary Figure A: Coverage distribution of 10 probands (top) and 5 unaffected relatives (bottom)

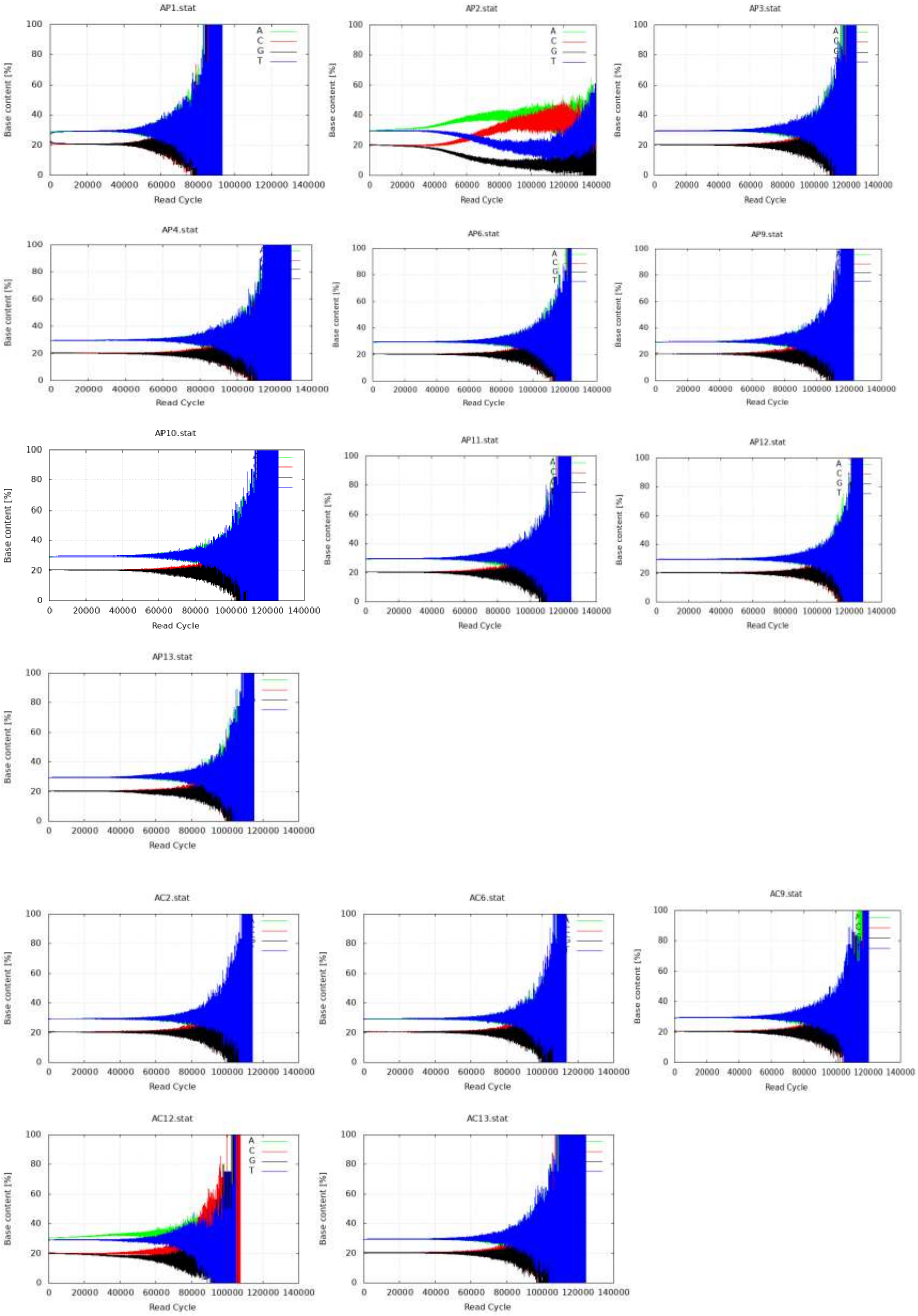

Supplementary Figure B: Base content on reads of 10 probands (top) and 5 unaffected relatives (bottom)

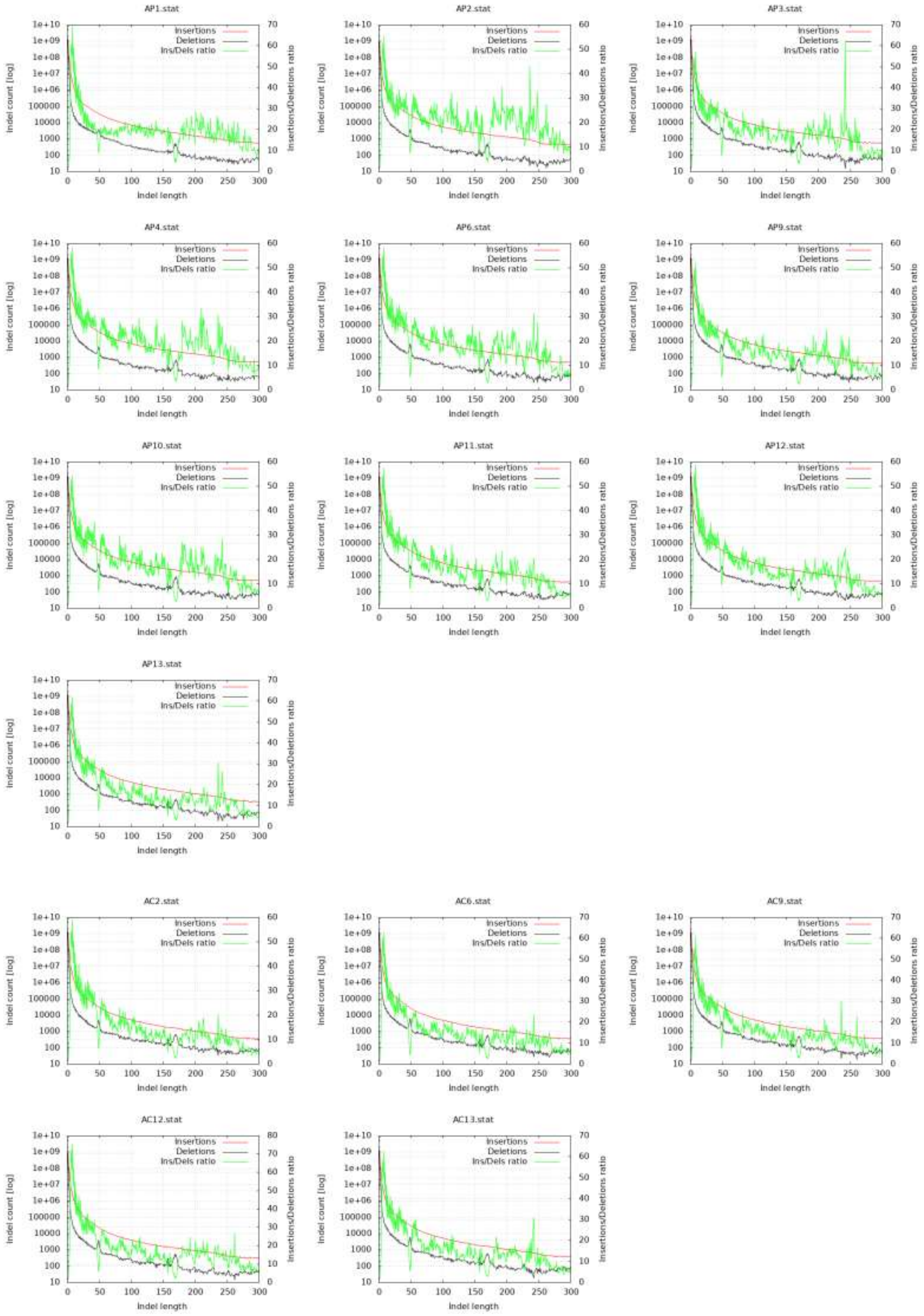

Supplementary Figure C: Counts of Indel length on reads of 10 probands (top) and 5 unaffected relatives (bottom)

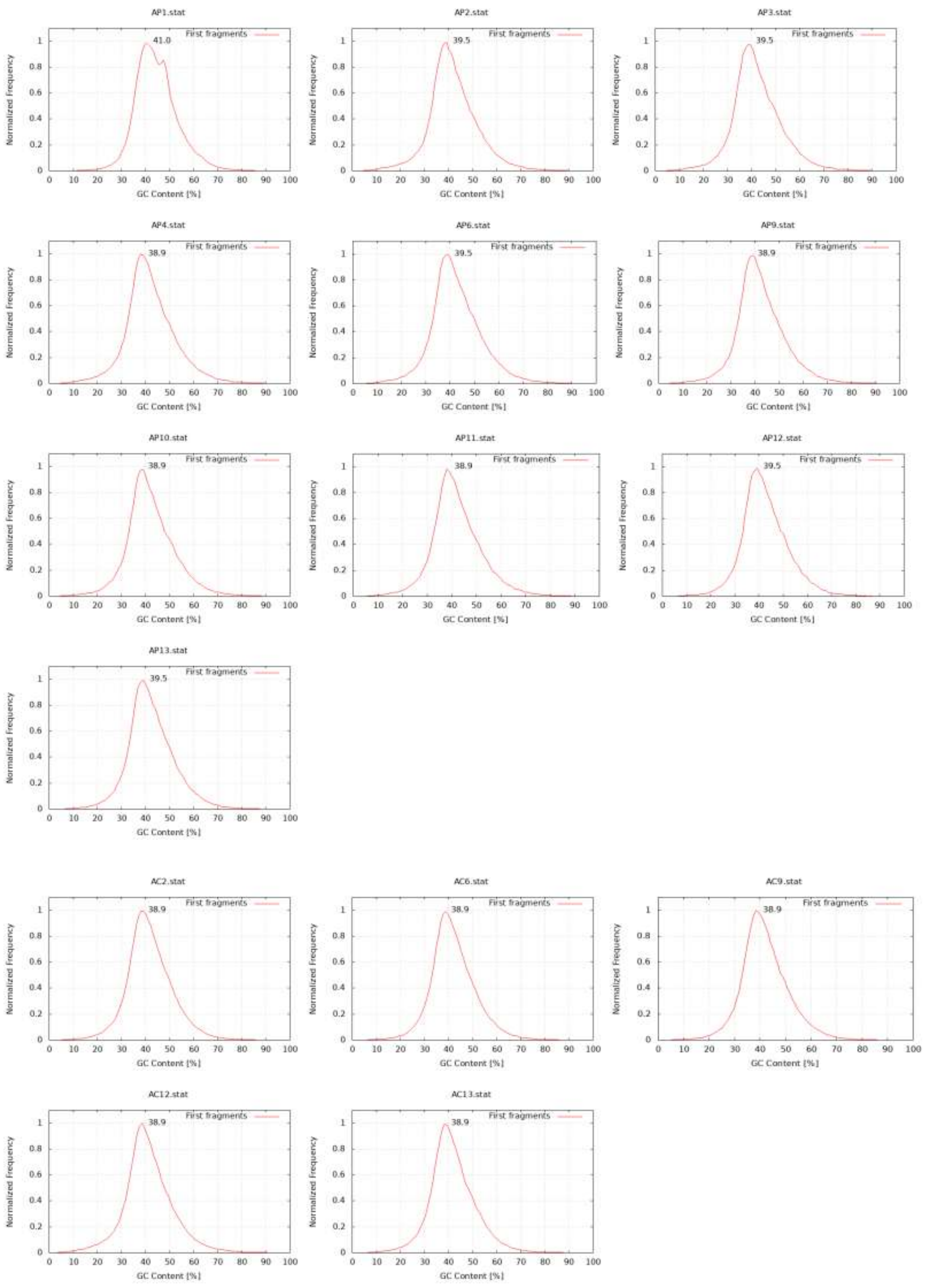

Supplementary Figure D: Distribution of GC content of 10 probands (top) and 5 unaffected relatives (bottom)

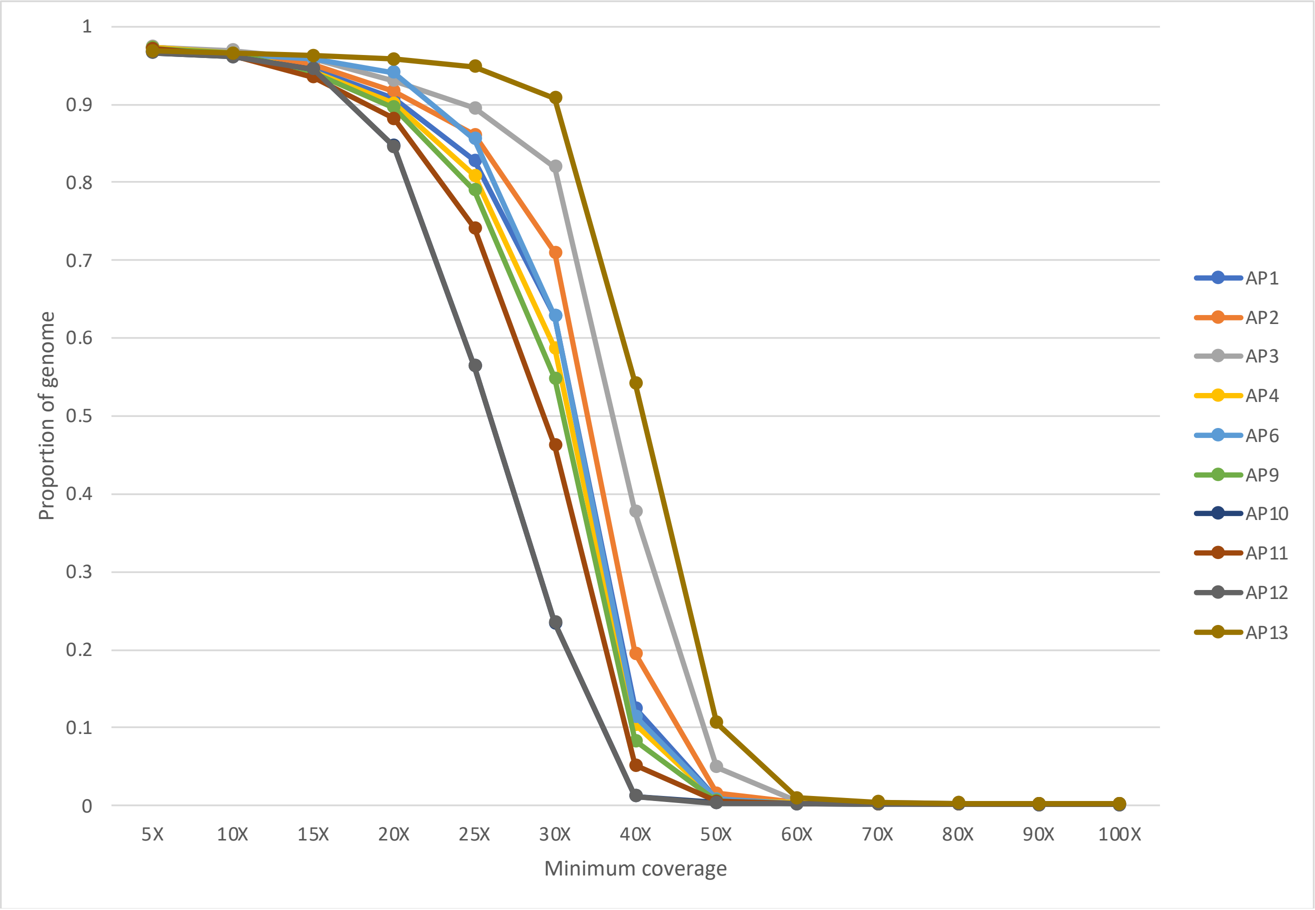

Supplementary Figure E: Proportion of genome exceedingminimum coverages in the Illumina data of 10 probands

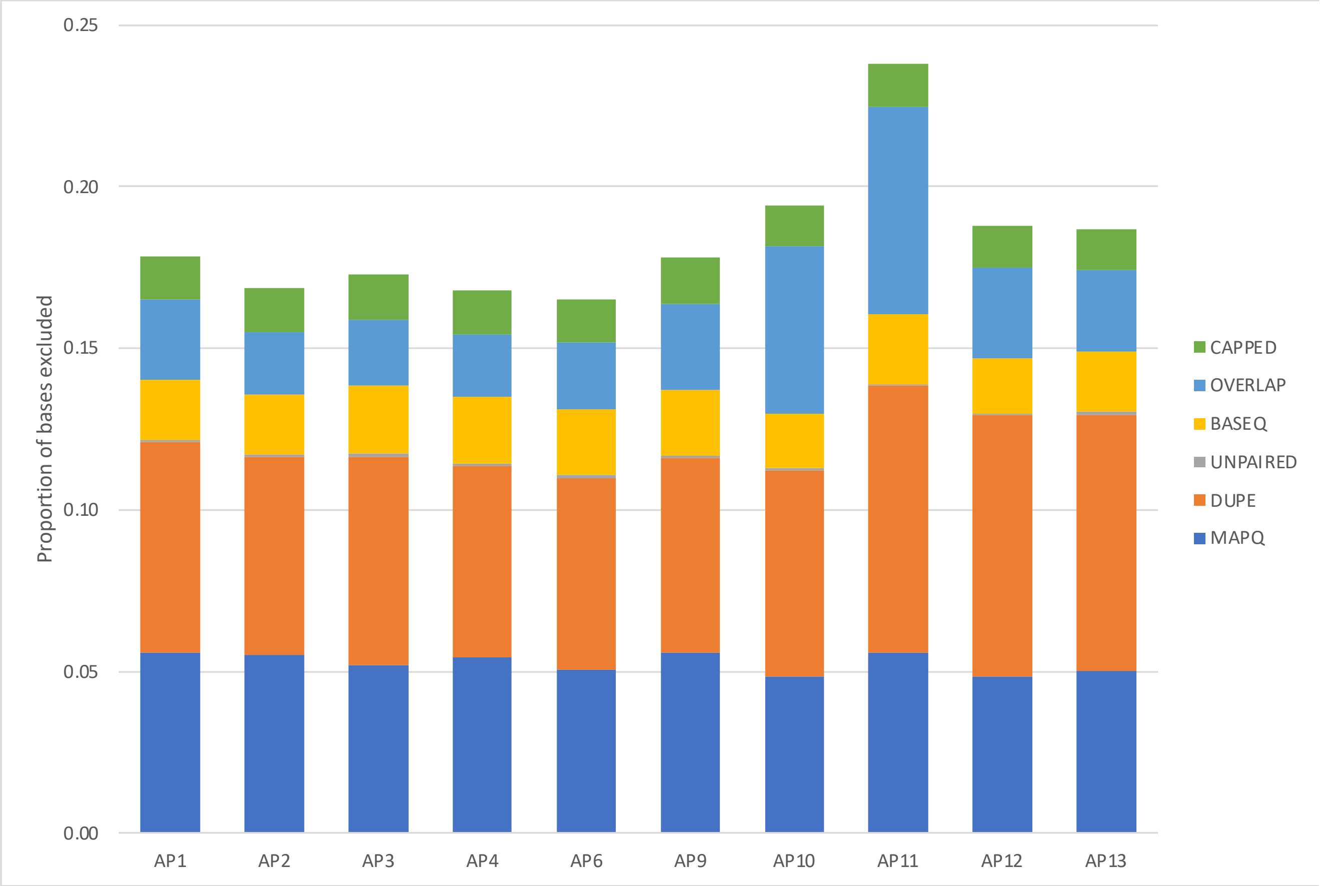

Legends:

The fractions of aligned bases that were filtered out because they were

MAPQ: in reads of low mapping quality (default is > 20)

DUPE: in reads marked as duplicates

UNPAIRED: in reads without a mapped mate pair

BASEQ: of low base quality (default is <20)

Capped would have raised coverage above the capped value (default cap = 250x)

Supplementary Figure F: Proportion of bases excluded in the Illumina data of 10 probands

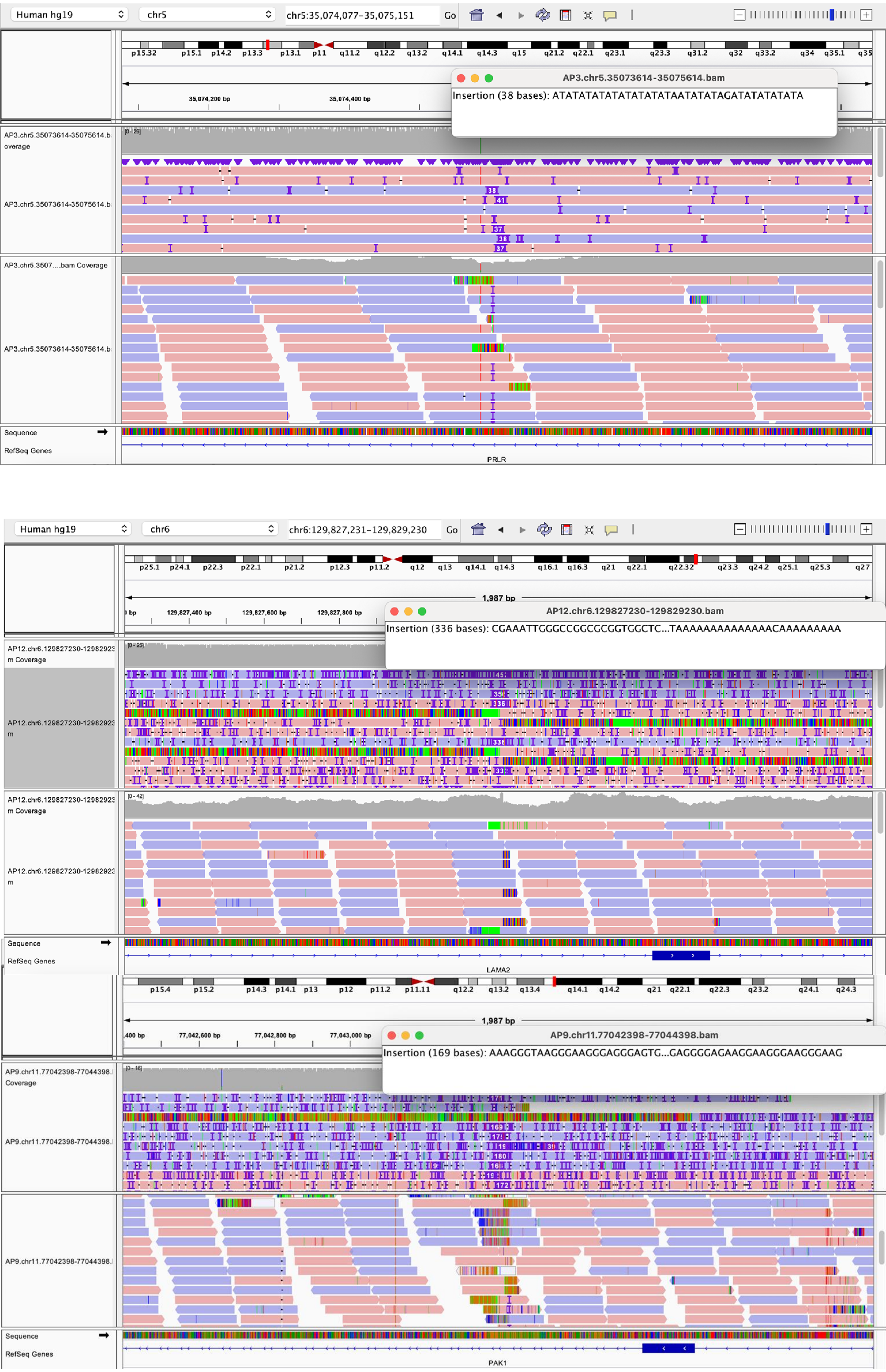

Supplementary Figure G: Visual Manual validation displayed from Integrated Genome Browser of the 3 SVs that were called by PacBio but not validated by Illumina and Sanger Sequencing

Supplementary Table A1: Results of symptom rating and functioning on schizophrenia and depression in schizophrenia

Rating scales	Group			
	**AP mean (SD)**	**AA mean (SD)**	**AC mean (SD)**	**CC mean (SD)**
**SAPS*** **	24.00 (15.01)	11.40 (9.08)	0.50 (1.22)	0.29 (0.75)
**SANS*** **	46.90 (28.41)	21.80 (15.81)	1.83 (2.99)	0.00 (0.00)
**GAF*** **	47.70(18.80)	64.50(15.17)	85.00(2.67)	87.25(2.49)
**CDSS**	0.90 (1.72)	2.40 (2.95)	0.33 (0.51)	0.00 (0.00)

ANCOVA with age and gender as covariates, ***p< 0.001

AP: Proband at the affected side of the family, AA: Affected control in the affected side of the family, AC: Unaffected control in the affected side of the family, CC: Unaffected control in the unaffected side of the family; SAPS: Scale for the Assessment of Positive Symptoms, SANS: Scale for the Assessment of Negative Symptoms, GAF: Global Assessment of Functioning Scale, CDSS: Calgary Depression Scale for Schizophrenia

Supplementary Table A2: Results of neurocognitive tests

Neurocognitive Measure	Subscale	Group		
		AP Mean (SD)	AA Mean (SD)	AC Mean (SD)
WAIS-IV (HK)	Verbal Comprehension Index	92.86 (17.22)	86.33 (10.29)	105.83 (17.76)
	Perceptual Reasoning Index	87.14 (10.81)	84.17 (8.35)	102.67 (13.74)
	Working Memory Index	82.57 (7.50)	83.17 (15.09)	97.83 (14.09)
	Processing Speed Index*	79.14 (4.56)	87.33 (10.27)	96.00 (7.01)
	Full Scale IQ	84.71 (10.00)	84.17 (9.45)	100.83 (14.11)
HKLLT	Total Learning	-1.77(0.99)	-1.75 (0.83)	-0.94 (1.60)
	Learning Slope	-0.44 (1.20)	-0.38 (0.92)	-0.54 (0.64)
	Immediate Recall	-2.27 (2.20)	-1.58 (1.19)	-1.16 (1.91)
	Delay Recall	-2.38(2.16)	-1.40 (1.11)	-1.15(1.72)
	Recognition - Discrimination Score	-4.11(4.93)	-2.27 (1.21)	-2.23 (4.26)
RCFT	Immediate Recall	34.38 (15.65)	38.17 (16.04)	43.25 (19.57)
	Immediate Recall (%ile)	19.19 (30.16)	25.75 (36.88)	39.38 (40.36)
	Delayed Recall	35.00 (18.12)	33.50 (19.08)	43.00 (18.94)
	Delayed Recall (%ile)	22.50 (33.64)	22.92 (36.25)	38.38 (38.55)
	Recognition Total Correct (%ile range)	13.19 (18.64)	14.67 (15.51)	23.50 (21.93)
CTT	Interference Index*	-1.84 (1.22)	-0.10 (1.11)	0.10 (0.87)
Stroop	Interference Index	-0.67 (3.83)	0.87 (3.20)	0.65 (1.76)
DVT	Total Time	-9.86 (25.07)	-1.84 (8.87)	-2.00 (11.31)
Verbal Fluency	Production	-1.65 (0.98)	-1.58 (1.23)	-0.84 (0.87)
TOL	Total Problem Solving Time	72.33 (15.77)	63.14 (45.62)	94.00 (14.07)

ANCOVA with age and gender as covariates, * $p < 0.05$

AP: Proband at the affected side of the family, AA: Affected control in the affected side of the family, AC: Unaffected control in the affected side of the family; WAIS-IV (HK): Wechsler's Adult Intelligence Scale (Forth Edition) Hong Kong Version, HKLLT: Hong Kong List Learning Test, RCFT: Rey-Complex Figure Test, Stroop: Stroop Test, Verbal Fluency, CTT: Colour Trail Test, DVT: Digit Vigilance Test, TOL: Tower of London Test

Supplementary Table B: Sequencing data from PacBio

Sample	Total length	Bases mapped	Bases mapped (cigar)	Mapping rate(cigar)	Error rate	Average length	Maximum length
AP1	37405195855	33871812659	31748531882	84.9%	15.1%	9920	93109
AP2	34542825531	31536222719	29673556309	85.9%	12.4%	10743	187336
AP3	46990263827	44169815256	42143363264	89.7%	12.2%	16566	126452
AP4	42621033452	39603549209	37525785763	88.0%	12.3%	15238	128937
AP6	44460957306	41627279422	39704240287	89.3%	12.6%	15541	123993
AP9	43156103684	40632279296	38912524748	90.2%	12.1%	16277	123019
AP10	44528388653	41959445577	40141016239	90.1%	11.8%	15912	125856
AP11	43676264363	40736159261	38844657055	88.9%	11.9%	14889	124850
AP12	46914274375	43847334140	41622173799	88.7%	12.1%	17676	128487
AP13	42019422006	38496428831	36256155465	86.3%	12.2%	14441	115001
AC2	45336327972	40765618375	38247706218	84.4%	12.4%	12787	114071
AC6	38233291717	35026146445	32978676161	86.3%	12.5%	14397	113522
AC9	39818597577	36577832840	34376331575	86.3%	12.3%	13803	120233
AC12	34531699528	29464477854	26840618130	77.7%	13.0%	9529	107294
AC13	42568438088	38434406146	36102092668	84.8%	12.6%	13353	124333

Supplementary Table C: Alignment statistics from Illumina sequencing of 10 probands

									% of genome with coverage exceeding												
Sample	Coverage (%) : Mean (SD, Median, MAD)	MAPQ (%)	DUPE (%)	Unpaired (%)	BASEQ (%)	Overlapped (%)	Capped (%)	Total (%)	5X	10X	15X	20X	25X	30X	40X	50X	60X	70X	80X	90X	100X
AP1	30.95 (10.67 , 32 , 5)	5.6	6.5	0.1	1.9	2.5	1.3	17.9	97.3	96.7	94.5	90.7	82.8	62.8	12.5	0.9	0.4	0.3	0.2	0.2	0.1
AP2	32.67 (11.10 , 34 , 5)	5.5	6.1	0.1	1.8	1.9	1.4	16.9	97.3	96.8	95.0	91.7	86.0	71.0	19.5	1.6	0.4	0.3	0.2	0.2	0.2
AP3	36.13 (11.93 , 37 , 5)	5.2	6.4	0.1	2.1	2.0	1.4	17.3	97.3	96.9	95.8	93.1	89.5	82.0	37.7	5.0	0.6	0.4	0.3	0.2	0.2
AP4	30.25 (10.56 , 31 , 5)	5.4	5.9	0.1	2.1	1.9	1.4	16.8	97.3	96.6	94.2	90.1	80.8	58.7	10.4	0.8	0.3	0.3	0.2	0.2	0.1
AP6	31.11 (10.22 , 32 , 4)	5.1	5.9	0.1	2.1	2.1	1.3	16.5	96.7	96.4	95.8	94.1	85.6	62.8	11.4	0.8	0.3	0.3	0.2	0.2	0.1
AP9	29.55 (10.28 , 30 , 5)	5.6	6.0	0.1	2.0	2.7	1.4	17.8	97.2	96.5	94.0	89.6	79.0	54.8	8.3	0.6	0.3	0.2	0.2	0.2	0.1
AP10	25.06 (8.73 , 25 , 4)	4.8	6.4	0.1	1.7	5.2	1.3	19.4	96.6	96.1	94.5	84.6	56.4	23.4	1.2	0.3	0.2	0.2	0.1	0.1	0.1
AP11	28.20 (9.99 , 29 , 4)	5.6	8.3	0.1	2.2	6.4	1.3	23.8	97.2	96.3	93.4	88.2	74.1	46.2	5.2	0.5	0.3	0.2	0.2	0.1	0.1
AP12	25.06 (8.70 , 25 , 4)	4.8	8.1	0.1	1.7	2.8	1.3	18.8	96.6	96.1	94.5	84.6	56.4	23.5	1.2	0.3	0.2	0.2	0.1	0.1	0.1
AP13	39.50 (12.13 , 40 , 5)	5.0	7.9	0.1	1.9	2.5	1.3	18.7	96.8	96.5	96.2	95.8	94.8	90.8	54.2	10.6	1.0	0.4	0.3	0.3	0.2

Footnote : Genome Territory : 2897310547 base pairs
MAD: Median Absolute Deviation
MAPQ: in reads marked as poor Mapping Quality
DUPE : in reads marked as duplicates
Unpaired : in reads without a mapped mate pair
BASEQ : in reads of low base quality (default is <20)

Supplementary Table D: 780 class-4 SVs and 536 genes found by 4 tools in 10 probands

Chromosome	Start	End	Length	Type	Sample	Gene	Transcript	Location
15	67534470	67534807	337	DEL	AP10,AP11,AP13,AP4,AP9	AAGAB	NM_024666	intron1-intron1
7	87098256	87098296	40	INS	AP12	ABCB4	NM_000443	intron3-intron3
10	101587738	101587964	226	INS	AP11,AP13,AP3	ABCC2	NM_000392	intron19-intron19
12	22016215	22016262	47	DEL	AP10,AP12,AP3	ABCC9	NM_020297	intron17-intron17
3	43735559	43735607	48	INS	AP6	ABHD5	NM_001365650	intron1-intron1
17	61565898	61566202	304	INS	AP10	ACE	NM_000789	intron15-intron16
2	74128664	74128709	45	DEL	AP9	ACTG2	NM_001199893	intron2-intron2
1	236900604	236900639	35	INS	AP10	ACTN2	NM_001103	intron9-intron9
2	158719091	158719404	313	DEL	AP10,AP11,AP12,AP3,AP6	ACVR1	NM_001105	intron1-intron1
12	52354757	52354890	133	DEL	AP11	ACVR1B	NM_004302	intron1-intron1
22	17665788	17665851	63	DEL	AP10,AP4,AP9	ADA2	NM_177405	intron4-intron4
15	58912854	58913175	321	DEL	AP1,AP10,AP11,AP12,AP13,AP2,AP3,AP4,AP6,AP9	ADAM10	NM_001110	intron11-intron11
8	38953618	38953877	259	DEL	AP4	ADAM9	NM_003816	intron20-intron20
15	100606978	100607180	202	DEL	AP11,AP3	ADAMTS17	NM_139057	intron15-intron15
15	100693156	100693198	42	INS	AP12,AP13	ADAMTS17	NM_139057	intron9-intron9
15	100702654	100702717	63	DEL	AP1	ADAMTS17	NM_139057	intron8-intron8
15	100879781	100879822	41	DEL	AP12	ADAMTS17	NM_139057	intron2-intron2
9	136414109	136414433	324	DEL	AP10,AP11	ADAMTSL2	NM_001145320	intron9-intron9
5	89860425	89860752	327	DEL	AP10,AP11,AP3,AP4,AP9	ADGRV1	NM_032119	intron1-intron1
5	89997830	89998001	171	INS	AP13	ADGRV1	NM_032119	intron34-intron34
10	76230334	76230481	147	INS	AP10	ADK	NM_001202450	intron6-intron6
5	132239876	132239918	42	DEL	AP9	AFF4	NM_014423	intron6-intron6
18	12331807	12331849	42	DEL	AP1	AFG3L2	NM_006796	intron16-intron16
15	86829966	86830016	50	INS	AP2	AGBL1	NM_152336	intron16-intron16
15	87177015	87177098	83	DEL	AP10,AP3	AGBL1	NM_152336	intron22-intron22
2	178287637	178288051	414	DEL	AP12,AP13,AP3,AP4,AP6	AGPS	NM_003659	intron2-intron2
2	178315691	178315735	44	INS	AP10,AP3,AP4,AP6	AGPS	NM_003659	intron8-intron8
2	178318718	178319078	360	INS	AP3	AGPS	NM_003659	intron8-intron8
2	178351456	178351821	365	INS	AP10	AGPS	NM_003659	intron11-intron11
1	966280	966317	37	DEL	AP6	AGRN	NM_001305275	intron2-intron2
1	243782739	243783742	1003	DEL	AP10,AP11,AP13,AP6	AKT3	NM_181690	intron5-intron5
15	101453075	101453386	311	DEL	AP12,AP3,AP4,AP9	ALDH1A3/LOC10192775	NM_000693	intron12-intron12
17	19571909	19572320	411	DEL	AP1,AP4	ALDH3A2	NM_001369137	intron9-intron9
2	112623971	112624016	45	INS	AP1,AP12	ANAPC1	NM_022662	intron7-intron7
4	113985876	113986308	432	DEL	AP13,AP9	ANK2	NM_001354269	intron1-intron1
4	114064436	114064513	77	INS	AP10,AP11,AP2,AP3	ANK2	NM_001354269	intron1-intron1
4	114135101	114135586	485	DEL	AP12	ANK2	NM_001354269	intron4-intron4
12	133304136	133304346	210	INS	AP6	ANKLE2	NM_015114	intron12-intron12
16	89408459	89408554	95	INS	AP9	ANKRD11	NM_001256182	intron3-intron3

7	36441175	36441946	771	DEL	AP2	ANLN	NM_001284301	intron3-intron3
3	43565778	43565935	157	INS	AP10,AP11,AP12,AP13	ANO10	NM_018075	intron11-intron11
11	26526328	26526359	31	DEL	AP10,AP12,AP3,AP9	ANO3	NM_001313726	intron5-intron5
11	26601639	26601957	318	INS	AP4	ANO3	NM_001313726	intron15-intron15
11	26679948	26680053	105	DEL	AP10,AP11,AP3	ANO3	NM_001313726	intron27-intron27
11	22262800	22262928	128	INS	AP6	ANO5	NM_001142649	intron9-intron9
11	22265278	22265450	172	DUP	AP13	ANO5	NM_001142649	intron9-intron9
4	80841014	80841057	43	DEL	AP1,AP10,AP12,AP3	ANTXR2	NM_001286781	intron16-intron16
4	80888046	80894103	6057	DEL	AP10,AP11,AP12,AP13,AP3,AP4,AP6,AP9	ANTXR2	NM_001286781	intron16-intron16
15	51222475	51222846	371	INS	AP9	AP4E1	NM_001252127	intron6-intron6
18	10462406	10462650	244	DEL	AP11	APCDD1	NM_153000	intron1-intron1
21	27252389	27252933	544	INS	AP11,AP13,AP4,AP9	APP	NM_001136016	exon17-txEnd
21	27374151	27374704	553	INV	AP10,AP11,AP12,AP13,AP3,AP4,AP6,AP9	APP	NM_001136016	intron6-intron6
6	157397677	157398023	346	INS	AP13	ARID1B	NM_001371656	intron6-intron6
22	51062326	51062358	32	DEL	AP10,AP11,AP12,AP13,AP4	ARSA	NM_000487	exon8-exon8
5	78253753	78254108	355	INS	AP13	ARSB	NM_000046	intron3-intron3
5	78276754	78277079	325	DEL	AP6	ARSB	NM_000046	intron1-intron1
5	78277729	78278053	324	DEL	AP13,AP3,AP9	ARSB	NM_000046	intron1-intron1
1	1465919	1466238	319	DEL	AP10	ATAD3A	NM_001170535	intron15-intron15
7	138420609	138420656	47	INS	AP10,AP11,AP3,AP4,AP6,AP9	ATP6V0A4	NM_130841	intron14-intron14
7	138468980	138469016	36	DEL	AP3	ATP6V0A4	NM_020632	intron2-intron2
7	138474596	138474778	182	INS	AP1,AP3	ATP6V0A4	NM_020632	intron1-intron1
13	52576728	52576883	155	INS	AP12,AP4	ATP7B	NM_000053	intron1-intron1
18	55380994	55381145	151	INS	AP12	ATP8B1	NM_001374385	intron2-intron2
X	76881497	76881559	62	DEL	AP1	ATRX	NM_000489	intron19-intron19
X	76906706	76906750	44	INS	AP10	ATRX	NM_000489	intron15-intron15
6	16367939	16367983	44	DEL	AP11	ATXN1	NM_001357857	intron7-intron7
14	92537352	92537402	50	INS	AP13,AP6	ATXN3	NM_001127696	intron8-exon9
13	31892037	31892186	149	INS	AP10,AP9	B3GLCT	NM_194318	intron13-intron13
15	73018135	73018218	83	DEL	AP6	BBS4	NM_001252678	intron7-intron7
7	33195321	33195693	372	INS	AP11	BBS9	NM_001348036	intron4-intron4
7	33348898	33348997	99	DEL	AP10	BBS9	NM_001348036	intron9-intron9
7	33403540	33403870	330	DEL	AP10,AP3,AP6	BBS9	NM_001348036	intron16-intron16
7	33576296	33576359	63	INS	AP3,AP4	BBS9	NM_001348036	intron21-intron21
18	60885835	60886060	225	DUP	AP10,AP3	BCL2	NM_000633	intron2-intron2
18	60932137	60932247	110	INS	AP10,AP11,AP12,AP13,AP3,AP4,AP9	BCL2	NM_000633	intron2-intron2
3	133133568	133133649	81	INS	AP12	BFSP2	NM_003571	intron1-intron1
7	34087397	34087538	141	INS	AP10,AP12,AP6,AP9	BMPER	NM_133468	intron8-intron8
10	88666182	88666526	344	DEL	AP10,AP11,AP12,AP13,AP3,AP6,AP9	BMPR1A	NM_004329	intron7-intron7
4	95870452	95870771	319	DEL	AP12,AP2	BMPR1B	NM_001203	intron2-intron2
2	203409935	203410074	139	INS	AP9	BMPR2	NM_001204	intron10-intron10
9	16682304	16682635	331	INS	AP13	BNC2	NM_001317939	intron2-intron2
9	16847393	16847519	126	DEL	AP9	BNC2	NM_001317939	intron1-intron1

7	134349999	134350428	429	DEL	AP1,AP11,AP12,AP13,AP6,AP9	BPGM	NM_001293085	intron3-intron3
13	32960701	32960733	32	DEL	AP6	BRCA2	NM_000059	intron24-intron24
6	105567842	105568182	340	DEL	AP1,AP10,AP11,AP12,AP2,AP4	BVES	NM_147147	intron5-intron5
11	73777311	73777371	60	INS	AP1,AP10,AP12,AP3,AP6,AP9	C2CD3	NM_001286577	intron24-intron24
5	40953955	40954031	76	INS	AP10,AP13,AP3,AP9	C7	NM_000587	intron9-intron9
1	57394750	57395002	252	INS	AP11,AP9	C8B	NM_000066	exon12-txEnd
12	2364956	2365257	301	DUP	AP10,AP11,AP13,AP3,AP6	CACNA1C	NM_000719	intron3-intron3
12	2467426	2467461	35	DEL	AP6	CACNA1C	NM_000719	intron3-intron3
12	2648366	2649066	700	DUP	AP10,AP11,AP12,AP13,AP6	CACNA1C	NM_000719	intron9-intron9
17	48665718	48665833	115	INS	AP11,AP12,AP9	CACNA1G	NM_001256324	intron9-intron9
10	18503075	18503589	514	DEL	AP11,AP9	CACNB2	NM_001167945	intron2-intron2
10	18731845	18731937	92	DEL	AP12	CACNB2	NM_001167945	intron3-intron3
2	152772499	152772542	43	DEL	AP4	CACNB4	NM_001330118	intron2-intron2
1	7044128	7044330	202	INS	AP13	CAMTA1	NM_001349608	intron2-intron2
1	7517099	7517132	33	DEL	AP6	CAMTA1	NM_001349608	intron4-intron4
17	77005260	77005315	55	DEL	AP3	CANT1	NM_001159772	intron1-intron1
6	17472276	17472595	319	DEL	AP1,AP3,AP4,AP6	CAP2	NM_001363533	intron4-intron4
13	111331380	111331414	34	INS	AP10	CARS2	NM_001352252	intron7-intron7
2	202146650	202149448	2798	DEL	AP11,AP12,AP3,AP4,AP6,AP9	CASP8	NM_001228	intron8-intron8
11	119124250	119124521	271	INS	AP4	CBL	NM_005188	intron2-intron2
18	57258186	57258527	341	DEL	AP10,AP11,AP13,AP2,AP4,AP9	CCBE1	NM_133459	intron2-intron2
14	91759356	91759922	566	DUP	AP9	CCDC88C	NM_001080414	intron23-intron23
3	45931754	45931796	42	INS	AP3	CCR9/LZTFL1	NM_001276378	intron2-intron2
11	833797	833877	80	INS	AP4	CD151	NM_004357	intron1-intron1
9	123257030	123259736	2706	DEL	AP12,AP3	CDK5RAP2	NM_001272039	intron12-intron12
4	85520548	85520584	36	DEL	AP10,AP11,AP13,AP3,AP9	CDS1	NM_001263	intron1-intron1
12	88520870	88520946	76	INS	AP1,AP10,AP12,AP2,AP3,AP4,AP6,AP9	CEP290	NM_025114	intron11-intron11
15	101051738	101052181	443	DEL	AP9	CERS3	NM_001290342	intron3-intron3
7	117252063	117252105	42	DEL	AP3	CFTR	NM_000492	intron20-intron20
15	93515792	93516110	318	DEL	AP10,AP11,AP12,AP3,AP6,AP9	CHD2	NM_001271	intron19-intron19
7	29303576	29303619	43	DEL	AP12	CHN2	NM_001293069	intron2-intron2
7	29399723	29399761	38	DEL	AP10	CHN2	NM_001293069	intron3-intron3
X	109948662	109948810	148	INS	AP12,AP3,AP6	CHRDL1	NM_001143981	intron6-intron6
12	120201091	120201208	117	INS	AP3,AP6,AP9	CIT	NM_001206999	intron19-intron19
X	49821980	49822355	375	DUP	AP10,AP12,AP13,AP4,AP6	CLCN5	NM_001127898	intron4-intron4
1	16376513	16376601	88	INS	AP12,AP13,AP4,AP9	CLCNKB	NM_000085	intron10-intron10
3	190119029	190119097	68	DEL	AP13,AP2,AP6,AP9	CLDN16	NM_006580	intron1-intron1
8	87595254	87595291	37	INS	AP11,AP13,AP2,AP4	CNGB3	NM_019098	intron15-intron15
7	146883956	146884169	213	DEL	AP13	CNTNAP2	NM_014141	intron7-intron7
7	147044513	147044572	59	DEL	AP12	CNTNAP2	NM_014141	intron8-intron8
7	147283612	147283660	48	INS	AP10	CNTNAP2	NM_014141	intron11-intron11
7	148072862	148076323	3461	DEL	AP11,AP6	CNTNAP2	NM_014141	intron20-intron20
7	148106880	148107021	141	DEL	AP12,AP3	CNTNAP2	NM_014141	intron22-intron22

16	70553015	70553335	320	DEL	AP12	COG4	NM_001195139	intron2-intron2
7	107059159	107063047	3888	INV	AP12,AP9	COG5	NM_006348	intron6-intron6
10	71610168	71610493	325	INS	AP9	COL13A1	NM_001130103	intron3-intron3
10	105817205	105817407	202	INS	AP1,AP3,AP9	COL17A1	NM_000494	intron16-intron16
21	46825209	46825262	53	INS	AP10	COL18A1	NM_130445	intron1-intron1
21	46926665	46926716	51	DEL	AP11,AP12	COL18A1	NM_130445	intron38-intron38
21	46927370	46927416	46	DEL	AP11,AP12	COL18A1	NM_130445	intron38-intron38
7	94051083	94051122	39	DEL	AP12,AP13	COL1A2	NM_000089	intron38-intron38
4	109922542	109922646	104	INS	AP3	COL25A1	NM_198721	intron5-intron5
9	116937319	116937372	53	INS	AP1,AP12,AP3,AP6	COL27A1	NM_032888	intron3-intron3
2	189845085	189845145	60	DEL	AP12,AP13,AP3,AP6	COL3A1	NM_000090	intron1-intron1
13	110812500	110812816	316	DEL	AP2	COL4A1	NM_001845	intron49-intron49
13	111076800	111077141	341	INS	AP10,AP13,AP4	COL4A2	NM_001846	intron4-exon5
9	137690415	137690499	84	DEL	AP11,AP12,AP6	COL5A1	NM_000093	intron37-intron37
9	137720489	137720588	99	INS	AP3	COL5A1/LOC101448202	NM_000093	intron63-intron63
21	47410371	47410437	66	INS	AP10	COL6A1	NM_001848	intron13-intron13
21	47540037	47540098	61	INS	AP2	COL6A2	NM_058174	intron15-intron15
2	238252156	238252194	38	DEL	AP2,AP3	COL6A3	NM_057167	intron35-intron35
4	783681	783834	153	DEL	AP13	CPLX1	NM_006651	intron3-intron3
1	197323945	197323986	41	DEL	AP10	CRB1	NM_001257965	intron6-intron6
11	46319021	46319058	37	DEL	AP11	CREB3L1	NM_052854	intron1-intron1
16	3918271	3918499	228	DEL	AP10,AP11	CREBBP	NM_001079846	intron1-intron1
19	18800290	18800801	511	INS	AP9	CRTC1	NM_001098482	intron1-intron1
22	26997611	26997946	335	INS	AP11	CRYBB1	NM_001887	exon5-intron5
2	209008236	209008276	40	DEL	AP3	CRYGB/LOC100507443	NM_005210	intron2-intron2
8	19412052	19412094	42	INS	AP1,AP10,AP13	CSGALNACT1	NM_001130518	intron3-intron3
18	77471990	77472029	39	INS	AP10	CTDP1	NM_004715	intron6-intron6
18	77499783	77500111	328	DEL	AP11	CTDP1	NM_004715	intron12-intron12
5	138111845	138112038	193	DEL	AP4	CTNNA1	NM_001290307	intron1-intron1
10	67831137	67831189	52	INS	AP12,AP13,AP2,AP3	CTNNA3	NM_001127384	intron14-intron14
10	68049101	68049358	257	INS	AP1,AP10,AP12	CTNNA3	NM_001127384	intron12-intron12
10	68105980	68106018	38	DEL	AP10	CTNNA3	NM_001127384	intron12-intron12
10	68127616	68127651	35	DEL	AP4	CTNNA3	NM_001127384	intron12-intron12
10	68283944	68283988	44	INS	AP10,AP11,AP6,AP9	CTNNA3	NM_001127384	intron10-intron10
10	68326810	68326876	66	INS	AP11,AP6	CTNNA3	NM_001127384	intron10-intron10
10	69061723	69061823	100	DEL	AP10,AP2	CTNNA3	NM_001127384	intron5-intron5
16	88779319	88779697	378	DUP	AP10,AP13	CTU2	NM_001012759	intron7-intron7
5	64286061	64286400	339	DEL	AP10,AP6	CWC27	NM_001297644	intron12-intron12
10	104590841	104590877	36	DEL	AP10	CYP17A1	NM_000102	intron7-intron7
20	52779058	52779162	104	INS	AP6	CYP24A1	NM_000782	intron7-intron7
4	108865988	108866032	44	INS	AP1,AP6,AP9	CYP2U1	NM_183075	intron1-intron1
1	58071859	58072086	227	INS	AP10,AP9	DAB1	NM_021080	intron3-intron3
1	58343211	58343540	329	DEL	AP10,AP3,AP4,AP6,AP9	DAB1	NM_021080	intron2-intron2

1	58416995	58417046	51	INS	AP10	DAB1	NM_021080	intron1-intron1
1	100703693	100703741	48	DEL	AP11,AP3,AP4,AP6	DBT	NM_001918	intron2-intron2
18	50462564	50463030	466	DEL	AP6,AP9	DCC	NM_005215	intron5-intron5
18	50732492	50732582	90	INS	AP10,AP2,AP9	DCC	NM_005215	intron10-intron10
18	50925982	50926311	329	DEL	AP1,AP11,AP13,AP2,AP3,AP6,AP9	DCC	NM_005215	intron18-intron18
18	50930750	50930811	61	DEL	AP11,AP3	DCC	NM_005215	intron19-intron19
6	24325365	24327811	2446	DEL	AP12	DCDC2	NM_016356	intron2-intron2
9	32463882	32464063	181	INS	AP10,AP3,AP9	DDX58	NM_014314	intron16-intron16
9	32520816	32520853	37	DEL	AP6	DDX58	NM_014314	intron1-intron1
11	677323	677784	461	DEL	AP10,AP11	DEAF1	NM_001293634	intron7-intron7
11	696741	696884	143	INS	AP13	DEAF1/TMEM80	NM_001367390	intron1-intron1
1	55334342	55334394	52	DEL	AP2	DHCR24	NM_014762	intron5-intron5
12	125433332	125433417	85	DEL	AP11,AP12,AP4,AP9	DHX37	NM_032656	intron25-intron25
13	60611164	60611300	136	INS	AP10,AP12	DIAPH3/DIAPH3-AS1	NM_001042517	intron5-intron5
2	233111614	233111749	135	INS	AP10,AP11,AP12,AP13,AP2,AP3,AP4,AP9	DIS3L2	NM_152383	intron11-intron11
8	13364087	13364197	110	INS	AP4	DLC1	NM_182643	intron1-intron1
19	46273466	46273513	47	DEL	AP10	DM1-AS/DMPK	NM_001288765	exon13-exon13
X	31546269	31546356	87	DEL	AP13	DMD	NM_004021	intron12-intron12
X	31577472	31577787	315	DEL	AP13	DMD	NM_004021	intron12-intron12
X	32619776	32619807	31	DEL	AP12	DMD	NM_004006	intron12-intron12
X	32897127	32897166	39	INS	AP10	DMD	NM_004006	intron2-intron2
X	32968065	32968130	65	INS	AP12	DMD	NM_004006	intron2-intron2
X	32987322	32989078	1756	DEL	AP6	DMD	NM_004006	intron2-intron2
X	33208588	33208914	326	DEL	AP6,AP9	DMD	NM_004006	intron1-intron1
X	33298072	33298170	98	INS	AP10	DMD	NM_000109	intron1-intron1
X	33302848	33303180	332	INS	AP13	DMD	NM_000109	intron1-intron1
15	51897686	51898050	364	DEL	AP10,AP11,AP9	DMXL2	NM_001174116	intron1-intron1
19	55671145	55671181	36	DEL	AP3	DNAAF3	NM_001256714	intron10-intron10
7	778688	778827	139	DEL	AP13	DNAAF5	NM_017802	intron2-intron2
7	824878	824955	77	DEL	AP11	DNAAF5	NM_017802	intron12-intron12
7	21587709	21587830	121	DEL	AP10,AP13	DNAH11	NM_001277115	intron2-intron2
17	76461582	76461623	41	DEL	AP9	DNAH17	NM_173628	intron56-intron56
17	76505129	76505311	182	DEL	AP3	DNAH17	NM_173628	intron27-intron27
5	13827593	13828226	633	INS	AP9	DNAH5	NM_001369	intron38-intron38
5	13855311	13855627	316	DEL	AP3	DNAH5	NM_001369	intron30-intron30
5	13895085	13895384	299	DEL	AP13,AP3,AP4,AP6,AP9	DNAH5	NM_001369	intron15-intron15
17	11557383	11557703	320	DEL	AP6	DNAH9	NM_001372	intron14-intron14
17	11597929	11598067	138	DEL	AP12	DNAH9	NM_001372	intron22-intron22
7	157149904	157149989	85	INS	AP9	DNAJB6	NM_005494	intron1-intron1
10	69585408	69585508	100	INS	AP10,AP11,AP12,AP13,AP3,AP4,AP6	DNAJC12	NM_021800	intron1-intron1
1	63151822	63152167	345	DEL	AP1,AP10,AP11,AP2,AP3,AP4,AP9	DOCK7	NM_001271999	intron1-intron1
9	403897	403984	87	DEL	AP11	DOCK8	NM_203447	intron26-intron26
4	3477941	3477992	51	DEL	AP9	DOK7	NM_001164673	intron3-intron3

4	3484820	3484906	86	DEL	AP12	DOK7	NM_001164673	intron4-intron4
21	34949911	34949971	60	DEL	AP3,AP9	DONSON	NM_017613	exon10-exon10
7	153529762	153529833	71	DEL	AP10	DPP6	NM_001364497	intron2-intron2
7	154113834	154113928	94	INS	AP10,AP12	DPP6	NM_001364497	intron2-intron2
7	154153814	154155079	1265	DEL	AP2,AP3,AP6,AP9	DPP6	NM_001364497	intron3-intron3
7	154382272	154382628	356	INS	AP6	DPP6	NM_001364497	intron6-intron6
7	154446112	154446174	62	INS	AP2,AP3,AP4,AP9	DPP6	NM_001364497	intron7-intron7
7	154586520	154586579	59	INS	AP10,AP11,AP2,AP6,AP9	DPP6	NM_001364497	intron12-intron12
7	154609468	154609555	87	DEL	AP13	DPP6	NM_001364497	intron17-intron17
1	97599563	97599600	37	DEL	AP13	DPYD/DPYD-AS1	NM_000110	intron20-intron20
6	116642460	116642508	48	INS	AP1	DSE	NM_001374522	intron2-intron2
6	116750822	116751142	320	DEL	AP4,AP9	DSE	NM_001374522	intron4-intron4
6	56758354	56760959	2605	DEL	AP12,AP6	DST	NM_001144769	intron3-intron3
18	46593458	46593902	444	DUP	AP12,AP4	DYM	NM_001353210	intron16-intron16
18	46816093	46816160	67	DEL	AP12	DYM	NM_001353210	intron8-intron8
11	103087929	103088089	160	DEL	AP11,AP12,AP13,AP2,AP3,AP4,AP6,AP9	DYNC2H1	NM_001080463	intron55-intron55
2	71894430	71894462	32	DEL	AP4	DYSF	NM_001130976	intron45-intron45
10	135182853	135182961	108	DEL	AP10,AP9	ECHS1	NM_004092	intron3-intron3
X	68865427	68865506	79	INS	AP10,AP12,AP13,AP3,AP6	EDA	NM_001005610	intron1-intron1
9	140563768	140564032	264	DUP	AP11,AP4	EHMT1	NM_001354611	intron1-intron1
9	140610465	140611020	555	DUP	AP6	EHMT1	NM_001354611	intron2-intron2
1	45408062	45408174	112	INS	AP12	EIF2B3	NM_020365	intron3-intron3
1	45417621	45417934	313	DEL	AP12	EIF2B3	NM_020365	intron3-intron3
6	53171004	53171062	58	DEL	AP1	ELOVL5	NM_001242828	intron1-intron1
1	29227718	29227878	160	DEL	AP10	EPB41	NM_001166005	intron1-intron1
2	212551246	212551550	304	DEL	AP1,AP11,AP12,AP4,AP6,AP9	ERBB4	NM_001042599	intron12-intron12
2	212700043	212700116	73	DEL	AP10	ERBB4	NM_001042599	intron3-intron3
2	212771238	212775156	3918	DEL	AP4	ERBB4	NM_001042599	intron3-intron3
2	213195781	213195886	105	INS	AP11,AP4,AP6,AP9	ERBB4	NM_001042599	intron1-intron1
2	213311309	213311345	36	DEL	AP3	ERBB4	NM_001042599	intron1-intron1
8	27662507	27662840	333	DEL	AP13,AP2	ESCO2	NM_001017420	exon11-txEnd
15	76522853	76522942	89	INS	AP10,AP11,AP12,AP3,AP6,AP9	ETFA	NM_000126	intron10-intron10
12	12026411	12027153	742	DEL	AP11,AP13	ETV6	NM_001987	intron5-intron5
2	72478232	72478274	42	INS	AP3	EXOC6B	NM_001321729	intron20-intron20
2	72739452	72739496	44	DEL	AP3	EXOC6B	NM_001321729	intron11-intron11
8	118980764	118981117	353	DEL	AP10,AP6	EXT1	NM_000127	intron1-intron1
8	72214745	72217809	3064	DEL	AP1	EYA1	NM_172058	intron6-intron6
6	65152734	65152780	46	DEL	AP11,AP9	EYS	NM_001292009	intron26-intron26
6	65185156	65185474	318	DEL	AP11,AP12,AP2,AP9	EYS	NM_001292009	intron26-intron26
6	65342504	65342557	53	INS	AP1,AP10,AP11,AP12,AP3,AP4,AP9	EYS	NM_001292009	intron22-intron22
6	65414374	65414406	32	DEL	AP2	EYS	NM_001292009	intron22-intron22
6	65768319	65768774	455	DEL	AP11,AP6	EYS	NM_001292009	intron12-intron12
6	65988220	65988285	65	INS	AP10,AP4	EYS	NM_001292009	intron12-intron12

6	66163974	66164302	328	INS	AP4	EYS	NM_001292009	intron5-intron5
6	66260027	66262028	2001	DEL	AP10,AP11,AP12,AP13,AP3,AP4,AP6,AP9	EYS	NM_001292009	intron2-intron2
6	66274841	66275173	332	DEL	AP10,AP12,AP3,AP4,AP9	EYS	NM_001292009	intron2-intron2
6	66399045	66404687	5642	DEL	AP10,AP6	EYS	NM_001292009	intron1-intron1
7	148563591	148563644	53	DEL	AP11	EZH2	NM_001203249	intron1-intron1
13	113790940	113791272	332	DEL	AP11,AP2,AP9	F10	NM_000504	intron2-intron2
6	6277349	6277615	266	DEL	AP10,AP12,AP6,AP9	F13A1	NM_000129	intron3-intron3
1	197011864	197012170	306	INS	AP10,AP4,AP6	F13B	NM_001994	intron10-intron10
15	31221929	31222010	81	DEL	AP9	FAN1	NM_014967	intron12-intron12
6	5348761	5349096	335	INS	AP3	FARS2	NM_001318872	intron1-intron1
6	5738544	5738582	38	DEL	AP4	FARS2	NM_001318872	intron6-intron6
5	150914537	150914576	39	INS	AP3	FAT2	NM_001447	intron11-intron11
22	45964509	45964705	196	DEL	AP11	FBLN1	NM_006486	intron14-intron14
9	97387410	97387736	326	INS	AP3	FBP1	NM_000507	intron1-intron1
3	192045373	192045705	332	INS	AP11	FGF12	NM_001377294	intron3-intron3
3	192063187	192063501	314	DEL	AP11,AP12,AP3,AP6	FGF12	NM_001377294	intron2-intron2
3	192358911	192359211	300	DEL	AP11,AP9	FGF12	NM_001377292	intron2-intron2
3	192429056	192429102	46	DEL	AP2	FGF12	NM_001377292	intron2-intron2
13	102455914	102455950	36	DEL	AP6	FGF14	NM_001321938	intron5-intron5
13	102813914	102814038	124	DEL	AP1,AP10,AP11,AP2,AP3,AP6	FGF14	NM_001321938	intron3-intron3
13	102886533	102886726	193	DEL	AP12,AP3	FGF14	NM_001321938	intron2-intron2
13	103026996	103027033	37	DEL	AP4	FGF14/FGF14-IT1	NM_001321938	intron2-intron2
3	187898593	187898733	140	INS	AP12	FLJ42393/LPP	NM_001375455	intron1-intron1
1	213039223	213039401	178	DEL	AP10,AP9	FLVCR1	NM_014053	intron2-intron2
1	240302567	240302662	95	DEL	AP12	FMN2	NM_001305424	intron2-intron2
1	240348222	240348519	297	DUP	AP11	FMN2	NM_001305424	intron4-intron4
1	240617000	240617325	325	DEL	AP13,AP2,AP3,AP9	FMN2	NM_001305424	intron17-intron17
3	71242345	71242654	309	DEL	AP10,AP4	FOXP1	NM_001244814	intron2-intron2
3	71376465	71376714	249	INS	AP11,AP12	FOXP1	NM_001244808	intron4-intron4
7	113953505	113953819	314	DEL	AP3,AP4	FOXP2	NR_033766	intron2-intron2
7	114227927	114228002	75	INS	AP13,AP3	FOXP2	NR_033766	intron6-intron6
1	74755166	74755291	125	DEL	AP1,AP10,AP11,AP12,AP13,AP2,AP3,AP4,AP6,AP9	FPGT-TNNI3K/TNNI3K	NM_015978	intron5-intron5
4	79159510	79159847	337	INS	AP4	FRAS1	NM_025074	intron3-intron3
4	79269120	79275203	6083	DEL	AP2,AP3,AP4,AP9	FRAS1	NM_025074	intron20-intron20
13	39379287	39383419	4132	DEL	AP11	FREM2	NM_207361	intron6-intron6
X	11953190	11959442	6252	DEL	AP10,AP12	FRMPD4	NM_001368395	intron3-intron3
2	49354879	49354959	80	DEL	AP10,AP2,AP3,AP9	FSHR	NM_000145	intron1-intron1
16	53823784	53823821	37	DEL	AP3,AP4	FTO	NM_001363891	intron1-intron1
14	66077925	66077980	55	DEL	AP10	FUT8	NM_004480	intron2-intron2
9	71665329	71665568	239	DEL	AP10,AP11,AP2,AP4,AP9	FXN	NM_000144	intron2-intron2
3	46018844	46018892	48	DEL	AP12,AP4,AP9	FYCO1	NM_024513	intron4-intron4
9	101209338	101209651	313	DEL	AP3	GABBR2	NM_005458	intron7-intron7
9	101309043	101311664	2621	DEL	AP13	GABBR2	NM_005458	intron2-intron2

15	27137546	27137600	54	DEL	AP10	GABRA5	NM_000810	intron6-intron6
4	47248362	47248405	43	DEL	AP3	GABRB1	NM_000812	intron4-intron4
15	27009296	27009344	48	DEL	AP9	GABRB3	NM_001278631	intron3-intron3
2	38913368	38913419	51	INS	AP11	GALM	NM_138801	intron3-intron3
2	166623167	166623301	134	INS	AP3	GALNT3	NM_004482	intron2-intron2
8	75375341	75375695	354	INS	AP10,AP11,AP3,AP6,AP9	GDAP1	NM_001362931	intron5-intron5
3	33085270	33085353	83	INS	AP11	GLB1	NM_001079811	intron10-intron10
9	6629933	6629981	48	INS	AP3	GLDC	NM_000170	intron2-intron2
2	121680864	121680900	36	DEL	AP9	GLI2	NM_001371271	intron2-intron2
9	3953761	3953800	39	DEL	AP9	GLIS3	NM_001042413	intron4-intron4
18	11874561	11875039	478	INS	AP11,AP12,AP3	GNAL	NM_182978	intron10-intron10
12	102141413	102142069	656	INS	AP13	GNPTAB	NM_024312	intron20-intron20
12	65134833	65135013	180	DEL	AP1,AP10,AP12,AP13,AP3,AP6	GNS	NM_002076	intron6-intron6
13	93883564	93883670	106	DEL	AP10,AP13,AP4	GPC6	NM_005708	intron1-intron1
13	94105809	94106150	341	INS	AP11,AP12	GPC6	NM_005708	intron1-intron1
13	94163216	94163339	123	INS	AP4	GPC6	NM_005708	intron1-intron1
13	94392426	94392525	99	DEL	AP11,AP13	GPC6	NM_005708	intron2-intron2
13	95044888	95045033	145	DEL	AP13,AP2	GPC6	NM_005708	intron7-intron7
3	32153718	32153878	160	DEL	AP11,AP3,AP6	GPD1L	NM_015141	intron1-intron1
3	32172207	32172249	42	DEL	AP13	GPD1L	NM_015141	intron2-intron2
3	32201771	32202103	332	DEL	AP9	GPD1L	NM_015141	intron7-intron7
3	32203870	32204154	284	DEL	AP11,AP3	GPD1L	NM_015141	intron7-intron7
14	67012205	67012305	100	DEL	AP3	GPHN	NM_001024218	intron1-intron1
14	67407796	67407855	59	DEL	AP11	GPHN	NM_001024218	intron7-intron7
1	240675802	240675985	183	INS	AP12,AP9	GREM2	NM_022469	intron1-intron1
1	240708772	240708810	38	DEL	AP12	GREM2	NM_022469	intron1-intron1
11	105765515	105765834	319	DEL	AP11,AP13	GRIA4	NM_001077244	intron6-intron6
4	93499199	93499255	56	DEL	AP10,AP12	GRID2	NM_001510	intron1-intron1
4	93567358	93570163	2805	DUP	AP12,AP6	GRID2	NM_001510	intron2-intron2
4	94165633	94165777	144	DEL	AP1,AP3	GRID2	NM_001510	intron8-intron8
4	94662314	94662352	38	DEL	AP10,AP12,AP3,AP6	GRID2	NM_001510	intron14-intron14
6	102491666	102491710	44	INS	AP2	GRIK2	NM_001166247	intron14-intron14
12	13941602	13941706	104	DEL	AP1,AP11,AP12,AP13,AP3,AP4,AP6	GRIN2B	NM_000834	intron3-intron3
12	67116985	67117061	76	DEL	AP10,AP9	GRIP1	NM_001366723	intron1-intron1
4	42905161	42905849	688	DEL	AP11,AP9	GRXCR1	NM_001080476	intron1-intron1
4	42922732	42923077	345	INS	AP12	GRXCR1	NM_001080476	intron1-intron1
20	33537824	33537873	49	INS	AP4	GSS	NM_000178	intron2-intron2
19	49495744	49495780	36	INS	AP12	GYS1	NM_001161587	intron1-intron1
12	21737302	21737336	34	DEL	AP12	GYS2	NM_021957	intron1-intron1
6	105260826	105263759	2933	DEL	AP10,AP13	HACE1	NM_001321083	intron6-intron6
15	28430088	28430347	259	INS	AP11,AP13,AP3,AP4,AP6,AP9	HERC2	NM_004667	intron56-intron56
15	72639532	72639885	353	INS	AP10,AP11,AP4	HEXA	NM_000520	intron10-intron10
5	73952303	73952348	45	DEL	AP12,AP9	HEXB	NM_001292004	intron1-intron1

7	81358335	81358611	276	DEL	AP1,AP11,AP12,AP13,AP3,AP4,AP9	HGF	NM_000601	intron8-intron8
6	143161427	143161511	84	DEL	AP10,AP11,AP12,AP13,AP2,AP3,AP4,AP6	HIVEP2	NM_006734	intron1-intron1
12	122289586	122289940	354	DEL	AP10,AP3,AP4,AP9	HPD	NM_002150	intron7-intron7
10	100496979	100497287	308	DEL	AP10,AP4	HPSE2	NM_021828	intron4-intron4
5	118853598	118853648	50	DEL	AP6	HSD17B4	NM_000414	intron17-intron17
20	2639732	2639788	56	INS	AP6	IDH3B	NM_001330763	intron11-intron11
3	129184476	129184785	309	DEL	AP12	IFT122	NM_001280545	intron8-intron8
6	160521754	160521811	57	DEL	AP10,AP11,AP13,AP3	IGF2R	NM_000876	intron45-intron45
8	42185315	42185635	320	DEL	AP1,AP10,AP11,AP12,AP13,AP3,AP4	IKBKB	NM_001242778	intron19-intron19
X	29337269	29337312	43	INS	AP3	IL1RAPL1	NM_014271	intron3-intron3
X	29353370	29353412	42	DEL	AP13	IL1RAPL1	NM_014271	intron3-intron3
X	29525484	29525672	188	INS	AP6	IL1RAPL1	NM_014271	intron5-intron5
X	29821294	29821440	146	DEL	AP12	IL1RAPL1	NM_014271	intron6-intron6
10	6064768	6064842	74	DEL	AP6,AP9	IL2RA	NM_000417	intron3-intron3
10	6073407	6073458	51	DEL	AP9	IL2RA	NM_000417	intron1-intron1
10	6097377	6097875	498	DEL	AP11,AP6	IL2RA	NM_000417	intron1-intron1
6	76746502	76746583	81	DEL	AP12	IMPG1	NM_001563	intron2-intron2
3	100945069	100945110	41	DEL	AP2,AP4,AP6	IMPG2	NM_016247	exon19-exon19
15	40714966	40715155	189	INS	AP13	IVD	NM_001354598	intron11-intron11
11	133964510	133964571	61	DEL	AP2	JAM3	NM_001205329	intron1-intron1
16	87705204	87705260	56	DEL	AP3,AP4,AP6	JPH3	NR_073379	intron2-intron2
16	87726167	87726210	43	DEL	AP4	JPH3	NR_073379	intron4-intron4
9	707446	708017	571	DEL	AP12	KANK1	NM_001256876	intron6-intron6
1	210891707	210891858	151	INS	AP12,AP3,AP4	KCNH1	NM_002238	intron10-intron10
21	39004547	39004897	350	INS	AP6	KCNJ6	NM_002240	intron3-intron3
21	39050072	39050155	83	INS	AP1,AP10,AP3,AP9	KCNJ6	NM_002240	intron3-intron3
21	39076535	39076599	64	DEL	AP3	KCNJ6	NM_002240	intron3-intron3
21	39080012	39080499	487	INS	AP1,AP4	KCNJ6	NM_002240	intron3-intron3
21	39196245	39196338	93	INS	AP10,AP3,AP4,AP9	KCNJ6	NM_002240	intron2-intron2
21	39233186	39233234	48	DEL	AP12	KCNJ6	NM_002240	intron1-intron1
10	78800905	78801223	318	DEL	AP13	KCNMA1	NM_001322838	intron15-intron15
10	79325774	79325815	41	DEL	AP10	KCNMA1	NM_001014797	intron1-intron1
8	133315817	133315855	38	INS	AP12	KCNQ3	NM_001204824	intron1-intron1
6	73651391	73651454	63	DEL	AP11,AP3	KCNQ5	NM_001160130	intron1-intron1
9	138649895	138650028	133	DEL	AP11	KCNT1	NM_001272003	intron8-intron8
18	24045976	24046054	78	DEL	AP6	KCTD1	NM_001142730	intron3-intron3
18	24134023	24134436	413	DEL	AP3	KCTD1	NM_198991	intron2-intron2
16	27724963	27725201	238	INS	AP10,AP11,AP9	KIAA0556/LOC10012807	NM_015202	intron13-intron13
5	93916283	93916629	346	DEL	AP11,AP13	KIAA0825	NM_001145678	intron2-intron2
5	61635355	61635683	328	INS	AP10	KIF2A	NM_001243952	intron2-intron2
12	57951986	57952255	269	DEL	AP3	KIF5A	NM_001354705	intron1-intron1
4	55577813	55577889	76	DEL	AP3	KIT	NM_001093772	intron7-intron7
2	10184251	10184297	46	DEL	AP11,AP13,AP3,AP6	KLF11	NM_003597	intron1-intron1

5	137022568	137023896	1328	DEL	AP10,AP11,AP12,AP3,AP9	KLHL3	NM_001257195	intron3-intron3
11	67965907	67965942	35	DEL	AP11	KMT5B	NM_001369426	intron1-intron1
22	29515983	29516631	648	DUP	AP12	KREMEN1	NM_001039570	intron3-intron3
2	143800161	143800195	34	DEL	AP12	KYNU	NM_001199241	exon15-exon15
18	6952980	6953019	39	DEL	AP6	LAMA1	NM_005559	intron57-intron57
18	7073845	7073893	48	DEL	AP13	LAMA1	NM_005559	intron3-intron3
6	129737079	129737393	314	DEL	AP10,AP4	LAMA2	NM_000426	intron40-intron40
6	129828230	129828587	357	INS	AP12	LAMA2	NM_000426	intron61-intron61
18	21490977	21491121	144	DEL	AP6,AP9	LAMA3	NM_001127717	intron54-intron54
3	45542650	45542991	341	INS	AP12,AP13	LARS2/LARS2-AS1	NM_001368263	intron14-intron14
12	65588606	65588714	108	INS	AP3	LEMD3	NM_001167614	intron1-intron1
1	65909628	65909898	270	DEL	AP4	LEPR	NM_001003680	intron2-intron2
1	66055933	66056278	345	INS	AP10,AP9	LEPR	NM_001003680	intron5-intron5
1	66079150	66079427	277	DEL	AP10,AP11,AP13,AP6,AP9	LEPR	NM_001003680	intron14-intron14
10	95545472	95546471	999	DEL	AP11,AP12,AP13,AP4,AP6,AP9	LGI1	NM_001308275	intron4-intron4
5	38511006	38511064	58	DEL	AP4	LIFR	NM_001127671	intron6-intron6
13	108867771	108867866	95	INS	AP3	LIG4	NM_001330595	exon1-intron1
15	77910866	77911241	375	DEL	AP12,AP13,AP9	LINGO1	NM_001301199	intron3-intron3
15	77991613	77992117	504	DUP	AP11	LINGO1	NM_001301198	intron1-intron1
15	78008485	78012583	4098	DEL	AP4	LINGO1	NM_001301197	intron1-intron1
16	964496	964829	333	DUP	AP12	LMF1	NM_001352021	intron3-intron3
1	165317168	165317254	86	DEL	AP10	LMX1A	NM_001174069	intron3-intron3
12	106890951	106891290	339	INS	AP10,AP12	LOC100287944/POLR3B	NM_018082	intron25-intron25
2	166888719	166888872	153	INS	AP2,AP9	LOC102724058/SCN1A	NM_001165963	intron19-intron19
18	2997374	2997458	84	DEL	AP3,AP9	LPIN2	NM_014646	intron1-intron1
3	188304836	188305175	339	DEL	AP1,AP11,AP12,AP13,AP2,AP3,AP6,AP9	LPP	NM_001375455	intron5-intron5
3	188325158	188325201	43	DEL	AP12,AP13	LPP	NM_001375455	intron5-intron5
3	188473595	188473636	41	DEL	AP1,AP11,AP12,AP4,AP6	LPP	NM_001375455	intron7-intron7
4	151293907	151294243	336	INS	AP11	LRBA	NM_001364905	intron47-intron47
4	151560894	151560951	57	DEL	AP6	LRBA	NM_001364905	intron37-intron37
4	151875129	151875222	93	INS	AP10,AP3,AP4	LRBA	NM_001364905	intron2-intron2
10	78255572	78261022	5450	DEL	AP1,AP4	LRMDA	NM_001305581	intron6-intron6
11	68156628	68156681	53	INS	AP6,AP9	LRP5	NM_001291902	intron6-intron6
11	68183100	68183411	311	DEL	AP11,AP12	LRP5	NM_001291902	intron12-intron12
22	21343986	21344028	42	DEL	AP4,AP6,AP9	LZTR1	NM_006767	intron7-intron7
7	1885062	1885190	128	DEL	AP12	MAD1L1	NM_001304525	intron3-intron3
7	2042674	2042742	68	INS	AP3,AP9	MAD1L1	NM_001013836	intron13-intron13
7	2172022	2172073	51	DEL	AP3	MAD1L1	NM_001013836	intron11-intron11
7	2200746	2200845	99	INS	AP10,AP11,AP13	MAD1L1	NM_001013836	intron10-intron10
11	47321174	47321237	63	INS	AP4,AP6,AP9	MADD	NM_001376651	intron22-intron22
7	77699495	77699535	40	DEL	AP2	MAGI2	NM_001301128	intron20-intron20
7	77959643	77959965	322	DEL	AP9	MAGI2	NM_001301128	intron9-intron9
7	78606779	78606825	46	DEL	AP11,AP2,AP4	MAGI2	NM_001301128	intron2-intron2

7	78953934	78954005	71	DEL	AP10	MAGI2	NM_001301128	intron1-intron1
7	78997174	78997232	58	INS	AP6	MAGI2	NM_001301128	intron1-intron1
11	95914918	95914962	44	DEL	AP1,AP10,AP12,AP3,AP9	MAML2	NM_032427	intron1-intron1
11	96001921	96003392	1471	DEL	AP6	MAML2	NM_032427	intron1-intron1
6	91237578	91237904	326	DEL	AP10,AP13,AP2,AP3,AP9	MAP3K7	NM_003188	intron12-intron12
18	32661959	32662279	320	DEL	AP11,AP2,AP3	MAPRE2	NM_001143826	intron3-intron3
5	68724041	68724494	453	DEL	AP12,AP6	MARVELD2	NM_001038603	intron3-intron3
3	168898582	168898634	52	INS	AP11,AP12,AP2	MECOM	NM_001205194	intron1-intron1
3	168909483	168909534	51	DEL	AP12	MECOM	NM_001205194	intron1-intron1
12	116715140	116715266	126	INS	AP10,AP11,AP12,AP3,AP4	MED13L	NM_015335	exon1-exon1
5	88032018	88032354	336	DEL	AP10,AP12,AP2,AP3,AP4,AP6	MEF2C	NM_001364353	intron4-intron4
5	88043136	88043477	341	DEL	AP1,AP3,AP4,AP6	MEF2C	NM_001364353	intron4-intron4
22	42140478	42140805	327	DEL	AP10,AP12,AP6,AP9	MEI1	NM_152513	intron12-intron12
7	116366194	116366314	120	INS	AP11,AP2	MET	NM_001324401	intron2-intron2
3	70013473	70013887	414	DEL	AP11	MITF	NM_001354604	intron9-intron9
3	154843069	154843129	60	DEL	AP2	MME	NM_001354642	intron8-intron8
11	102471852	102472163	311	DEL	AP9	MMP20	NM_004771	intron6-intron6
21	33672989	33673073	84	INS	AP10	MRAP	NM_001285394	intron2-intron2
5	80039374	80039687	313	DEL	AP10,AP11,AP12,AP13,AP3,AP4,AP6,AP9	MSH3	NM_002439	intron11-intron11
8	15997255	15997314	59	DEL	AP12,AP9	MSR1	NM_138716	intron8-intron8
6	74216636	74217351	715	INS	AP10,AP3	MTO1	NM_001123226	exon13-exon13
9	113463932	113464153	221	DEL	AP1	MUSK	NM_001166280	intron5-intron5
12	102030823	102030908	85	INS	AP1,AP10,AP13,AP3,AP4,AP6	MYBPC1	NM_001254718	intron8-intron8
16	15869264	15869317	53	DEL	AP13,AP4	MYH11	NM_001040113	intron9-intron9
15	59431438	59431472	34	DEL	AP6	MYO1E	NM_004998	intron26-intron26
18	47694970	47698386	3416	DEL	AP12,AP13,AP4,AP9	MYO5B	NM_001080467	intron1-intron1
15	72217498	72217548	50	DEL	AP4,AP9	MYO9A	NM_006901	intron18-intron18
2	1826541	1826604	63	DEL	AP12,AP9	MYT1L	NM_001329845	intron21-intron21
2	1852059	1852109	50	DEL	AP12	MYT1L	NM_001329845	intron19-intron19
2	1865365	1865769	404	DUP	AP13,AP3,AP4,AP9	MYT1L	NM_001329845	intron18-intron18
2	2239127	2239202	75	DEL	AP2,AP3,AP4	MYT1L	NM_001329845	intron2-intron2
2	2266692	2266762	70	INS	AP11,AP3	MYT1L	NM_001329845	intron2-intron2
13	101894141	101896423	2282	DEL	AP6,AP9	NALCN	NM_001350748	intron11-intron11
2	15519545	15519865	320	INS	AP3	NBAS	NM_015909	exon30-intron30
2	240861056	240861096	40	DEL	AP10	NDUFA10	NR_136158	intron7-intron7
2	240865209	240867500	2291	DEL	AP13	NDUFA10	NR_136158	intron7-intron7
2	240958947	240959271	324	DEL	AP1,AP10,AP11,AP12,AP9	NDUFA10	NR_136158	intron3-intron3
8	96103236	96103276	40	DEL	AP1,AP13,AP4,AP9	NDUFAF6	NR_148913	intron8-intron8
5	1812644	1812970	326	INS	AP3	NDUFS6	NM_004553	intron2-intron2
2	152476862	152476895	33	DEL	AP6	NEB	NM_001271208	intron72-intron72
18	55801583	55801914	331	DEL	AP11,AP12,AP13,AP3,AP6	NEDD4L	NM_001144967	intron1-intron1
17	29662292	29662413	121	INS	AP1,AP11,AP4,AP6	NF1	NM_000267	intron39-intron39
9	14388366	14388407	41	DEL	AP12	NFIB	NM_001190738	intron1-intron1

9	14498762	14498820	58	DEL	AP11	NFIB	NM_001369458	intron1-intron1
3	25802351	25802392	41	DEL	AP11,AP12,AP13,AP2,AP3,AP6,AP9	NGLY1	NM_018297	intron3-intron3
3	25824975	25825027	52	DEL	AP1,AP11,AP13,AP3,AP9	NGLY1	NM_001145294	intron1-intron1
X	17502755	17502824	69	DEL	AP12,AP6	NHS	NM_001291867	intron1-intron1
X	17520624	17520941	317	DEL	AP12,AP6	NHS	NM_001291867	intron1-intron1
15	23066394	23066531	137	DEL	AP1,AP13	NIPA1	NM_144599	intron1-intron1
1	10028615	10028764	149	INS	AP10,AP11,AP13,AP9	NMNAT1	NM_001297778	intron1-intron1
12	117797678	117797725	47	DEL	AP3	NOS1	NM_000620	intron1-intron1
12	100907587	100907626	39	DEL	AP9	NR1H4	NM_001206977	intron5-intron5
4	149287576	149287636	60	DUP	AP9	NR3C2	NM_000901	intron2-intron2
2	50360414	50360453	39	INS	AP3,AP4	NRXN1	NM_001330078	intron18-intron18
2	50602633	50602976	343	INS	AP12,AP13,AP3	NRXN1	NM_001330078	intron17-intron17
8	126305707	126305910	203	DEL	AP11,AP4	NSMCE2	NM_001349485	intron4-intron4
8	126336807	126337128	321	DEL	AP10,AP2	NSMCE2	NM_001349485	intron4-intron4
5	6616779	6617028	249	INS	AP10,AP3,AP9	NSUN2	NM_001193455	intron7-intron8
1	156806527	156806585	58	DEL	AP13,AP9	NTRK1	NM_001007792	intron1-intron1
9	87345367	87345682	315	DEL	AP12	NTRK2	NM_001007097	intron11-intron11
17	73208322	73208355	33	DEL	AP12	NUP85	NM_001330472	intron4-intron4
16	56774119	56774158	39	DEL	AP12	NUP93	NM_014669	intron1-intron1
17	744615	744964	349	DUP	AP10,AP12	NXN	NM_022463	intron1-intron1
17	801626	802222	596	DEL	AP12	NXN	NM_022463	intron1-intron1
15	28175794	28176197	403	INS	AP10,AP11	OCA2	NM_000275	intron18-intron18
11	132558588	132558880	292	DEL	AP1,AP11,AP6	OPCML	NM_001319103	intron1-intron1
11	132995568	132995604	36	DEL	AP13	OPCML	NM_001012393	intron1-intron1
11	133123705	133123753	48	DEL	AP10,AP11	OPCML	NM_001012393	intron1-intron1
10	13159416	13159452	36	DEL	AP11,AP9	OPTN	NM_001008211	intron7-intron7
16	21718916	21718955	39	DEL	AP4	OTOA	NM_144672	intron12-intron12
12	80573892	80574059	167	DEL	AP1,AP4,AP9	OTOGL	NM_001368062	intron5-intron5
8	107417372	107417415	43	DEL	AP3,AP4,AP9	OXR1	NM_001198533	intron2-intron2
8	107434022	107434064	42	INS	AP11,AP2,AP3,AP4,AP9	OXR1	NM_001198533	intron2-intron2
8	107511099	107511428	329	DEL	AP12,AP13	OXR1	NM_001198533	intron2-intron2
8	107728562	107728893	331	DEL	AP10,AP2	OXR1	NM_001198533	intron11-intron11
8	107758527	107758572	45	INS	AP10,AP11,AP12,AP13,AP3,AP6	OXR1	NM_001198533	intron16-intron16
11	66008249	66008578	329	INS	AP3	PACS1	NM_018026	intron21-intron21
12	103261910	103261960	50	DEL	AP12,AP3	PAH	NM_000277	intron4-intron4
11	77043398	77043571	173	INS	AP9	PAK1	NM_001376269	intron14-intron14
11	93871028	93871128	100	INS	AP10,AP3	PANX1	NM_015368	intron1-intron1
1	164540827	164540858	31	DEL	AP9	PBX1	NM_002585	intron2-intron2
1	164845205	164845248	43	DEL	AP11	PBX1	NM_001353131	intron7-intron7
1	164845207	164845247	40	DEL	AP9	PBX1	NM_001353131	intron7-intron7
1	164845209	164845249	40	DEL	AP9	PBX1	NM_001353131	intron7-intron7
1	164845210	164845254	44	DEL	AP10,AP2,AP3	PBX1	NM_001353131	intron7-intron7
1	164845234	164845267	33	INS	AP13	PBX1	NM_001353131	intron7-intron7

1	164845236	164845269	33	INS	AP13	PBX1	NM_001353131	intron7-intron7
11	66711484	66713291	1807	DEL	AP11	PC	NM_000920	intron2-intron2
10	55615665	55615707	42	INS	AP9	PCDH15	NM_001142771	intron29-intron29
10	55648055	55648195	140	INS	AP12	PCDH15	NM_001142771	intron27-intron27
10	55725753	55725841	88	DEL	AP4	PCDH15	NM_001142771	intron22-intron22
10	55761803	55761837	34	DEL	AP1	PCDH15	NM_001142771	intron21-intron21
10	55893665	55894123	458	DEL	AP3,AP4,AP6,AP9	PCDH15	NM_001142771	intron15-intron15
10	55958254	55958546	292	DEL	AP10	PCDH15	NM_001142771	intron11-intron11
10	56521450	56521481	31	DEL	AP11	PCDH15	NM_001142771	intron1-intron1
10	56682778	56684358	1580	DEL	AP12	PCDH15	NM_001354404	intron3-intron3
10	56762356	56762424	68	INS	AP10,AP13,AP2,AP3,AP4,AP6,AP9	PCDH15	NM_001354404	intron3-intron3
10	56767188	56773196	6008	INV	AP1,AP11,AP12	PCDH15	NM_001354404	intron3-intron3
10	56902361	56902705	344	INS	AP12,AP13	PCDH15	NM_001354404	intron3-intron3
10	56919102	56919134	32	DEL	AP9	PCDH15	NM_001354404	intron3-intron3
10	57161852	57161890	38	DEL	AP6	PCDH15	NM_001354404	intron2-intron2
2	178676250	178676477	227	INS	AP6	PDE11A	NM_001077196	intron6-intron6
2	178810558	178810606	48	DEL	AP12	PDE11A	NM_016953	intron2-intron2
5	58274032	58274164	132	DEL	AP9	PDE4D	NM_001197223	intron6-intron6
5	58327202	58327238	36	DEL	AP6	PDE4D	NM_001197221	intron2-intron2
5	58869905	58869956	51	DEL	AP6	PDE4D	NM_001349242	intron1-intron1
17	79619831	79619886	55	DEL	AP10,AP11,AP12,AP13,AP3,AP4	PDE6G	NM_001365725	intron2-intron2
10	26999004	27002153	3149	DEL	AP11	PDSS1	NM_001321978	intron5-intron5
6	107723671	107723832	161	INS	AP12	PDSS2	NM_020381	intron1-intron1
7	92144388	92144420	32	DEL	AP3	PEX1	NM_000466	intron5-intron5
12	7348825	7348881	56	DEL	AP13	PEX5	NM_001351124	intron5-intron5
12	7355769	7356073	304	INS	AP13	PEX5	NM_001351124	intron8-exon9
6	13196491	13196527	36	DEL	AP1,AP13,AP4,AP6	PHACTR1	NM_001374582	intron8-intron8
6	13231402	13231642	240	DEL	AP10	PHACTR1	NM_001374582	intron11-intron11
11	46036239	46036594	355	DEL	AP1,AP12,AP2	PHF21A	NM_001352028	intron6-intron6
16	30763551	30764009	458	INS	AP11	PHKG2	NM_001172432	intron4-intron4
18	10757445	10757529	84	DEL	AP10,AP11,AP13,AP4,AP9	PIEZO2	NM_022068	intron25-intron25
18	10911130	10911467	337	INS	AP12,AP9	PIEZO2	NM_022068	intron3-intron4
18	10915930	10915978	48	DEL	AP1,AP11,AP9	PIEZO2	NM_022068	intron3-intron3
18	10944496	10944529	33	DEL	AP11	PIEZO2	NM_022068	intron3-intron3
18	59713725	59714056	331	INS	AP3	PIGN	NM_012327	intron29-intron29
18	59728658	59728771	113	DEL	AP11,AP12,AP3,AP4,AP9	PIGN	NM_012327	intron29-intron29
18	59834520	59834564	44	DEL	AP10,AP12,AP2,AP3,AP9	PIGN	NM_012327	intron1-intron1
18	59852899	59852954	55	INS	AP2,AP3,AP4,AP9	PIGN	NM_012327	intron1-intron1
11	17195845	17196160	315	DEL	AP1,AP10,AP3,AP4	PIK3C2A	NM_001321378	intron2-intron2
11	17203155	17203196	41	DEL	AP12	PIK3C2A	NM_001321378	intron2-intron2
11	17215411	17215607	196	INS	AP11,AP4	PIK3C2A	NM_001321378	intron2-intron2
17	6422506	6422671	165	DEL	AP6	PITPNM3	NM_001165966	intron2-intron2
6	51517350	51517684	334	DEL	AP1,AP10,AP11,AP12,AP13,AP2,AP3,AP4,AP6,AP9	PKHD1	NM_138694	intron61-intron61

6	51739564	51745615	6051	DEL	AP10,AP11,AP3,AP6,AP9	PKHD1	NM_138694	intron46-intron46
6	51916612	51916655	43	DEL	AP10,AP3	PKHD1	NM_138694	intron21-intron21
12	32963604	32963921	317	DEL	AP10,AP11,AP12,AP13,AP4	PKP2	NM_004572	intron10-intron10
20	9149569	9149624	55	DEL	AP11,AP13,AP2,AP9	PLCB4	NM_001377134	intron2-intron2
20	9389100	9389168	68	INS	AP10,AP12	PLCB4	NM_001377134	intron21-intron21
6	161149397	161149509	112	INS	AP4	PLG	NM_000301	intron10-intron10
2	55877990	55878128	138	DEL	AP6	PNPT1	NM_033109	intron18-intron18
3	12351955	12352265	310	DEL	AP6	PPARG	NM_001354666	intron1-intron1
5	146026781	146026827	46	INS	AP10,AP11,AP3,AP6,AP9	PPP2R2B	NR_073526	intron5-intron5
5	146192048	146192081	33	DEL	AP4	PPP2R2B	NR_073526	intron1-intron1
5	146396113	146396177	64	INS	AP2,AP6	PPP2R2B	NM_001271900	intron2-intron2
4	102085246	102085818	572	INS	AP10,AP13	PPP3CA	NM_000944	intron2-intron2
1	3028033	3028072	39	INS	AP9	PRDM16	NM_022114	intron1-intron1
1	3079425	3079904	479	DUP	AP11,AP6	PRDM16	NM_022114	intron1-intron1
4	121808932	121809251	319	DEL	AP13,AP6	PRDM5	NM_001300823	intron2-intron2
5	122431836	122432034	198	INS	AP11,AP13,AP4	PRDM6	NM_001136239	intron2-intron2
12	42871283	42871330	47	DEL	AP11,AP4	PRICKLE1	NM_001144881	intron1-intron1
14	30295370	30295550	180	DEL	AP1	PRKD1	NM_001348390	intron1-intron1
10	53313464	53313524	60	DEL	AP10	PRKG1	NM_001098512	intron3-intron3
10	53827672	53827991	319	DEL	AP6	PRKG1	NM_001098512	intron7-intron7
10	53828727	53828778	51	DEL	AP6	PRKG1	NM_001098512	intron7-intron7
6	161795618	161795800	182	INS	AP12,AP4,AP6	PRKN	NM_004562	intron10-intron10
6	161973419	161973479	60	DEL	AP12,AP3	PRKN	NM_004562	intron8-intron8
6	162316509	162316729	220	DEL	AP12	PRKN	NM_004562	intron6-intron6
6	162801507	162801541	34	DEL	AP4	PRKN	NM_004562	intron2-intron2
5	35074614	35074652	38	INS	AP3	PRLR	NM_001204316	intron4-intron4
5	35083885	35083936	51	DEL	AP3	PRLR	NM_001204316	intron4-intron4
4	15987507	15987553	46	INS	AP1,AP10,AP11,AP3,AP9	PROM1	NM_001145849	intron20-intron20
17	40726730	40726764	34	DEL	AP12	PSMC3IP	NM_001256014	intron2-intron2
10	89676313	89676345	32	DEL	AP6	PTEN	NM_000314	intron2-intron2
1	214610129	214610173	44	DEL	AP12	PTPN14	NM_005401	intron3-intron3
11	48120066	48120377	311	DEL	AP10,AP4	PTPRJ	NM_001098503	intron1-intron1
14	51408899	51409531	632	DEL	AP12,AP4	PYGL	NM_001163940	intron1-intron1
11	64526477	64526563	86	INS	AP10,AP11,AP12,AP4,AP9	PYGM	NM_001164716	intron1-intron1
3	25030883	25032243	1360	DEL	AP10,AP12,AP13,AP2,AP3,AP4,AP6	RARB	NM_001290216	intron2-intron2
3	25508990	25509033	43	DEL	AP3,AP9	RARB	NM_001290216	intron5-intron5
6	88285774	88286250	476	DEL	AP3	RARS2	NM_001318785	intron1-intron1
15	38835026	38835065	39	DEL	AP4	RASGRP1	NM_001128602	intron2-intron2
10	112480400	112480454	54	DEL	AP3	RBM20	NM_001134363	intron1-intron1
4	26290467	26295385	4918	DEL	AP3	RBPJ	NM_001374401	intron1-intron1
7	103463072	103463399	327	DEL	AP1,AP10,AP11,AP12,AP13,AP2,AP4,AP6,AP9	RELN	NM_173054	intron3-intron3
7	103470504	103470566	62	INS	AP10,AP6	RELN	NM_173054	intron3-intron3
7	103612297	103612333	36	DEL	AP13	RELN	NM_173054	intron1-intron1

1	8446629	8446963	334	INS	AP10,AP11	RERE	NM_001042682	intron2-intron2
1	8729018	8729101	83	DEL	AP10,AP4,AP6	RERE	NM_001042681	intron1-intron1
6	72863851	72873534	9683	DEL	AP3	RIMS1	NM_014989	intron4-intron4
3	149593684	149593795	111	INS	AP1,AP10,AP11,AP6,AP9	RNF13	NM_007282	intron5-intron5
4	1086775	1086826	51	DEL	AP12,AP3	RNF212	NM_001366918	intron3-intron3
3	77213923	77213975	52	INS	AP12	ROBO2	NM_001290039	intron2-intron2
15	61216539	61216889	350	INS	AP11	RORA	NM_134261	intron1-intron1
15	61346046	61346090	44	INS	AP4	RORA	NM_134261	intron1-intron1
15	60835369	60835400	31	DEL	AP4	RORA/RORA-AS1	NM_134262	intron1-intron1
16	53713454	53713556	102	DEL	AP1,AP4	RPGRIP1L	NM_001127897	intron6-intron6
2	89001138	89001220	82	INS	AP12,AP13,AP3,AP4,AP6	RPIA	NM_144563	intron3-intron3
2	89029234	89032289	3055	DEL	AP3,AP4	RPIA	NM_144563	intron4-intron4
11	14348314	14348634	320	DEL	AP12,AP13,AP4,AP9	RRAS2	NM_001177315	intron1-intron1
1	38077346	38077420	74	DEL	AP10,AP12,AP13,AP3,AP6,AP9	RSPO1	NM_001038633	intron8-exon9
16	57231835	57231881	46	DEL	AP1,AP11	RSPRY1	NM_001305182	intron1-intron1
16	57234574	57234622	48	INS	AP3	RSPRY1	NM_001305182	intron1-intron1
16	57252625	57252951	326	DEL	AP1,AP11,AP12	RSPRY1	NM_001305163	intron8-intron8
3	158116907	158117252	345	DEL	AP12,AP13,AP4,AP6,AP9	RSRC1	NM_001271834	intron5-intron5
3	158195587	158195627	40	INS	AP13	RSRC1	NM_001271834	intron6-intron6
3	158223547	158223903	356	INS	AP13	RSRC1	NM_001271834	intron6-intron6
1	237449855	237450067	212	INS	AP10,AP3,AP6,AP9	RYR2	NM_001035	intron2-intron2
1	237679021	237679178	157	INS	AP3,AP4	RYR2	NM_001035	intron24-intron24
8	119447049	119447361	312	DEL	AP6	SAMD12	NR_146234	intron3-intron3
8	119618005	119618046	41	DEL	AP11,AP6	SAMD12	NR_146234	intron1-intron1
2	200312455	200312679	224	DEL	AP13	SATB2	NM_001172517	intron3-intron3
11	10292740	10293846	1106	DEL	AP3	SBF2	NM_030962	intron1-intron1
15	76723499	76723592	93	INS	AP4	SCAPER	NM_001353009	intron27-intron27
15	76884596	76896941	12345	DEL	AP3	SCAPER	NM_001353009	intron23-intron23
15	51978918	51979041	123	INS	AP3	SCG3	NM_001165257	intron3-intron3
3	39011492	39011531	39	DEL	AP12	SCN11A	NM_001349253	intron2-intron2
2	166905653	166905728	75	DEL	AP2	SCN1A	NM_001165963	intron9-intron9
2	166014692	166016744	2052	DEL	AP10,AP4,AP6	SCN3A	NM_001081676	intron9-intron9
16	23344422	23344476	54	DEL	AP6	SCNN1B	NM_000336	intron1-intron1
1	243643592	243644217	625	DUP	AP3	SDCCAG8	NM_001350246	intron18-intron18
14	39563442	39563495	53	DEL	AP1	SEC23A	NM_006364	intron2-intron2
6	108266114	108266421	307	DEL	AP1,AP10,AP11,AP3,AP4,AP9	SEC63	NM_007214	intron1-intron1
6	108274959	108275056	97	DEL	AP1	SEC63	NM_007214	intron1-intron1
1	26134788	26134833	45	DEL	AP2,AP3	SELENON	NM_020451	intron4-intron4
6	158548272	158549079	807	DEL	AP1,AP11,AP2,AP3,AP4	SERAC1	NM_032861	intron10-intron10
18	42448667	42448712	45	DEL	AP10,AP4	SETBP1	NM_001130110	intron2-intron2
5	155777255	155777300	45	INS	AP12,AP6	SGCD	NM_000337	intron3-intron3
4	2810478	2810773	295	DEL	AP10	SH3BP2	NM_001122681	intron1-intron1
5	171795464	171795672	208	DEL	AP3	SH3PXD2B	NM_001308175	intron6-intron6

5	148385841	148385940	99	DEL	AP3	SH3TC2	NM_024577	intron16-intron16
5	148401439	148401598	159	DEL	AP11,AP2,AP3	SH3TC2	NM_024577	intron12-intron12
22	51154450	51154532	82	DEL	AP13	SHANK3	NM_001372044	intron21-intron21
X	50538040	50538088	48	DEL	AP12	SHROOM4	NM_020717	intron1-intron1
15	48550269	48550591	322	DEL	AP13,AP3,AP6	SLC12A1	NM_000338	intron16-intron16
15	48562946	48563157	211	INS	AP10,AP4	SLC12A1	NM_000338	intron19-intron19
X	73679069	73679217	148	INS	AP10,AP11,AP9	SLC16A2	NM_006517	intron1-intron1
1	169442962	169443308	346	INS	AP4	SLC19A2	NM_006996	intron2-intron2
5	36649429	36649465	36	DEL	AP10,AP12,AP13	SLC1A3	NM_001289939	intron2-intron2
14	92941046	92941091	45	DEL	AP1	SLC24A4	NM_153648	intron12-intron12
2	172664711	172664822	111	DEL	AP10	SLC25A12	NM_003705	intron13-intron13
7	95764104	95764144	40	INS	AP12	SLC25A13	NM_014251	intron14-intron14
1	108733315	108737228	3913	DEL	AP9	SLC25A24	NM_213651	txStart-intron1
3	66335921	66336865	944	INS	AP11	SLC25A26	NM_173471	intron6-intron6
4	9927873	9927914	41	DEL	AP9	SLC2A9	NM_020041	intron6-intron6
4	9952298	9952551	253	DEL	AP10,AP12,AP3,AP4	SLC2A9	NM_020041	intron5-intron5
4	9952515	9952550	35	INS	AP13	SLC2A9	NM_020041	intron5-intron5
4	9982897	9983287	390	INS	AP11	SLC2A9	NM_020041	intron4-intron4
5	33973937	33973977	40	DEL	AP12,AP6	SLC45A2	NM_016180	intron2-intron2
5	1206475	1206546	71	DEL	AP13,AP9	SLC6A19	NM_001003841	intron1-intron1
3	45831051	45831106	55	DEL	AP1,AP6	SLC6A20	NM_020208	intron1-intron1
5	492292	492352	60	DEL	AP4	SLC9A3	NM_001284351	intron1-intron1
5	497823	497956	133	DEL	AP10	SLC9A3	NM_001284351	intron1-intron1
5	505488	505568	80	DEL	AP6	SLC9A3	NM_001284351	intron1-intron1
18	2748478	2748515	37	DEL	AP1,AP12,AP2,AP3,AP4,AP6	SMCHD1	NM_015295	intron30-intron30
6	168905864	168905942	78	DEL	AP10,AP6	SMOC2	NM_001166412	intron1-intron1
6	169024302	169024334	32	INS	AP10	SMOC2	NM_001166412	intron9-intron9
15	25080105	25080244	139	DEL	AP9	SNHG14/SNRPN	NM_001349455	intron1-intron1
15	25126697	25126738	41	DEL	AP12	SNHG14/SNRPN	NM_001349455	intron1-intron1
15	25621913	25621953	40	INS	AP4	SNHG14/UBE3A	NM_001354506	intron6-intron6
6	107975959	107976050	91	INS	AP10	SOBP	NM_018013	intron6-intron6
2	39306293	39306358	65	INS	AP1,AP10,AP11,AP2,AP9	SOS1	NM_005633	intron1-intron1
2	39315332	39315664	332	INS	AP3	SOS1	NM_005633	intron1-intron1
12	23795725	23796047	322	DEL	AP13,AP3,AP4	SOX5	NM_001330785	intron7-intron7
12	24430399	24430436	37	DEL	AP1,AP3	SOX5	NM_001261414	intron3-intron3
12	24460451	24460738	287	DEL	AP11,AP12	SOX5	NM_001261414	intron3-intron3
4	123936969	123937020	51	DEL	AP13,AP6	SPATA5	NM_001317799	intron10-intron10
14	88890014	88890172	158	DEL	AP10,AP11,AP12,AP2,AP6	SPATA7	NM_001040428	intron4-intron4
5	35734503	35734541	38	DEL	AP3	SPEF2	NM_024867	intron21-intron21
5	35751146	35751211	65	INS	AP3	SPEF2	NM_024867	intron23-intron23
5	35752762	35752934	172	INS	AP1,AP10,AP11,AP12,AP13,AP3,AP4,AP6	SPEF2	NM_024867	intron23-intron23
2	220321614	220321683	69	DEL	AP10,AP11,AP12,AP4,AP6	SPEG	NM_005876	intron6-intron6
5	147462158	147462478	320	DEL	AP11,AP12,AP13,AP3,AP4,AP6,AP9	SPINK5	NM_001127699	intron4-intron4

1	158629379	158629706	327	DEL	AP13	SPTA1	NM_003126	intron18-intron18
14	65289091	65289265	174	INS	AP1,AP10,AP11	SPTB	NM_001024858	intron1-intron1
14	77982337	77982368	31	DEL	AP3	SPTLC2	NM_004863	intron11-intron11
14	78036159	78036302	143	INS	AP10,AP12,AP13,AP3,AP4,AP9	SPTLC2	NM_004863	intron5-intron5
14	35457792	35457864	72	INS	AP10,AP4	SRP54	NM_001146282	intron1-intron1
3	133503773	133505091	1318	DEL	AP13,AP6,AP9	SRPRB/TF	NM_001354703	exon23-exon23
11	130048287	130048420	133	INS	AP11	ST14	NM_021978	intron1-intron1
1	44303678	44303709	31	DEL	AP12	ST3GAL3	NM_001270465	intron3-intron3
14	64504396	64504479	83	DEL	AP11	SYNE2	NM_182914	intron45-intron45
1	43857464	43857802	338	DEL	AP11,AP12	SZT2	NM_001365999	intron1-intron1
6	149632778	149632945	167	DEL	AP10,AP4,AP9	TAB2	NM_001292035	intron2-intron2
1	109614579	109614741	162	DEL	AP11,AP12,AP6,AP9	TAF13	NM_005645	intron2-intron2
17	80722613	80723330	717	DUP	AP4	TBCD	NM_005993	intron3-intron3
17	80856032	80856103	71	DEL	AP12,AP4,AP9	TBCD	NM_005993	intron17-intron17
17	80883791	80883967	176	DEL	AP11,AP12,AP4,AP6	TBCD	NM_005993	intron27-intron27
17	80896742	80896870	128	INS	AP12	TBCD	NM_005993	intron36-intron36
4	107056578	107063360	6782	DEL	AP6	TBCK	NM_001163435	intron23-intron23
X	9671259	9671309	50	DEL	AP13	TBL1X	NM_001139466	intron12-intron12
18	53146064	53146399	335	INS	AP1	TCF4/TCF4-AS1	NM_001243231	intron1-intron1
5	149746196	149746524	328	DEL	AP12,AP3,AP6	TCOF1	NM_001135243	intron3-intron3
11	12943736	12944013	277	DEL	AP9	TEAD1	NM_021961	intron10-intron10
14	102876618	102878079	1461	DEL	AP2	TECPR2	NM_001172631	intron4-intron4
11	121055970	121056912	942	DUP	AP12,AP4	TECTA	NM_005422	intron19-intron19
4	183438530	183438848	318	DEL	AP6	TENM3	NM_001080477	intron3-intron3
11	78516317	78516390	73	INS	AP1,AP12,AP3,AP4,AP6	TENM4	NM_001098816	exon15-intron15
2	74259596	74259670	74	INS	AP10	TET3	NM_001287491	intron3-intron3
3	24306051	24306095	44	DEL	AP1,AP10,AP11,AP12,AP3,AP4	THRB	NM_000461	intron2-intron2
9	71738120	71743355	5235	DEL	AP10,AP12,AP13,AP9	TJP2	NM_001170414	intron1-intron1
9	71777938	71778254	316	DEL	AP6	TJP2	NM_001170414	intron2-intron2
9	71827885	71827962	77	INS	AP1	TJP2	NM_001170414	intron3-intron3
11	61107791	61108134	343	DEL	AP10,AP3,AP6,AP9	TKFC	NM_001351978	intron5-intron5
3	53269955	53270028	73	INS	AP1,AP10,AP11	TKT	NM_001135055	intron4-intron4
4	166987133	166987265	132	INS	AP10,AP11	TLL1	NM_012464	intron16-intron16
4	167004312	167004909	597	DEL	AP12,AP3,AP4	TLL1	NM_012464	intron18-intron18
9	75315634	75315677	43	DEL	AP11,AP12,AP3	TMC1	NM_138691	intron8-intron8
1	165731218	165731498	280	DEL	AP12,AP13	TMCO1	NM_019026	intron2-intron2
7	12281703	12282031	328	DEL	AP12,AP6,AP9	TMEM106B	NM_001134232	exon8-exon8
3	14178184	14178860	676	DEL	AP12	TMEM43	NM_024334	intron10-intron10
9	117857735	117857940	205	DEL	AP12	TNC	NM_002160	intron1-intron1
3	189575902	189576227	325	DEL	AP10,AP2,AP4	TP63	NM_001329964	intron4-intron4
7	144382752	144382906	154	DEL	AP1,AP10,AP11,AP12,AP13,AP2,AP3,AP4,AP6,AP9	TPK1	NM_001350884	intron1-intron1
2	1449538	1449605	67	DEL	AP10	TPO	NM_000547	intron5-intron5
2	1471720	1472068	348	INS	AP13	TPO	NM_000547	intron7-intron7

16	2223693	2223754	61	DEL	AP9	TRAF7	NM_032271	intron11-intron11
8	140884949	140885158	209	DEL	AP10,AP11,AP12,AP4,AP9	TRAPPC9	NM_001160372	intron21-intron21
6	123622894	123622957	63	INS	AP4	TRDN	NM_006073	intron24-intron24
4	154095269	154095370	101	INS	AP10,AP11,AP3	TRIM2	NM_001375517	intron1-intron1
5	14347117	14347525	408	DUP	AP9	TRIO	NM_007118	intron11-intron11
8	116488223	116488555	332	DEL	AP10,AP11,AP12,AP3,AP6,AP9	TRPS1	NM_001282902	intron4-intron4
7	98550659	98550695	36	DEL	AP11,AP12,AP3	TRRAP	NM_001244580	intron38-intron38
14	81469120	81469435	315	DEL	AP1,AP11,AP13,AP3,AP6,AP9	TSHR	NM_001018036	intron1-intron1
14	81597047	81597367	320	DEL	AP3	TSHR	NM_000369	intron8-intron8
17	40103097	40103399	302	DUP	AP6	TTC25	NM_001350319	intron8-intron8
2	179621874	179622308	434	INS	AP9	TTN	NM_001267550	exon45-intron45
2	179621877	179621968	91	INS	AP3	TTN	NM_001256850	intron44-intron44
18	77792972	77793024	52	DEL	AP11,AP12,AP3,AP6	TXNL4A	NM_001305563	intron1-intron1
9	34202621	34202703	82	INS	AP11,AP13	UBAP1	NM_001171201	intron1-intron1
2	210651880	210652009	129	INS	AP10	UNC80	NM_001371986	intron5-intron5
2	210821028	210821246	218	INS	AP11,AP13,AP9	UNC80	NM_001371986	intron48-intron48
12	109544696	109544857	161	INS	AP11,AP3	UNG	NM_080911	intron6-intron6
11	17548692	17548737	45	DEL	AP12	USH1C	NM_001297764	intron5-intron5
1	216085794	216085859	65	DEL	AP12,AP9	USH2A	NM_206933	intron38-intron38
1	216185304	216185483	179	DEL	AP1,AP11,AP12,AP3	USH2A	NM_206933	intron32-intron32
1	216404580	216404656	76	INS	AP10,AP12	USH2A	NM_206933	intron14-intron14
15	50787443	50787498	55	DEL	AP13	USP8	NM_001128610	intron16-intron16
X	41057617	41057683	66	DEL	AP10,AP12,AP3	USP9X	NM_001039590	intron29-intron29
4	177661098	177661167	69	DEL	AP10,AP11,AP2,AP4,AP6	VEGFC	NM_005429	intron1-intron1
8	100655424	100655943	519	DEL	AP6	VPS13B	NM_017890	intron34-intron34
8	100827316	100827356	40	DEL	AP12,AP3,AP6	VPS13B	NM_017890	intron44-intron44
14	100810939	100810977	38	DEL	AP4	WARS1	NM_004184	intron7-intron7
6	110476734	110479774	3040	DEL	AP12	WASF1	NM_003931	intron3-intron3
2	20185028	20185287	259	DUP	AP9	WDR35	NM_001006657	intron2-intron2
17	80589465	80589784	319	INS	AP10,AP11	WDR45B	NM_019613	intron2-intron2
12	122436345	122436380	35	DEL	AP4	WDR66	NM_144668	intron19-intron19
11	32434272	32434601	329	INS	AP10	WT1	NM_001198552	intron4-intron4
16	78178326	78178388	62	INS	AP10	WWOX	NM_130791	intron4-intron4
16	78227442	78227881	439	INS	AP11,AP12,AP2,AP3,AP6,AP9	WWOX	NM_130791	intron5-intron5
16	78355183	78355251	68	DEL	AP12,AP3	WWOX	NM_001291997	intron4-intron4
16	78561889	78561925	36	INS	AP11	WWOX	NM_001291997	intron7-intron7
16	78653040	78653097	57	DEL	AP9	WWOX	NM_001291997	intron7-intron7
16	78692828	78693175	347	INS	AP3	WWOX	NM_001291997	intron7-intron7
2	31559687	31560028	341	INS	AP12,AP3	XDH	NM_000379	intron35-intron35
3	14196293	14196373	80	DEL	AP10	XPC	NM_001354727	intron9-intron9
1	180828201	180828255	54	INS	AP12,AP2,AP3,AP6,AP9	XPR1	NM_001135669	intron10-intron10
17	48427516	48427899	383	DEL	AP11	XYLT2	NM_022167	intron1-intron1
3	114466479	114466805	326	DEL	AP11,AP12,AP13,AP2,AP4,AP6,AP9	ZBTB20	NM_001164343	intron4-intron4

8	106664511	106664854	343	INS	AP10,AP4,AP6	ZFPM2	NM_001362836	intron4-intron4
8	106670524	106670848	324	DEL	AP10,AP4,AP6	ZFPM2	NM_001362836	intron4-intron4
10	80925715	80925776	61	INS	AP10,AP11	ZMIZ1	NM_020338	intron4-intron4
10	271571	271620	49	DEL	AP11	ZMYND11	NM_001370103	intron3-intron3
3	125062303	125062371	68	DEL	AP3,AP4	ZNF148	NM_001348433	intron1-intron1
16	49623939	49624002	63	INS	AP10,AP13,AP3	ZNF423	NM_001330533	intron3-intron3
16	49623952	49623991	39	INS	AP12	ZNF423	NM_001330533	intron3-intron3
16	49720684	49720721	37	INS	AP11,AP9	ZNF423	NM_015069	intron3-intron3
16	88478396	88478502	106	INS	AP11,AP3,AP6,AP9	ZNF469	NM_001367624	intron1-intron1
7	76044138	76044849	711	INS	AP3	ZP3	NM_007155	intron1-intron1

Supplementary Table E: 692 class-4 SVs and 477 genes found by 4 tools in 10 probands and at least one tool in 5 unaffected relatives

Chromosome	Start	End	Length	Type	Sample	Gene	Transcript	Location
15	67534470	67534807	337	DEL	AP10,AP11,AP13,AP4,AP9	AAGAB	NM_024666	intron1-intron1
10	101587738	101587964	226	INS	AP11,AP13,AP3	ABCC2	NM_000392	intron19-intron19
12	22016215	22016262	47	DEL	AP10,AP12,AP3	ABCC9	NM_020297	intron17-intron17
3	43735559	43735607	48	INS	AP6	ABHD5	NM_001365650	intron1-intron1
17	61565898	61566202	304	INS	AP10	ACE	NM_000789	intron15-intron16
2	74128664	74128709	45	DEL	AP9	ACTG2	NM_001199893	intron2-intron2
1	236900604	236900639	35	INS	AP10	ACTN2	NM_001103	intron9-intron9
2	158719091	158719404	313	DEL	AP10,AP11,AP12,AP3,AP6	ACVR1	NM_001105	intron1-intron1
12	52354757	52354890	133	DEL	AP11	ACVR1B	NM_004302	intron1-intron1
22	17665788	17665851	63	DEL	AP10,AP4,AP9	ADA2	NM_177405	intron4-intron4
15	58912854	58913175	321	DEL	AP1,AP10,AP11,AP12,AP13,AP2,AP3,AP4,AP6,AP[illegible]	ADAM10	NM_001110	intron11-intron11
8	38953618	38953877	259	DEL	AP4	ADAM9	NM_003816	intron20-intron20
15	100606978	100607180	202	DEL	AP11,AP3	ADAMTS17	NM_139057	intron15-intron15
15	100693156	100693198	42	INS	AP12,AP13	ADAMTS17	NM_139057	intron9-intron9
15	100702654	100702717	63	DEL	AP1	ADAMTS17	NM_139057	intron8-intron8
15	100879781	100879822	41	DEL	AP12	ADAMTS17	NM_139057	intron2-intron2
9	136414109	136414433	324	DEL	AP10,AP11	ADAMTSL2	NM_001145320	intron9-intron9
5	89860425	89860752	327	DEL	AP10,AP11,AP3,AP4,AP9	ADGRV1	NM_032119	intron1-intron1
5	89997830	89998001	171	INS	AP13	ADGRV1	NM_032119	intron34-intron34
10	76230334	76230481	147	INS	AP10	ADK	NM_001202450	intron6-intron6
5	132239876	132239918	42	DEL	AP9	AFF4	NM_014423	intron6-intron6
18	12331807	12331849	42	DEL	AP1	AFG3L2	NM_006796	intron16-intron16
15	86829966	86830016	50	INS	AP2	AGBL1	NM_152336	intron16-intron16
15	87177015	87177098	83	DEL	AP10,AP3	AGBL1	NM_152336	intron22-intron22
2	178287637	178288051	414	DEL	AP12,AP13,AP3,AP4,AP6	AGPS	NM_003659	intron2-intron2
2	178315691	178315735	44	INS	AP10,AP3,AP4,AP6	AGPS	NM_003659	intron8-intron8
2	178318718	178319078	360	INS	AP3	AGPS	NM_003659	intron8-intron8
2	178351456	178351821	365	INS	AP10	AGPS	NM_003659	intron11-intron11
1	966280	966317	37	DEL	AP6	AGRN	NM_001305275	intron2-intron2
1	243782739	243783742	1003	DEL	AP10,AP11,AP13,AP6	AKT3	NM_181690	intron5-intron5
15	101453075	101453386	311	DEL	AP12,AP3,AP4,AP9	ALDH1A3/LOC101927751	NM_000693	intron12-intron12
17	19571909	19572320	411	DEL	AP1,AP4	ALDH3A2	NM_001369137	intron9-intron9
2	112623971	112624016	45	INS	AP1,AP12	ANAPC1	NM_022662	intron7-intron7
4	113985876	113986308	432	DEL	AP13,AP9	ANK2	NM_001354269	intron1-intron1
4	114064436	114064513	77	INS	AP10,AP11,AP2,AP3	ANK2	NM_001354269	intron1-intron1
4	114135101	114135586	485	DEL	AP12	ANK2	NM_001354269	intron4-intron4
12	133304136	133304346	210	INS	AP6	ANKLE2	NM_015114	intron12-intron12
16	89408459	89408554	95	INS	AP9	ANKRD11	NM_001256182	intron3-intron3
7	36441175	36441946	771	DEL	AP2	ANLN	NM_001284301	intron3-intron3
3	43565778	43565935	157	INS	AP10,AP11,AP12,AP13	ANO10	NM_018075	intron11-intron11
11	26526328	26526359	31	DEL	AP10,AP12,AP3,AP9	ANO3	NM_001313726	intron5-intron5
11	26601639	26601957	318	INS	AP4	ANO3	NM_001313726	intron15-intron15
11	26679948	26680053	105	DEL	AP10,AP11,AP3	ANO3	NM_001313726	intron27-intron27
11	22262800	22262928	128	INS	AP6	ANO5	NM_001142649	intron9-intron9
11	22265278	22265450	172	DUP	AP13	ANO5	NM_001142649	intron9-intron9

4	80841014	80841057	43	DEL	AP1,AP10,AP12,AP3	ANTXR2	NM_001286781	intron16-intron16
4	80888046	80894103	6057	DEL	AP10,AP11,AP12,AP13,AP3,AP4,AP6,AP9	ANTXR2	NM_001286781	intron16-intron16
15	51222475	51222846	371	INS	AP9	AP4E1	NM_001252127	intron6-intron6
18	10462406	10462650	244	DEL	AP11	APCDD1	NM_153000	intron1-intron1
21	27252389	27252933	544	INS	AP11,AP13,AP4,AP9	APP	NM_001136016	exon17-txEnd
21	27374151	27374704	553	INV	AP10,AP11,AP12,AP13,AP3,AP4,AP6,AP9	APP	NM_001136016	intron6-intron6
22	51062326	51062358	32	DEL	AP10,AP11,AP12,AP13,AP4	ARSA	NM_000487	exon8-exon8
5	78276754	78277079	325	DEL	AP6	ARSB	NM_000046	intron1-intron1
5	78277729	78278053	324	DEL	AP13,AP3,AP9	ARSB	NM_000046	intron1-intron1
1	1465919	1466238	319	DEL	AP10	ATAD3A	NM_001170535	intron15-intron15
7	138420609	138420656	47	INS	AP10,AP11,AP3,AP4,AP6,AP9	ATP6V0A4	NM_130841	intron14-intron14
7	138468980	138469016	36	DEL	AP3	ATP6V0A4	NM_020632	intron2-intron2
7	138474596	138474778	182	INS	AP1,AP3	ATP6V0A4	NM_020632	intron1-intron1
13	52576728	52576883	155	INS	AP12,AP4	ATP7B	NM_000053	intron1-intron1
18	55380994	55381145	151	INS	AP12	ATP8B1	NM_001374385	intron2-intron2
14	92537352	92537402	50	INS	AP13,AP6	ATXN3	NM_001127696	intron8-exon9
13	31892037	31892186	149	INS	AP10,AP9	B3GLCT	NM_194318	intron13-intron13
15	73018135	73018218	83	DEL	AP6	BBS4	NM_001252678	intron7-intron7
7	33195321	33195693	372	INS	AP11	BBS9	NM_001348036	intron4-intron4
7	33403540	33403870	330	DEL	AP10,AP3,AP6	BBS9	NM_001348036	intron16-intron16
7	33576296	33576359	63	INS	AP3,AP4	BBS9	NM_001348036	intron21-intron21
18	60885835	60886060	225	DUP	AP10,AP3	BCL2	NM_000633	intron2-intron2
18	60932137	60932247	110	INS	AP10,AP11,AP12,AP13,AP3,AP4,AP9	BCL2	NM_000633	intron2-intron2
3	133133568	133133649	81	INS	AP12	BFSP2	NM_003571	intron1-intron1
7	34087397	34087538	141	INS	AP10,AP12,AP6,AP9	BMPER	NM_133468	intron8-intron8
10	88666182	88666526	344	DEL	AP10,AP11,AP12,AP13,AP3,AP6,AP9	BMPR1A	NM_004329	intron7-intron7
4	95870452	95870771	319	DEL	AP12,AP2	BMPR1B	NM_001203	intron2-intron2
2	203409935	203410074	139	INS	AP9	BMPR2	NM_001204	intron10-intron10
9	16682304	16682635	331	INS	AP13	BNC2	NM_001317939	intron2-intron2
9	16847393	16847519	126	DEL	AP9	BNC2	NM_001317939	intron1-intron1
7	134349999	134350428	429	DEL	AP1,AP11,AP12,AP13,AP6,AP9	BPGM	NM_001293085	intron3-intron3
13	32960701	32960733	32	DEL	AP6	BRCA2	NM_000059	intron24-intron24
6	105567842	105568182	340	DEL	AP1,AP10,AP11,AP12,AP2,AP4	BVES	NM_147147	intron5-intron5
11	73777311	73777371	60	INS	AP1,AP10,AP12,AP3,AP6,AP9	C2CD3	NM_001286577	intron24-intron24
5	40953955	40954031	76	INS	AP10,AP13,AP3,AP9	C7	NM_000587	intron9-intron9
1	57394750	57395002	252	INS	AP11,AP9	C8B	NM_000066	exon12-txEnd
12	2364956	2365257	301	DUP	AP10,AP11,AP13,AP3,AP6	CACNA1C	NM_000719	intron3-intron3
12	2467426	2467461	35	DEL	AP6	CACNA1C	NM_000719	intron3-intron3
12	2648366	2649066	700	DUP	AP10,AP11,AP12,AP13,AP6	CACNA1C	NM_000719	intron9-intron9
17	48665718	48665833	115	INS	AP11,AP12,AP9	CACNA1G	NM_001256324	intron9-intron9
10	18503075	18503589	514	DEL	AP11,AP9	CACNB2	NM_001167945	intron2-intron2
10	18731845	18731937	92	DEL	AP12	CACNB2	NM_001167945	intron3-intron3
2	152772499	152772542	43	DEL	AP4	CACNB4	NM_001330118	intron2-intron2
1	7044128	7044330	202	INS	AP13	CAMTA1	NM_001349608	intron2-intron2
1	7517099	7517132	33	DEL	AP6	CAMTA1	NM_001349608	intron4-intron4
17	77005260	77005315	55	DEL	AP3	CANT1	NM_001159772	intron1-intron1
6	17472276	17472595	319	DEL	AP1,AP3,AP4,AP6	CAP2	NM_001363533	intron4-intron4
13	111331380	111331414	34	INS	AP10	CARS2	NM_001352252	intron7-intron7
2	202146650	202149448	2798	DEL	AP11,AP12,AP3,AP4,AP6,AP9	CASP8	NM_001228	intron8-intron8

11	119124250	119124521	271	INS	AP4	CBL	NM_005188	intron2-intron2
18	57258186	57258527	341	DEL	AP10,AP11,AP13,AP2,AP4,AP9	CCBE1	NM_133459	intron2-intron2
14	91759356	91759922	566	DUP	AP9	CCDC88C	NM_001080414	intron23-intron23
3	45931754	45931796	42	INS	AP3	CCR9/LZTFL1	NM_001276378	intron2-intron2
11	833797	833877	80	INS	AP4	CD151	NM_004357	intron1-intron1
9	123257030	123259736	2706	DEL	AP12,AP3	CDK5RAP2	NM_001272039	intron12-intron12
4	85520548	85520584	36	DEL	AP10,AP11,AP13,AP3,AP9	CDS1	NM_001263	intron1-intron1
12	88520870	88520946	76	INS	AP1,AP10,AP12,AP2,AP3,AP4,AP6,AP9	CEP290	NM_025114	intron11-intron11
15	101051738	101052181	443	DEL	AP9	CERS3	NM_001290342	intron3-intron3
15	93515792	93516110	318	DEL	AP10,AP11,AP12,AP3,AP6,AP9	CHD2	NM_001271	intron19-intron19
X	109948662	109948810	148	INS	AP12,AP3,AP6	CHRDL1	NM_001143981	intron6-intron6
12	120201091	120201208	117	INS	AP3,AP6,AP9	CIT	NM_001206999	intron19-intron19
X	49821980	49822355	375	DUP	AP10,AP12,AP13,AP4,AP6	CLCN5	NM_001127898	intron4-intron4
1	16376513	16376601	88	INS	AP12,AP13,AP4,AP9	CLCNKB	NM_000085	intron10-intron10
3	190119029	190119097	68	DEL	AP13,AP2,AP6,AP9	CLDN16	NM_006580	intron1-intron1
8	87595254	87595291	37	INS	AP11,AP13,AP2,AP4	CNGB3	NM_019098	intron15-intron15
7	147283612	147283660	48	INS	AP10	CNTNAP2	NM_014141	intron11-intron11
7	148072862	148076323	3461	DEL	AP11,AP6	CNTNAP2	NM_014141	intron20-intron20
7	148106880	148107021	141	DEL	AP12,AP3	CNTNAP2	NM_014141	intron22-intron22
16	70553015	70553335	320	DEL	AP12	COG4	NM_001195139	intron2-intron2
7	107059159	107063047	3888	INV	AP12,AP9	COG5	NM_006348	intron6-intron6
10	71610168	71610493	325	INS	AP9	COL13A1	NM_001130103	intron3-intron3
10	105817205	105817407	202	INS	AP1,AP3,AP9	COL17A1	NM_000494	intron16-intron16
21	46825209	46825262	53	INS	AP10	COL18A1	NM_130445	intron1-intron1
21	46926665	46926716	51	DEL	AP11,AP12	COL18A1	NM_130445	intron38-intron38
21	46927370	46927416	46	DEL	AP11,AP12	COL18A1	NM_130445	intron38-intron38
7	94051083	94051122	39	DEL	AP12,AP13	COL1A2	NM_000089	intron38-intron38
4	109922542	109922646	104	INS	AP3	COL25A1	NM_198721	intron5-intron5
9	116937319	116937372	53	INS	AP1,AP12,AP3,AP6	COL27A1	NM_032888	intron3-intron3
2	189845085	189845145	60	DEL	AP12,AP13,AP3,AP6	COL3A1	NM_000090	intron1-intron1
13	110812500	110812816	316	DEL	AP2	COL4A1	NM_001845	intron49-intron49
13	111076800	111077141	341	INS	AP10,AP13,AP4	COL4A2	NM_001846	intron4-exon5
9	137690415	137690499	84	DEL	AP11,AP12,AP6	COL5A1	NM_000093	intron37-intron37
9	137720489	137720588	99	INS	AP3	COL5A1/LOC101448202	NM_000093	intron63-intron63
21	47410371	47410437	66	INS	AP10	COL6A1	NM_001848	intron13-intron13
21	47540037	47540098	61	INS	AP2	COL6A2	NM_058174	intron15-intron15
2	238252156	238252194	38	DEL	AP2,AP3	COL6A3	NM_057167	intron35-intron35
4	783681	783834	153	DEL	AP13	CPLX1	NM_006651	intron3-intron3
11	46319021	46319058	37	DEL	AP11	CREB3L1	NM_052854	intron1-intron1
16	3918271	3918499	228	DEL	AP10,AP11	CREBBP	NM_001079846	intron1-intron1
19	18800290	18800801	511	INS	AP9	CRTC1	NM_001098482	intron1-intron1
2	209008236	209008276	40	DEL	AP3	CRYGB/LOC100507443	NM_005210	intron2-intron2
8	19412052	19412094	42	INS	AP1,AP10,AP13	CSGALNACT1	NM_001130518	intron3-intron3
18	77471990	77472029	39	INS	AP10	CTDP1	NM_004715	intron6-intron6
18	77499783	77500111	328	DEL	AP11	CTDP1	NM_004715	intron12-intron12
10	67831137	67831189	52	INS	AP12,AP13,AP2,AP3	CTNNA3	NM_001127384	intron14-intron14
10	68049101	68049358	257	INS	AP1,AP10,AP12	CTNNA3	NM_001127384	intron12-intron12
10	68105980	68106018	38	DEL	AP10	CTNNA3	NM_001127384	intron12-intron12
10	68127616	68127651	35	DEL	AP4	CTNNA3	NM_001127384	intron12-intron12

10	68283944	68283988	44	INS	AP10,AP11,AP6,AP9	CTNNA3	NM_001127384	intron10-intron10
10	68326810	68326876	66	INS	AP11,AP6	CTNNA3	NM_001127384	intron10-intron10
10	69061723	69061823	100	DEL	AP10,AP2	CTNNA3	NM_001127384	intron5-intron5
16	88779319	88779697	378	DUP	AP10,AP13	CTU2	NM_001012759	intron7-intron7
5	64286061	64286400	339	DEL	AP10,AP6	CWC27	NM_001297644	intron12-intron12
10	104590841	104590877	36	DEL	AP10	CYP17A1	NM_000102	intron7-intron7
20	52779058	52779162	104	INS	AP6	CYP24A1	NM_000782	intron7-intron7
4	108865988	108866032	44	INS	AP1,AP6,AP9	CYP2U1	NM_183075	intron1-intron1
1	58071859	58072086	227	INS	AP10,AP9	DAB1	NM_021080	intron3-intron3
1	58343211	58343540	329	DEL	AP10,AP3,AP4,AP6,AP9	DAB1	NM_021080	intron2-intron2
1	58416995	58417046	51	INS	AP10	DAB1	NM_021080	intron1-intron1
1	100703693	100703741	48	DEL	AP11,AP3,AP4,AP6	DBT	NM_001918	intron2-intron2
18	50462564	50463030	466	DEL	AP6,AP9	DCC	NM_005215	intron5-intron5
18	50732492	50732582	90	INS	AP10,AP2,AP9	DCC	NM_005215	intron10-intron10
18	50925982	50926311	329	DEL	AP1,AP11,AP13,AP2,AP3,AP6,AP9	DCC	NM_005215	intron18-intron18
18	50930750	50930811	61	DEL	AP11,AP3	DCC	NM_005215	intron19-intron19
6	24325365	24327811	2446	DEL	AP12	DCDC2	NM_016356	intron2-intron2
9	32463882	32464063	181	INS	AP10,AP3,AP9	DDX58	NM_014314	intron16-intron16
9	32520816	32520853	37	DEL	AP6	DDX58	NM_014314	intron1-intron1
11	677323	677784	461	DEL	AP10,AP11	DEAF1	NM_001293634	intron7-intron7
11	696741	696884	143	INS	AP13	DEAF1/TMEM80	NM_001367390	intron1-intron1
12	125433332	125433417	85	DEL	AP11,AP12,AP4,AP9	DHX37	NM_032656	intron25-intron25
13	60611164	60611300	136	INS	AP10,AP12	DIAPH3/DIAPH3-AS1	NM_001042517	intron5-intron5
2	233111614	233111749	135	INS	AP10,AP11,AP12,AP13,AP2,AP3,AP4,AP9	DIS3L2	NM_152383	intron11-intron11
8	13364087	13364197	110	INS	AP4	DLC1	NM_182643	intron1-intron1
19	46273466	46273513	47	DEL	AP10	DM1-AS/DMPK	NM_001288765	exon13-exon13
X	31546269	31546356	87	DEL	AP13	DMD	NM_004021	intron12-intron12
X	31577472	31577787	315	DEL	AP13	DMD	NM_004021	intron12-intron12
X	32619776	32619807	31	DEL	AP12	DMD	NM_004006	intron12-intron12
X	32968065	32968130	65	INS	AP12	DMD	NM_004006	intron2-intron2
X	32987322	32989078	1756	DEL	AP6	DMD	NM_004006	intron2-intron2
X	33208588	33208914	326	DEL	AP6,AP9	DMD	NM_004006	intron1-intron1
X	33298072	33298170	98	INS	AP10	DMD	NM_000109	intron1-intron1
X	33302848	33303180	332	INS	AP13	DMD	NM_000109	intron1-intron1
15	51897686	51898050	364	DEL	AP10,AP11,AP9	DMXL2	NM_001174116	intron1-intron1
19	55671145	55671181	36	DEL	AP3	DNAAF3	NM_001256714	intron10-intron10
7	778688	778827	139	DEL	AP13	DNAAF5	NM_017802	intron2-intron2
7	21587709	21587830	121	DEL	AP10,AP13	DNAH11	NM_001277115	intron2-intron2
17	76461582	76461623	41	DEL	AP9	DNAH17	NM_173628	intron56-intron56
17	76505129	76505311	182	DEL	AP3	DNAH17	NM_173628	intron27-intron27
5	13827593	13828226	633	INS	AP9	DNAH5	NM_001369	intron38-intron38
5	13855311	13855627	316	DEL	AP3	DNAH5	NM_001369	intron30-intron30
5	13895085	13895384	299	DEL	AP13,AP3,AP4,AP6,AP9	DNAH5	NM_001369	intron15-intron15
17	11597929	11598067	138	DEL	AP12	DNAH9	NM_001372	intron22-intron22
7	157149904	157149989	85	INS	AP9	DNAJB6	NM_005494	intron1-intron1
10	69585408	69585508	100	INS	AP10,AP11,AP12,AP13,AP3,AP4,AP6	DNAJC12	NM_021800	intron1-intron1
1	63151822	63152167	345	DEL	AP1,AP10,AP11,AP2,AP3,AP4,AP9	DOCK7	NM_001271999	intron1-intron1
9	403897	403984	87	DEL	AP11	DOCK8	NM_203447	intron26-intron26
4	3477941	3477992	51	DEL	AP9	DOK7	NM_001164673	intron3-intron3

4	3484820	3484906	86	DEL	AP12	DOK7	NM_001164673	intron4-intron4
21	34949911	34949971	60	DEL	AP3,AP9	DONSON	NM_017613	exon10-exon10
7	153529762	153529833	71	DEL	AP10	DPP6	NM_001364497	intron2-intron2
7	154113834	154113928	94	INS	AP10,AP12	DPP6	NM_001364497	intron2-intron2
7	154153814	154155079	1265	DEL	AP2,AP3,AP6,AP9	DPP6	NM_001364497	intron3-intron3
7	154382272	154382628	356	INS	AP6	DPP6	NM_001364497	intron6-intron6
7	154446112	154446174	62	INS	AP2,AP3,AP4,AP9	DPP6	NM_001364497	intron7-intron7
7	154586520	154586579	59	INS	AP10,AP11,AP2,AP6,AP9	DPP6	NM_001364497	intron12-intron12
7	154609468	154609555	87	DEL	AP13	DPP6	NM_001364497	intron17-intron17
1	97599563	97599600	37	DEL	AP13	DPYD/DPYD-AS1	NM_000110	intron20-intron20
6	116642460	116642508	48	INS	AP1	DSE	NM_001374522	intron2-intron2
6	116750822	116751142	320	DEL	AP4,AP9	DSE	NM_001374522	intron4-intron4
6	56758354	56760959	2605	DEL	AP12,AP6	DST	NM_001144769	intron3-intron3
18	46593458	46593902	444	DUP	AP12,AP4	DYM	NM_001353210	intron16-intron16
11	103087929	103088089	160	DEL	AP11,AP12,AP13,AP2,AP3,AP4,AP6,AP9	DYNC2H1	NM_001080463	intron55-intron55
10	135182853	135182961	108	DEL	AP10,AP9	ECHS1	NM_004092	intron3-intron3
X	68865427	68865506	79	INS	AP10,AP12,AP13,AP3,AP6	EDA	NM_001005610	intron1-intron1
9	140563768	140564032	264	DUP	AP11,AP4	EHMT1	NM_001354611	intron1-intron1
9	140610465	140611020	555	DUP	AP6	EHMT1	NM_001354611	intron2-intron2
1	45408062	45408174	112	INS	AP12	EIF2B3	NM_020365	intron3-intron3
1	45417621	45417934	313	DEL	AP12	EIF2B3	NM_020365	intron3-intron3
6	53171004	53171062	58	DEL	AP1	ELOVL5	NM_001242828	intron1-intron1
1	29227718	29227878	160	DEL	AP10	EPB41	NM_001166005	intron1-intron1
2	212551246	212551550	304	DEL	AP1,AP11,AP12,AP4,AP6,AP9	ERBB4	NM_001042599	intron12-intron12
2	212700043	212700116	73	DEL	AP10	ERBB4	NM_001042599	intron3-intron3
2	213195781	213195886	105	INS	AP11,AP4,AP6,AP9	ERBB4	NM_001042599	intron1-intron1
8	27662507	27662840	333	DEL	AP13,AP2	ESCO2	NM_001017420	exon11-txEnd
15	76522853	76522942	89	INS	AP10,AP11,AP12,AP3,AP6,AP9	ETFA	NM_000126	intron10-intron10
12	12026411	12027153	742	DEL	AP11,AP13	ETV6	NM_001987	intron5-intron5
2	72739452	72739496	44	DEL	AP3	EXOC6B	NM_001321729	intron11-intron11
8	118980764	118981117	353	DEL	AP10,AP6	EXT1	NM_000127	intron1-intron1
8	72214745	72217809	3064	DEL	AP1	EYA1	NM_172058	intron6-intron6
6	65152734	65152780	46	DEL	AP11,AP9	EYS	NM_001292009	intron26-intron26
6	65185156	65185474	318	DEL	AP11,AP12,AP2,AP9	EYS	NM_001292009	intron26-intron26
6	65342504	65342557	53	INS	AP1,AP10,AP11,AP12,AP3,AP4,AP9	EYS	NM_001292009	intron22-intron22
6	65414374	65414406	32	DEL	AP2	EYS	NM_001292009	intron22-intron22
6	65768319	65768774	455	DEL	AP11,AP6	EYS	NM_001292009	intron12-intron12
6	65988220	65988285	65	INS	AP10,AP4	EYS	NM_001292009	intron12-intron12
6	66163974	66164302	328	INS	AP4	EYS	NM_001292009	intron5-intron5
6	66260027	66262028	2001	DEL	AP10,AP11,AP12,AP13,AP3,AP4,AP6,AP9	EYS	NM_001292009	intron2-intron2
6	66274841	66275173	332	DEL	AP10,AP12,AP3,AP4,AP9	EYS	NM_001292009	intron2-intron2
6	66399045	66404687	5642	DEL	AP10,AP6	EYS	NM_001292009	intron1-intron1
7	148563591	148563644	53	DEL	AP11	EZH2	NM_001203249	intron1-intron1
13	113790940	113791272	332	DEL	AP11,AP2,AP9	F10	NM_000504	intron2-intron2
6	6277349	6277615	266	DEL	AP10,AP12,AP6,AP9	F13A1	NM_000129	intron3-intron3
1	197011864	197012170	306	INS	AP10,AP4,AP6	F13B	NM_001994	intron10-intron10
15	31221929	31222010	81	DEL	AP9	FAN1	NM_014967	intron12-intron12
6	5348761	5349096	335	INS	AP3	FARS2	NM_001318872	intron1-intron1
5	150914537	150914576	39	INS	AP3	FAT2	NM_001447	intron11-intron11

22	45964509	45964705	196	DEL	AP11	FBLN1	NM_006486	intron14-intron14
3	192045373	192045705	332	INS	AP11	FGF12	NM_001377294	intron3-intron3
3	192063187	192063501	314	DEL	AP11,AP12,AP3,AP6	FGF12	NM_001377294	intron2-intron2
3	192358911	192359211	300	DEL	AP11,AP9	FGF12	NM_001377292	intron2-intron2
3	192429056	192429102	46	DEL	AP2	FGF12	NM_001377292	intron2-intron2
13	102455914	102455950	36	DEL	AP6	FGF14	NM_001321938	intron5-intron5
13	102813914	102814038	124	DEL	AP1,AP10,AP11,AP2,AP3,AP6	FGF14	NM_001321938	intron3-intron3
13	102886533	102886726	193	DEL	AP12,AP3	FGF14	NM_001321938	intron2-intron2
13	103026996	103027033	37	DEL	AP4	FGF14/FGF14-IT1	NM_001321938	intron2-intron2
3	187898593	187898733	140	INS	AP12	FLJ42393/LPP	NM_001375455	intron1-intron1
1	213039223	213039401	178	DEL	AP10,AP9	FLVCR1	NM_014053	intron2-intron2
1	240302567	240302662	95	DEL	AP12	FMN2	NM_001305424	intron2-intron2
1	240348222	240348519	297	DUP	AP11	FMN2	NM_001305424	intron4-intron4
1	240617000	240617325	325	DEL	AP13,AP2,AP3,AP9	FMN2	NM_001305424	intron17-intron17
3	71242345	71242654	309	DEL	AP10,AP4	FOXP1	NM_001244814	intron2-intron2
3	71376465	71376714	249	INS	AP11,AP12	FOXP1	NM_001244808	intron4-intron4
7	113953505	113953819	314	DEL	AP3,AP4	FOXP2	NR_033766	intron2-intron2
7	114227927	114228002	75	INS	AP13,AP3	FOXP2	NR_033766	intron6-intron6
1	74755166	74755291	125	DEL	AP1,AP10,AP11,AP12,AP13,AP2,AP3,AP4,AP6,AP[illegible]	FPGT-TNNI3K/TNNI3K	NM_015978	intron5-intron5
4	79159510	79159847	337	INS	AP4	FRAS1	NM_025074	intron3-intron3
4	79269120	79275203	6083	DEL	AP2,AP3,AP4,AP9	FRAS1	NM_025074	intron20-intron20
X	11953190	11959442	6252	DEL	AP10,AP12	FRMPD4	NM_001368395	intron3-intron3
2	49354879	49354959	80	DEL	AP10,AP2,AP3,AP9	FSHR	NM_000145	intron1-intron1
16	53823784	53823821	37	DEL	AP3,AP4	FTO	NM_001363891	intron1-intron1
14	66077925	66077980	55	DEL	AP10	FUT8	NM_004480	intron2-intron2
9	71665329	71665568	239	DEL	AP10,AP11,AP2,AP4,AP9	FXN	NM_000144	intron2-intron2
3	46018844	46018892	48	DEL	AP12,AP4,AP9	FYCO1	NM_024513	intron4-intron4
9	101209338	101209651	313	DEL	AP3	GABBR2	NM_005458	intron7-intron7
9	101309043	101311664	2621	DEL	AP13	GABBR2	NM_005458	intron2-intron2
4	47248362	47248405	43	DEL	AP3	GABRB1	NM_000812	intron4-intron4
2	166623167	166623301	134	INS	AP3	GALNT3	NM_004482	intron2-intron2
8	75375341	75375695	354	INS	AP10,AP11,AP3,AP6,AP9	GDAP1	NM_001362931	intron5-intron5
9	6629933	6629981	48	INS	AP3	GLDC	NM_000170	intron2-intron2
2	121680864	121680900	36	DEL	AP9	GLI2	NM_001371271	intron2-intron2
9	3953761	3953800	39	DEL	AP9	GLIS3	NM_001042413	intron4-intron4
18	11874561	11875039	478	INS	AP11,AP12,AP3	GNAL	NM_182978	intron10-intron10
12	102141413	102142069	656	INS	AP13	GNPTAB	NM_024312	intron20-intron20
12	65134833	65135013	180	DEL	AP1,AP10,AP12,AP13,AP3,AP6	GNS	NM_002076	intron6-intron6
13	93883564	93883670	106	DEL	AP10,AP13,AP4	GPC6	NM_005708	intron1-intron1
13	94105809	94106150	341	INS	AP11,AP12	GPC6	NM_005708	intron1-intron1
13	94163216	94163339	123	INS	AP4	GPC6	NM_005708	intron1-intron1
13	94392426	94392525	99	DEL	AP11,AP13	GPC6	NM_005708	intron2-intron2
13	95044888	95045033	145	DEL	AP13,AP2	GPC6	NM_005708	intron7-intron7
3	32153718	32153878	160	DEL	AP11,AP3,AP6	GPD1L	NM_015141	intron1-intron1
3	32201771	32202103	332	DEL	AP9	GPD1L	NM_015141	intron7-intron7
3	32203870	32204154	284	DEL	AP11,AP3	GPD1L	NM_015141	intron7-intron7
14	67012205	67012305	100	DEL	AP3	GPHN	NM_001024218	intron1-intron1
1	240675802	240675985	183	INS	AP12,AP9	GREM2	NM_022469	intron1-intron1
1	240708772	240708810	38	DEL	AP12	GREM2	NM_022469	intron1-intron1

11	105765515	105765834	319	DEL	AP11,AP13	GRIA4	NM_001077244	intron6-intron6
4	93499199	93499255	56	DEL	AP10,AP12	GRID2	NM_001510	intron1-intron1
4	93567358	93570163	2805	DUP	AP12,AP6	GRID2	NM_001510	intron2-intron2
4	94165633	94165777	144	DEL	AP1,AP3	GRID2	NM_001510	intron8-intron8
4	94662314	94662352	38	DEL	AP10,AP12,AP3,AP6	GRID2	NM_001510	intron14-intron14
6	102491666	102491710	44	INS	AP2	GRIK2	NM_001166247	intron14-intron14
12	13941602	13941706	104	DEL	AP1,AP11,AP12,AP13,AP3,AP4,AP6	GRIN2B	NM_000834	intron3-intron3
12	67116985	67117061	76	DEL	AP10,AP9	GRIP1	NM_001366723	intron1-intron1
4	42905161	42905849	688	DEL	AP11,AP9	GRXCR1	NM_001080476	intron1-intron1
19	49495744	49495780	36	INS	AP12	GYS1	NM_001161587	intron1-intron1
6	105260826	105263759	2933	DEL	AP10,AP13	HACE1	NM_001321083	intron6-intron6
15	28430088	28430347	259	INS	AP11,AP13,AP3,AP4,AP6,AP9	HERC2	NM_004667	intron56-intron56
15	72639532	72639885	353	INS	AP10,AP11,AP4	HEXA	NM_000520	intron10-intron10
5	73952303	73952348	45	DEL	AP12,AP9	HEXB	NM_001292004	intron1-intron1
7	81358335	81358611	276	DEL	AP1,AP11,AP12,AP13,AP3,AP4,AP9	HGF	NM_000601	intron8-intron8
6	143161427	143161511	84	DEL	AP10,AP11,AP12,AP13,AP2,AP3,AP4,AP6	HIVEP2	NM_006734	intron1-intron1
12	122289586	122289940	354	DEL	AP10,AP3,AP4,AP9	HPD	NM_002150	intron7-intron7
10	100496979	100497287	308	DEL	AP10,AP4	HPSE2	NM_021828	intron4-intron4
5	118853598	118853648	50	DEL	AP6	HSD17B4	NM_000414	intron17-intron17
20	2639732	2639788	56	INS	AP6	IDH3B	NM_001330763	intron11-intron11
3	129184476	129184785	309	DEL	AP12	IFT122	NM_001280545	intron8-intron8
6	160521754	160521811	57	DEL	AP10,AP11,AP13,AP3	IGF2R	NM_000876	intron45-intron45
8	42185315	42185635	320	DEL	AP1,AP10,AP11,AP12,AP13,AP3,AP4	IKBKB	NM_001242778	intron19-intron19
X	29337269	29337312	43	INS	AP3	IL1RAPL1	NM_014271	intron3-intron3
X	29353370	29353412	42	DEL	AP13	IL1RAPL1	NM_014271	intron3-intron3
X	29525484	29525672	188	INS	AP6	IL1RAPL1	NM_014271	intron5-intron5
10	6064768	6064842	74	DEL	AP6,AP9	IL2RA	NM_000417	intron3-intron3
10	6073407	6073458	51	DEL	AP9	IL2RA	NM_000417	intron1-intron1
10	6097377	6097875	498	DEL	AP11,AP6	IL2RA	NM_000417	intron1-intron1
6	76746502	76746583	81	DEL	AP12	IMPG1	NM_001563	intron2-intron2
3	100945069	100945110	41	DEL	AP2,AP4,AP6	IMPG2	NM_016247	exon19-exon19
15	40714966	40715155	189	INS	AP13	IVD	NM_001354598	intron11-intron11
16	87705204	87705260	56	DEL	AP3,AP4,AP6	JPH3	NR_073379	intron2-intron2
16	87726167	87726210	43	DEL	AP4	JPH3	NR_073379	intron4-intron4
1	210891707	210891858	151	INS	AP12,AP3,AP4	KCNH1	NM_002238	intron10-intron10
21	39050072	39050155	83	INS	AP1,AP10,AP3,AP9	KCNJ6	NM_002240	intron3-intron3
21	39076535	39076599	64	DEL	AP3	KCNJ6	NM_002240	intron3-intron3
21	39080012	39080499	487	INS	AP1,AP4	KCNJ6	NM_002240	intron3-intron3
21	39196245	39196338	93	INS	AP10,AP3,AP4,AP9	KCNJ6	NM_002240	intron2-intron2
21	39233186	39233234	48	DEL	AP12	KCNJ6	NM_002240	intron1-intron1
10	78800905	78801223	318	DEL	AP13	KCNMA1	NM_001322838	intron15-intron15
10	79325774	79325815	41	DEL	AP10	KCNMA1	NM_001014797	intron1-intron1
8	133315817	133315855	38	INS	AP12	KCNQ3	NM_001204824	intron1-intron1
6	73651391	73651454	63	DEL	AP11,AP3	KCNQ5	NM_001160130	intron1-intron1
18	24045976	24046054	78	DEL	AP6	KCTD1	NM_001142730	intron3-intron3
18	24134023	24134436	413	DEL	AP3	KCTD1	NM_198991	intron2-intron2
16	27724963	27725201	238	INS	AP10,AP11,AP9	KIAA0556/LOC100128079	NM_015202	intron13-intron13
5	93916283	93916629	346	DEL	AP11,AP13	KIAA0825	NM_001145678	intron2-intron2
5	61635355	61635683	328	INS	AP10	KIF2A	NM_001243952	intron2-intron2

2	10184251	10184297	46	DEL	AP11,AP13,AP3,AP6	KLF11	NM_003597	intron1-intron1
5	137022568	137023896	1328	DEL	AP10,AP11,AP12,AP3,AP9	KLHL3	NM_001257195	intron3-intron3
22	29515983	29516631	648	DUP	AP12	KREMEN1	NM_001039570	intron3-intron3
2	143800161	143800195	34	DEL	AP12	KYNU	NM_001199241	exon15-exon15
18	6952980	6953019	39	DEL	AP6	LAMA1	NM_005559	intron57-intron57
18	7073845	7073893	48	DEL	AP13	LAMA1	NM_005559	intron3-intron3
6	129737079	129737393	314	DEL	AP10,AP4	LAMA2	NM_000426	intron40-intron40
18	21490977	21491121	144	DEL	AP6,AP9	LAMA3	NM_001127717	intron54-intron54
3	45542650	45542991	341	INS	AP12,AP13	LARS2/LARS2-AS1	NM_001368263	intron14-intron14
12	65588606	65588714	108	INS	AP3	LEMD3	NM_001167614	intron1-intron1
1	65909628	65909898	270	DEL	AP4	LEPR	NM_001003680	intron2-intron2
1	66055933	66056278	345	INS	AP10,AP9	LEPR	NM_001003680	intron5-intron5
1	66079150	66079427	277	DEL	AP10,AP11,AP13,AP6,AP9	LEPR	NM_001003680	intron14-intron14
10	95545472	95546471	999	DEL	AP11,AP12,AP13,AP4,AP6,AP9	LGI1	NM_001308275	intron4-intron4
5	38511006	38511064	58	DEL	AP4	LIFR	NM_001127671	intron6-intron6
13	108867771	108867866	95	INS	AP3	LIG4	NM_001330595	exon1-intron1
15	77910866	77911241	375	DEL	AP12,AP13,AP9	LINGO1	NM_001301199	intron3-intron3
15	77991613	77992117	504	DUP	AP11	LINGO1	NM_001301198	intron1-intron1
15	78008485	78012583	4098	DEL	AP4	LINGO1	NM_001301197	intron1-intron1
16	964496	964829	333	DUP	AP12	LMF1	NM_001352021	intron3-intron3
12	106890951	106891290	339	INS	AP10,AP12	LOC100287944/POLR3B	NM_018082	intron25-intron25
2	166888719	166888872	153	INS	AP2,AP9	LOC102724058/SCN1A	NM_001165963	intron19-intron19
18	2997374	2997458	84	DEL	AP3,AP9	LPIN2	NM_014646	intron1-intron1
3	188304836	188305175	339	DEL	AP1,AP11,AP12,AP13,AP2,AP3,AP6,AP9	LPP	NM_001375455	intron5-intron5
3	188325158	188325201	43	DEL	AP12,AP13	LPP	NM_001375455	intron5-intron5
3	188473595	188473636	41	DEL	AP1,AP11,AP12,AP4,AP6	LPP	NM_001375455	intron7-intron7
4	151560894	151560951	57	DEL	AP6	LRBA	NM_001364905	intron37-intron37
4	151875129	151875222	93	INS	AP10,AP3,AP4	LRBA	NM_001364905	intron2-intron2
10	78255572	78261022	5450	DEL	AP1,AP4	LRMDA	NM_001305581	intron6-intron6
11	68156628	68156681	53	INS	AP6,AP9	LRP5	NM_001291902	intron6-intron6
11	68183100	68183411	311	DEL	AP11,AP12	LRP5	NM_001291902	intron12-intron12
22	21343986	21344028	42	DEL	AP4,AP6,AP9	LZTR1	NM_006767	intron7-intron7
7	1885062	1885190	128	DEL	AP12	MAD1L1	NM_001304525	intron3-intron3
7	2042674	2042742	68	INS	AP3,AP9	MAD1L1	NM_001013836	intron13-intron13
7	2172022	2172073	51	DEL	AP3	MAD1L1	NM_001013836	intron11-intron11
7	2200746	2200845	99	INS	AP10,AP11,AP13	MAD1L1	NM_001013836	intron10-intron10
11	47321174	47321237	63	INS	AP4,AP6,AP9	MADD	NM_001376651	intron22-intron22
7	77699495	77699535	40	DEL	AP2	MAGI2	NM_001301128	intron20-intron20
7	77959643	77959965	322	DEL	AP9	MAGI2	NM_001301128	intron9-intron9
7	78606779	78606825	46	DEL	AP11,AP2,AP4	MAGI2	NM_001301128	intron2-intron2
7	78953934	78954005	71	DEL	AP10	MAGI2	NM_001301128	intron1-intron1
11	95914918	95914962	44	DEL	AP1,AP10,AP12,AP3,AP9	MAML2	NM_032427	intron1-intron1
11	96001921	96003392	1471	DEL	AP6	MAML2	NM_032427	intron1-intron1
6	91237578	91237904	326	DEL	AP10,AP13,AP2,AP3,AP9	MAP3K7	NM_003188	intron12-intron12
18	32661959	32662279	320	DEL	AP11,AP2,AP3	MAPRE2	NM_001143826	intron3-intron3
5	68724041	68724494	453	DEL	AP12,AP6	MARVELD2	NM_001038603	intron3-intron3
3	168898582	168898634	52	INS	AP11,AP12,AP2	MECOM	NM_001205194	intron1-intron1
3	168909483	168909534	51	DEL	AP12	MECOM	NM_001205194	intron1-intron1
12	116715140	116715266	126	INS	AP10,AP11,AP12,AP3,AP4	MED13L	NM_015335	exon1-exon1

5	88032018	88032354	336	DEL	AP10,AP12,AP2,AP3,AP4,AP6	MEF2C	NM_001364353	intron4-intron4
5	88043136	88043477	341	DEL	AP1,AP3,AP4,AP6	MEF2C	NM_001364353	intron4-intron4
22	42140478	42140805	327	DEL	AP10,AP12,AP6,AP9	MEI1	NM_152513	intron12-intron12
7	116366194	116366314	120	INS	AP11,AP2	MET	NM_001324401	intron2-intron2
3	70013473	70013887	414	DEL	AP11	MITF	NM_001354604	intron9-intron9
3	154843069	154843129	60	DEL	AP2	MME	NM_001354642	intron8-intron8
21	33672989	33673073	84	INS	AP10	MRAP	NM_001285394	intron2-intron2
5	80039374	80039687	313	DEL	AP10,AP11,AP12,AP13,AP3,AP4,AP6,AP9	MSH3	NM_002439	intron11-intron11
8	15997255	15997314	59	DEL	AP12,AP9	MSR1	NM_138716	intron8-intron8
6	74216636	74217351	715	INS	AP10,AP3	MTO1	NM_001123226	exon13-exon13
9	113463932	113464153	221	DEL	AP1	MUSK	NM_001166280	intron5-intron5
12	102030823	102030908	85	INS	AP1,AP10,AP13,AP3,AP4,AP6	MYBPC1	NM_001254718	intron8-intron8
16	15869264	15869317	53	DEL	AP13,AP4	MYH11	NM_001040113	intron9-intron9
18	47694970	47698386	3416	DEL	AP12,AP13,AP4,AP9	MYO5B	NM_001080467	intron1-intron1
15	72217498	72217548	50	DEL	AP4,AP9	MYO9A	NM_006901	intron18-intron18
2	1826541	1826604	63	DEL	AP12,AP9	MYT1L	NM_001329845	intron21-intron21
2	1852059	1852109	50	DEL	AP12	MYT1L	NM_001329845	intron19-intron19
2	1865365	1865769	404	DUP	AP13,AP3,AP4,AP9	MYT1L	NM_001329845	intron18-intron18
2	2239127	2239202	75	DEL	AP2,AP3,AP4	MYT1L	NM_001329845	intron2-intron2
2	2266692	2266762	70	INS	AP11,AP3	MYT1L	NM_001329845	intron2-intron2
13	101894141	101896423	2282	DEL	AP6,AP9	NALCN	NM_001350748	intron11-intron11
2	240958947	240959271	324	DEL	AP1,AP10,AP11,AP12,AP9	NDUFA10	NR_136158	intron3-intron3
8	96103236	96103276	40	DEL	AP1,AP13,AP4,AP9	NDUFAF6	NR_148913	intron8-intron8
5	1812644	1812970	326	INS	AP3	NDUFS6	NM_004553	intron2-intron2
2	152476862	152476895	33	DEL	AP6	NEB	NM_001271208	intron72-intron72
18	55801583	55801914	331	DEL	AP11,AP12,AP13,AP3,AP6	NEDD4L	NM_001144967	intron1-intron1
17	29662292	29662413	121	INS	AP1,AP11,AP4,AP6	NF1	NM_000267	intron39-intron39
9	14388366	14388407	41	DEL	AP12	NFIB	NM_001190738	intron1-intron1
3	25802351	25802392	41	DEL	AP11,AP12,AP13,AP2,AP3,AP6,AP9	NGLY1	NM_018297	intron3-intron3
3	25824975	25825027	52	DEL	AP1,AP11,AP13,AP3,AP9	NGLY1	NM_001145294	intron1-intron1
X	17502755	17502824	69	DEL	AP12,AP6	NHS	NM_001291867	intron1-intron1
X	17520624	17520941	317	DEL	AP12,AP6	NHS	NM_001291867	intron1-intron1
15	23066394	23066531	137	DEL	AP1,AP13	NIPA1	NM_144599	intron1-intron1
1	10028615	10028764	149	INS	AP10,AP11,AP13,AP9	NMNAT1	NM_001297778	intron1-intron1
12	117797678	117797725	47	DEL	AP3	NOS1	NM_000620	intron1-intron1
12	100907587	100907626	39	DEL	AP9	NR1H4	NM_001206977	intron5-intron5
4	149287576	149287636	60	DUP	AP9	NR3C2	NM_000901	intron2-intron2
2	50360414	50360453	39	INS	AP3,AP4	NRXN1	NM_001330078	intron18-intron18
2	50602633	50602976	343	INS	AP12,AP13,AP3	NRXN1	NM_001330078	intron17-intron17
8	126305707	126305910	203	DEL	AP11,AP4	NSMCE2	NM_001349485	intron4-intron4
8	126336807	126337128	321	DEL	AP10,AP2	NSMCE2	NM_001349485	intron4-intron4
5	6616779	6617028	249	INS	AP10,AP3,AP9	NSUN2	NM_001193455	intron7-intron8
1	156806527	156806585	58	DEL	AP13,AP9	NTRK1	NM_001007792	intron1-intron1
9	87345367	87345682	315	DEL	AP12	NTRK2	NM_001007097	intron11-intron11
17	73208322	73208355	33	DEL	AP12	NUP85	NM_001330472	intron4-intron4
16	56774119	56774158	39	DEL	AP12	NUP93	NM_014669	intron1-intron1
17	744615	744964	349	DUP	AP10,AP12	NXN	NM_022463	intron1-intron1
17	801626	802222	596	DEL	AP12	NXN	NM_022463	intron1-intron1
15	28175794	28176197	403	INS	AP10,AP11	OCA2	NM_000275	intron18-intron18

11	132558588	132558880	292	DEL	AP1,AP11,AP6	OPCML	NM_001319103	intron1-intron1
11	133123705	133123753	48	DEL	AP10,AP11	OPCML	NM_001012393	intron1-intron1
10	13159416	13159452	36	DEL	AP11,AP9	OPTN	NM_001008211	intron7-intron7
12	80573892	80574059	167	DEL	AP1,AP4,AP9	OTOGL	NM_001368062	intron5-intron5
8	107417372	107417415	43	DEL	AP3,AP4,AP9	OXR1	NM_001198533	intron2-intron2
8	107434022	107434064	42	INS	AP11,AP2,AP3,AP4,AP9	OXR1	NM_001198533	intron2-intron2
8	107511099	107511428	329	DEL	AP12,AP13	OXR1	NM_001198533	intron2-intron2
8	107728562	107728893	331	DEL	AP10,AP2	OXR1	NM_001198533	intron11-intron11
8	107758527	107758572	45	INS	AP10,AP11,AP12,AP13,AP3,AP6	OXR1	NM_001198533	intron16-intron16
12	103261910	103261960	50	DEL	AP12,AP3	PAH	NM_000277	intron4-intron4
11	93871028	93871128	100	INS	AP10,AP3	PANX1	NM_015368	intron1-intron1
1	164540827	164540858	31	DEL	AP9	PBX1	NM_002585	intron2-intron2
1	164845205	164845248	43	DEL	AP11	PBX1	NM_001353131	intron7-intron7
1	164845207	164845247	40	DEL	AP9	PBX1	NM_001353131	intron7-intron7
1	164845209	164845249	40	DEL	AP9	PBX1	NM_001353131	intron7-intron7
1	164845210	164845254	44	DEL	AP10,AP2,AP3	PBX1	NM_001353131	intron7-intron7
1	164845234	164845267	33	INS	AP13	PBX1	NM_001353131	intron7-intron7
1	164845236	164845269	33	INS	AP13	PBX1	NM_001353131	intron7-intron7
11	66711484	66713291	1807	DEL	AP11	PC	NM_000920	intron2-intron2
10	55615665	55615707	42	INS	AP9	PCDH15	NM_001142771	intron29-intron29
10	55648055	55648195	140	INS	AP12	PCDH15	NM_001142771	intron27-intron27
10	55725753	55725841	88	DEL	AP4	PCDH15	NM_001142771	intron22-intron22
10	55761803	55761837	34	DEL	AP1	PCDH15	NM_001142771	intron21-intron21
10	55893665	55894123	458	DEL	AP3,AP4,AP6,AP9	PCDH15	NM_001142771	intron15-intron15
10	55958254	55958546	292	DEL	AP10	PCDH15	NM_001142771	intron11-intron11
10	56521450	56521481	31	DEL	AP11	PCDH15	NM_001142771	intron1-intron1
10	56682778	56684358	1580	DEL	AP12	PCDH15	NM_001354404	intron3-intron3
10	56762356	56762424	68	INS	AP10,AP13,AP2,AP3,AP4,AP6,AP9	PCDH15	NM_001354404	intron3-intron3
10	56767188	56773196	6008	INV	AP1,AP11,AP12	PCDH15	NM_001354404	intron3-intron3
10	56902361	56902705	344	INS	AP12,AP13	PCDH15	NM_001354404	intron3-intron3
10	56919102	56919134	32	DEL	AP9	PCDH15	NM_001354404	intron3-intron3
2	178676250	178676477	227	INS	AP6	PDE11A	NM_001077196	intron6-intron6
2	178810558	178810606	48	DEL	AP12	PDE11A	NM_016953	intron2-intron2
5	58274032	58274164	132	DEL	AP9	PDE4D	NM_001197223	intron6-intron6
5	58327202	58327238	36	DEL	AP6	PDE4D	NM_001197221	intron2-intron2
5	58869905	58869956	51	DEL	AP6	PDE4D	NM_001349242	intron1-intron1
17	79619831	79619886	55	DEL	AP10,AP11,AP12,AP13,AP3,AP4	PDE6G	NM_001365725	intron2-intron2
6	107723671	107723832	161	INS	AP12	PDSS2	NM_020381	intron1-intron1
7	92144388	92144420	32	DEL	AP3	PEX1	NM_000466	intron5-intron5
12	7348825	7348881	56	DEL	AP13	PEX5	NM_001351124	intron5-intron5
12	7355769	7356073	304	INS	AP13	PEX5	NM_001351124	intron8-exon9
6	13196491	13196527	36	DEL	AP1,AP13,AP4,AP6	PHACTR1	NM_001374582	intron8-intron8
11	46036239	46036594	355	DEL	AP1,AP12,AP2	PHF21A	NM_001352028	intron6-intron6
16	30763551	30764009	458	INS	AP11	PHKG2	NM_001172432	intron4-intron4
18	10757445	10757529	84	DEL	AP10,AP11,AP13,AP4,AP9	PIEZO2	NM_022068	intron25-intron25
18	10911130	10911467	337	INS	AP12,AP9	PIEZO2	NM_022068	intron3-intron4
18	10915930	10915978	48	DEL	AP1,AP11,AP9	PIEZO2	NM_022068	intron3-intron3
18	59728658	59728771	113	DEL	AP11,AP12,AP3,AP4,AP9	PIGN	NM_012327	intron29-intron29
18	59834520	59834564	44	DEL	AP10,AP12,AP2,AP3,AP9	PIGN	NM_012327	intron1-intron1

18	59852899	59852954	55	INS	AP2,AP3,AP4,AP9	PIGN	NM_012327	intron1-intron1
11	17195845	17196160	315	DEL	AP1,AP10,AP3,AP4	PIK3C2A	NM_001321378	intron2-intron2
11	17203155	17203196	41	DEL	AP12	PIK3C2A	NM_001321378	intron2-intron2
11	17215411	17215607	196	INS	AP11,AP4	PIK3C2A	NM_001321378	intron2-intron2
17	6422506	6422671	165	DEL	AP6	PITPNM3	NM_001165966	intron2-intron2
6	51517350	51517684	334	DEL	AP1,AP10,AP11,AP12,AP13,AP2,AP3,AP4,AP6,AP9	PKHD1	NM_138694	intron61-intron61
6	51739564	51745615	6051	DEL	AP10,AP11,AP3,AP6,AP9	PKHD1	NM_138694	intron46-intron46
6	51916612	51916655	43	DEL	AP10,AP3	PKHD1	NM_138694	intron21-intron21
12	32963604	32963921	317	DEL	AP10,AP11,AP12,AP13,AP4	PKP2	NM_004572	intron10-intron10
20	9149569	9149624	55	DEL	AP11,AP13,AP2,AP9	PLCB4	NM_001377134	intron2-intron2
20	9389100	9389168	68	INS	AP10,AP12	PLCB4	NM_001377134	intron21-intron21
6	161149397	161149509	112	INS	AP4	PLG	NM_000301	intron10-intron10
3	12351955	12352265	310	DEL	AP6	PPARG	NM_001354666	intron1-intron1
5	146026781	146026827	46	INS	AP10,AP11,AP3,AP6,AP9	PPP2R2B	NR_073526	intron5-intron5
5	146192048	146192081	33	DEL	AP4	PPP2R2B	NR_073526	intron1-intron1
5	146396113	146396177	64	INS	AP2,AP6	PPP2R2B	NM_001271900	intron2-intron2
4	102085246	102085818	572	INS	AP10,AP13	PPP3CA	NM_000944	intron2-intron2
1	3028033	3028072	39	INS	AP9	PRDM16	NM_022114	intron1-intron1
1	3079425	3079904	479	DUP	AP11,AP6	PRDM16	NM_022114	intron1-intron1
4	121808932	121809251	319	DEL	AP13,AP6	PRDM5	NM_001300823	intron2-intron2
5	122431836	122432034	198	INS	AP11,AP13,AP4	PRDM6	NM_001136239	intron2-intron2
12	42871283	42871330	47	DEL	AP11,AP4	PRICKLE1	NM_001144881	intron1-intron1
14	30295370	30295550	180	DEL	AP1	PRKD1	NM_001348390	intron1-intron1
10	53827672	53827991	319	DEL	AP6	PRKG1	NM_001098512	intron7-intron7
10	53828727	53828778	51	DEL	AP6	PRKG1	NM_001098512	intron7-intron7
6	161795618	161795800	182	INS	AP12,AP4,AP6	PRKN	NM_004562	intron10-intron10
6	161973419	161973479	60	DEL	AP12,AP3	PRKN	NM_004562	intron8-intron8
4	15987507	15987553	46	INS	AP1,AP10,AP11,AP3,AP9	PROM1	NM_001145849	intron20-intron20
17	40726730	40726764	34	DEL	AP12	PSMC3IP	NM_001256014	intron2-intron2
1	214610129	214610173	44	DEL	AP12	PTPN14	NM_005401	intron3-intron3
11	48120066	48120377	311	DEL	AP10,AP4	PTPRJ	NM_001098503	intron1-intron1
14	51408899	51409531	632	DEL	AP12,AP4	PYGL	NM_001163940	intron1-intron1
11	64526477	64526563	86	INS	AP10,AP11,AP12,AP4,AP9	PYGM	NM_001164716	intron1-intron1
3	25030883	25032243	1360	DEL	AP10,AP12,AP13,AP2,AP3,AP4,AP6	RARB	NM_001290216	intron2-intron2
3	25508990	25509033	43	DEL	AP3,AP9	RARB	NM_001290216	intron5-intron5
6	88285774	88286250	476	DEL	AP3	RARS2	NM_001318785	intron1-intron1
10	112480400	112480454	54	DEL	AP3	RBM20	NM_001134363	intron1-intron1
7	103463072	103463399	327	DEL	AP1,AP10,AP11,AP12,AP13,AP2,AP4,AP6,AP9	RELN	NM_173054	intron3-intron3
7	103470504	103470566	62	INS	AP10,AP6	RELN	NM_173054	intron3-intron3
7	103612297	103612333	36	DEL	AP13	RELN	NM_173054	intron1-intron1
1	8446629	8446963	334	INS	AP10,AP11	RERE	NM_001042682	intron2-intron2
1	8729018	8729101	83	DEL	AP10,AP4,AP6	RERE	NM_001042681	intron1-intron1
6	72863851	72873534	9683	DEL	AP3	RIMS1	NM_014989	intron4-intron4
3	149593684	149593795	111	INS	AP1,AP10,AP11,AP6,AP9	RNF13	NM_007282	intron5-intron5
4	1086775	1086826	51	DEL	AP12,AP3	RNF212	NM_001366918	intron3-intron3
3	77213923	77213975	52	INS	AP12	ROBO2	NM_001290039	intron2-intron2
15	61216539	61216889	350	INS	AP11	RORA	NM_134261	intron1-intron1
15	61346046	61346090	44	INS	AP4	RORA	NM_134261	intron1-intron1
15	60835369	60835400	31	DEL	AP4	RORA/RORA-AS1	NM_134262	intron1-intron1

16	53713454	53713556	102	DEL	AP1,AP4	RPGRIP1L	NM_001127897	intron6-intron6
2	89001138	89001220	82	INS	AP12,AP13,AP3,AP4,AP6	RPIA	NM_144563	intron3-intron3
2	89029234	89032289	3055	DEL	AP3,AP4	RPIA	NM_144563	intron4-intron4
11	14348314	14348634	320	DEL	AP12,AP13,AP4,AP9	RRAS2	NM_001177315	intron1-intron1
1	38077346	38077420	74	DEL	AP10,AP12,AP13,AP3,AP6,AP9	RSPO1	NM_001038633	intron8-exon9
16	57231835	57231881	46	DEL	AP1,AP11	RSPRY1	NM_001305182	intron1-intron1
16	57234574	57234622	48	INS	AP3	RSPRY1	NM_001305182	intron1-intron1
16	57252625	57252951	326	DEL	AP1,AP11,AP12	RSPRY1	NM_001305163	intron8-intron8
3	158116907	158117252	345	DEL	AP12,AP13,AP4,AP6,AP9	RSRC1	NM_001271834	intron5-intron5
3	158223547	158223903	356	INS	AP13	RSRC1	NM_001271834	intron6-intron6
1	237449855	237450067	212	INS	AP10,AP3,AP6,AP9	RYR2	NM_001035	intron2-intron2
1	237679021	237679178	157	INS	AP3,AP4	RYR2	NM_001035	intron24-intron24
8	119447049	119447361	312	DEL	AP6	SAMD12	NR_146234	intron3-intron3
8	119618005	119618046	41	DEL	AP11,AP6	SAMD12	NR_146234	intron1-intron1
2	200312455	200312679	224	DEL	AP13	SATB2	NM_001172517	intron3-intron3
11	10292740	10293846	1106	DEL	AP3	SBF2	NM_030962	intron1-intron1
3	39011492	39011531	39	DEL	AP12	SCN11A	NM_001349253	intron2-intron2
2	166905653	166905728	75	DEL	AP2	SCN1A	NM_001165963	intron9-intron9
2	166014692	166016744	2052	DEL	AP10,AP4,AP6	SCN3A	NM_001081676	intron9-intron9
1	243643592	243644217	625	DUP	AP3	SDCCAG8	NM_001350246	intron18-intron18
14	39563442	39563495	53	DEL	AP1	SEC23A	NM_006364	intron2-intron2
6	108266114	108266421	307	DEL	AP1,AP10,AP11,AP3,AP4,AP9	SEC63	NM_007214	intron1-intron1
6	108274959	108275056	97	DEL	AP1	SEC63	NM_007214	intron1-intron1
1	26134788	26134833	45	DEL	AP2,AP3	SELENON	NM_020451	intron4-intron4
6	158548272	158549079	807	DEL	AP1,AP11,AP2,AP3,AP4	SERAC1	NM_032861	intron10-intron10
18	42448667	42448712	45	DEL	AP10,AP4	SETBP1	NM_001130110	intron2-intron2
5	155777255	155777300	45	INS	AP12,AP6	SGCD	NM_000337	intron3-intron3
4	2810478	2810773	295	DEL	AP10	SH3BP2	NM_001122681	intron1-intron1
5	171795464	171795672	208	DEL	AP3	SH3PXD2B	NM_001308175	intron6-intron6
5	148401439	148401598	159	DEL	AP11,AP2,AP3	SH3TC2	NM_024577	intron12-intron12
22	51154450	51154532	82	DEL	AP13	SHANK3	NM_001372044	intron21-intron21
X	50538040	50538088	48	DEL	AP12	SHROOM4	NM_020717	intron1-intron1
15	48550269	48550591	322	DEL	AP13,AP3,AP6	SLC12A1	NM_000338	intron16-intron16
15	48562946	48563157	211	INS	AP10,AP4	SLC12A1	NM_000338	intron19-intron19
X	73679069	73679217	148	INS	AP10,AP11,AP9	SLC16A2	NM_006517	intron1-intron1
1	169442962	169443308	346	INS	AP4	SLC19A2	NM_006996	intron2-intron2
5	36649429	36649465	36	DEL	AP10,AP12,AP13	SLC1A3	NM_001289939	intron2-intron2
14	92941046	92941091	45	DEL	AP1	SLC24A4	NM_153648	intron12-intron12
2	172664711	172664822	111	DEL	AP10	SLC25A12	NM_003705	intron13-intron13
7	95764104	95764144	40	INS	AP12	SLC25A13	NM_014251	intron14-intron14
1	108733315	108737228	3913	DEL	AP9	SLC25A24	NM_213651	txStart-intron1
3	66335921	66336865	944	INS	AP11	SLC25A26	NM_173471	intron6-intron6
4	9927873	9927914	41	DEL	AP9	SLC2A9	NM_020041	intron6-intron6
4	9952298	9952551	253	DEL	AP10,AP12,AP3,AP4	SLC2A9	NM_020041	intron5-intron5
4	9982897	9983287	390	INS	AP11	SLC2A9	NM_020041	intron4-intron4
5	33973937	33973977	40	DEL	AP12,AP6	SLC45A2	NM_016180	intron2-intron2
5	1206475	1206546	71	DEL	AP13,AP9	SLC6A19	NM_001003841	intron1-intron1
3	45831051	45831106	55	DEL	AP1,AP6	SLC6A20	NM_020208	intron1-intron1
5	492292	492352	60	DEL	AP4	SLC9A3	NM_001284351	intron1-intron1

5	505488	505568	80	DEL	AP6	SLC9A3	NM_001284351	intron1-intron1
18	2748478	2748515	37	DEL	AP1,AP12,AP2,AP3,AP4,AP6	SMCHD1	NM_015295	intron30-intron30
6	168905864	168905942	78	DEL	AP10,AP6	SMOC2	NM_001166412	intron1-intron1
15	25080105	25080244	139	DEL	AP9	SNHG14/SNRPN	NM_001349455	intron1-intron1
15	25126697	25126738	41	DEL	AP12	SNHG14/SNRPN	NM_001349455	intron1-intron1
15	25621913	25621953	40	INS	AP4	SNHG14/UBE3A	NM_001354506	intron6-intron6
6	107975959	107976050	91	INS	AP10	SOBP	NM_018013	intron6-intron6
2	39306293	39306358	65	INS	AP1,AP10,AP11,AP2,AP9	SOS1	NM_005633	intron1-intron1
2	39315332	39315664	332	INS	AP3	SOS1	NM_005633	intron1-intron1
12	23795725	23796047	322	DEL	AP13,AP3,AP4	SOX5	NM_001330785	intron7-intron7
12	24430399	24430436	37	DEL	AP1,AP3	SOX5	NM_001261414	intron3-intron3
12	24460451	24460738	287	DEL	AP11,AP12	SOX5	NM_001261414	intron3-intron3
4	123936969	123937020	51	DEL	AP13,AP6	SPATA5	NM_001317799	intron10-intron10
14	88890014	88890172	158	DEL	AP10,AP11,AP12,AP2,AP6	SPATA7	NM_001040428	intron4-intron4
5	35751146	35751211	65	INS	AP3	SPEF2	NM_024867	intron23-intron23
5	35752762	35752934	172	INS	AP1,AP10,AP11,AP12,AP13,AP3,AP4,AP6	SPEF2	NM_024867	intron23-intron23
2	220321614	220321683	69	DEL	AP10,AP11,AP12,AP4,AP6	SPEG	NM_005876	intron6-intron6
5	147462158	147462478	320	DEL	AP11,AP12,AP13,AP3,AP4,AP6,AP9	SPINK5	NM_001127699	intron4-intron4
1	158629379	158629706	327	DEL	AP13	SPTA1	NM_003126	intron18-intron18
14	65289091	65289265	174	INS	AP1,AP10,AP11	SPTB	NM_001024858	intron1-intron1
14	77982337	77982368	31	DEL	AP3	SPTLC2	NM_004863	intron11-intron11
14	78036159	78036302	143	INS	AP10,AP12,AP13,AP3,AP4,AP9	SPTLC2	NM_004863	intron5-intron5
14	35457792	35457864	72	INS	AP10,AP4	SRP54	NM_001146282	intron1-intron1
3	133503773	133505091	1318	DEL	AP13,AP6,AP9	SRPRB/TF	NM_001354703	exon23-exon23
11	130048287	130048420	133	INS	AP11	ST14	NM_021978	intron1-intron1
1	44303678	44303709	31	DEL	AP12	ST3GAL3	NM_001270465	intron3-intron3
14	64504396	64504479	83	DEL	AP11	SYNE2	NM_182914	intron45-intron45
1	43857464	43857802	338	DEL	AP11,AP12	SZT2	NM_001365999	intron1-intron1
6	149632778	149632945	167	DEL	AP10,AP4,AP9	TAB2	NM_001292035	intron2-intron2
1	109614579	109614741	162	DEL	AP11,AP12,AP6,AP9	TAF13	NM_005645	intron2-intron2
17	80722613	80723330	717	DUP	AP4	TBCD	NM_005993	intron3-intron3
17	80856032	80856103	71	DEL	AP12,AP4,AP9	TBCD	NM_005993	intron17-intron17
17	80883791	80883967	176	DEL	AP11,AP12,AP4,AP6	TBCD	NM_005993	intron27-intron27
17	80896742	80896870	128	INS	AP12	TBCD	NM_005993	intron36-intron36
4	107056578	107063360	6782	DEL	AP6	TBCK	NM_001163435	intron23-intron23
X	9671259	9671309	50	DEL	AP13	TBL1X	NM_001139466	intron12-intron12
18	53146064	53146399	335	INS	AP1	TCF4/TCF4-AS1	NM_001243231	intron1-intron1
5	149746196	149746524	328	DEL	AP12,AP3,AP6	TCOF1	NM_001135243	intron3-intron3
11	12943736	12944013	277	DEL	AP9	TEAD1	NM_021961	intron10-intron10
14	102876618	102878079	1461	DEL	AP2	TECPR2	NM_001172631	intron4-intron4
11	121055970	121056912	942	DUP	AP12,AP4	TECTA	NM_005422	intron19-intron19
4	183438530	183438848	318	DEL	AP6	TENM3	NM_001080477	intron3-intron3
11	78516317	78516390	73	INS	AP1,AP12,AP3,AP4,AP6	TENM4	NM_001098816	exon15-intron15
3	24306051	24306095	44	DEL	AP1,AP10,AP11,AP12,AP3,AP4	THRB	NM_000461	intron2-intron2
9	71738120	71743355	5235	DEL	AP10,AP12,AP13,AP9	TJP2	NM_001170414	intron1-intron1
9	71777938	71778254	316	DEL	AP6	TJP2	NM_001170414	intron2-intron2
9	71827885	71827962	77	INS	AP1	TJP2	NM_001170414	intron3-intron3
11	61107791	61108134	343	DEL	AP10,AP3,AP6,AP9	TKFC	NM_001351978	intron5-intron5
3	53269955	53270028	73	INS	AP1,AP10,AP11	TKT	NM_001135055	intron4-intron4

4	166987133	166987265	132	INS	AP10,AP11	TLL1	NM_012464	intron16-intron16
4	167004312	167004909	597	DEL	AP12,AP3,AP4	TLL1	NM_012464	intron18-intron18
9	75315634	75315677	43	DEL	AP11,AP12,AP3	TMC1	NM_138691	intron8-intron8
1	165731218	165731498	280	DEL	AP12,AP13	TMCO1	NM_019026	intron2-intron2
7	12281703	12282031	328	DEL	AP12,AP6,AP9	TMEM106B	NM_001134232	exon8-exon8
3	14178184	14178860	676	DEL	AP12	TMEM43	NM_024334	intron10-intron10
9	117857735	117857940	205	DEL	AP12	TNC	NM_002160	intron1-intron1
3	189575902	189576227	325	DEL	AP10,AP2,AP4	TP63	NM_001329964	intron4-intron4
7	144382752	144382906	154	DEL	AP1,AP10,AP11,AP12,AP13,AP2,AP3,AP4,AP6,AP[illegible]	TPK1	NM_001350884	intron1-intron1
2	1449538	1449605	67	DEL	AP10	TPO	NM_000547	intron5-intron5
2	1471720	1472068	348	INS	AP13	TPO	NM_000547	intron7-intron7
16	2223693	2223754	61	DEL	AP9	TRAF7	NM_032271	intron11-intron11
8	140884949	140885158	209	DEL	AP10,AP11,AP12,AP4,AP9	TRAPPC9	NM_001160372	intron21-intron21
6	123622894	123622957	63	INS	AP4	TRDN	NM_006073	intron24-intron24
4	154095269	154095370	101	INS	AP10,AP11,AP3	TRIM2	NM_001375517	intron1-intron1
5	14347117	14347525	408	DUP	AP9	TRIO	NM_007118	intron11-intron11
8	116488223	116488555	332	DEL	AP10,AP11,AP12,AP3,AP6,AP9	TRPS1	NM_001282902	intron4-intron4
7	98550659	98550695	36	DEL	AP11,AP12,AP3	TRRAP	NM_001244580	intron38-intron38
14	81469120	81469435	315	DEL	AP1,AP11,AP13,AP3,AP6,AP9	TSHR	NM_001018036	intron1-intron1
14	81597047	81597367	320	DEL	AP3	TSHR	NM_000369	intron8-intron8
17	40103097	40103399	302	DUP	AP6	TTC25	NM_001350319	intron8-intron8
2	179621877	179621968	91	INS	AP3	TTN	NM_001256850	intron44-intron44
18	77792972	77793024	52	DEL	AP11,AP12,AP3,AP6	TXNL4A	NM_001305563	intron1-intron1
9	34202621	34202703	82	INS	AP11,AP13	UBAP1	NM_001171201	intron1-intron1
2	210651880	210652009	129	INS	AP10	UNC80	NM_001371986	intron5-intron5
2	210821028	210821246	218	INS	AP11,AP13,AP9	UNC80	NM_001371986	intron48-intron48
12	109544696	109544857	161	INS	AP11,AP3	UNG	NM_080911	intron6-intron6
11	17548692	17548737	45	DEL	AP12	USH1C	NM_001297764	intron5-intron5
1	216085794	216085859	65	DEL	AP12,AP9	USH2A	NM_206933	intron38-intron38
1	216185304	216185483	179	DEL	AP1,AP11,AP12,AP3	USH2A	NM_206933	intron32-intron32
1	216404580	216404656	76	INS	AP10,AP12	USH2A	NM_206933	intron14-intron14
15	50787443	50787498	55	DEL	AP13	USP8	NM_001128610	intron16-intron16
X	41057617	41057683	66	DEL	AP10,AP12,AP3	USP9X	NM_001039590	intron29-intron29
4	177661098	177661167	69	DEL	AP10,AP11,AP2,AP4,AP6	VEGFC	NM_005429	intron1-intron1
8	100655424	100655943	519	DEL	AP6	VPS13B	NM_017890	intron34-intron34
8	100827316	100827356	40	DEL	AP12,AP3,AP6	VPS13B	NM_017890	intron44-intron44
6	110476734	110479774	3040	DEL	AP12	WASF1	NM_003931	intron3-intron3
2	20185028	20185287	259	DUP	AP9	WDR35	NM_001006657	intron2-intron2
17	80589465	80589784	319	INS	AP10,AP11	WDR45B	NM_019613	intron2-intron2
12	122436345	122436380	35	DEL	AP4	WDR66	NM_144668	intron19-intron19
11	32434272	32434601	329	INS	AP10	WT1	NM_001198552	intron4-intron4
16	78178326	78178388	62	INS	AP10	WWOX	NM_130791	intron4-intron4
16	78227442	78227881	439	INS	AP11,AP12,AP2,AP3,AP6,AP9	WWOX	NM_130791	intron5-intron5
16	78355183	78355251	68	DEL	AP12,AP3	WWOX	NM_001291997	intron4-intron4
16	78692828	78693175	347	INS	AP3	WWOX	NM_001291997	intron7-intron7
2	31559687	31560028	341	INS	AP12,AP3	XDH	NM_000379	intron35-intron35
3	14196293	14196373	80	DEL	AP10	XPC	NM_001354727	intron9-intron9
1	180828201	180828255	54	INS	AP12,AP2,AP3,AP6,AP9	XPR1	NM_001135669	intron10-intron10
17	48427516	48427899	383	DEL	AP11	XYLT2	NM_022167	intron1-intron1

3	114466479	114466805	326	DEL	AP11,AP12,AP13,AP2,AP4,AP6,AP9	ZBTB20	NM_001164343	intron4-intron4
8	106664511	106664854	343	INS	AP10,AP4,AP6	ZFPM2	NM_001362836	intron4-intron4
8	106670524	106670848	324	DEL	AP10,AP4,AP6	ZFPM2	NM_001362836	intron4-intron4
10	80925715	80925776	61	INS	AP10,AP11	ZMIZ1	NM_020338	intron4-intron4
3	125062303	125062371	68	DEL	AP3,AP4	ZNF148	NM_001348433	intron1-intron1
16	49623939	49624002	63	INS	AP10,AP13,AP3	ZNF423	NM_001330533	intron3-intron3
16	49623952	49623991	39	INS	AP12	ZNF423	NM_001330533	intron3-intron3
16	49720684	49720721	37	INS	AP11,AP9	ZNF423	NM_015069	intron3-intron3
16	88478396	88478502	106	INS	AP11,AP3,AP6,AP9	ZNF469	NM_001367624	intron1-intron1
7	76044138	76044849	711	INS	AP3	ZP3	NM_007155	intron1-intron1

Supplementary Table F: 404 SVs and 325 genes found by 4 tools in 5 unaffected relatives

Chromosome	Start	End	Length	Type	Sample	Gene	Transcript	Location
15	67534473	67534808	335	DEL	AC6,AC9,AC13	AAGAB	NM_024666	intron1-intron1
10	101587739	101587960	221	INS	AC13	ABCC2	NM_000392	intron19-intron19
12	22016216	22016263	47	DEL	AC9,AC13	ABCC9	NM_020297	intron17-intron17
12	22047472	22047576	104	DEL	AC6,AC9	ABCC9	NM_020297	intron11-intron11
3	43735558	43735609	51	INS	AC6	ABHD5	NM_001365650	intron1-intron1
2	74128660	74128710	50	DEL	AC9	ACTG2	NM_001199893	intron2-intron2
1	236876894	236878365	1471	DUP	AC13	ACTN2	NM_001103	intron1-intron1
2	158719092	158719404	312	DEL	AC6,AC13	ACVR1	NM_001105	intron1-intron1
22	17665794	17665850	56	DEL	AC13	ADA2	NM_177405	intron4-intron4
15	58912853	58913174	321	DEL	AC2,AC6,AC13	ADAM10	NM_001110	intron11-intron11
8	38902903	38903082	179	INS	AC9,AC13	ADAM9	NM_003816	intron12-intron12
15	100606982	100607180	198	DEL	AC6	ADAMTS17	NM_139057	intron15-intron15
15	100693158	100693199	41	INS	AC2	ADAMTS17	NM_139057	intron9-intron9
4	73418754	73418842	88	DEL	AC2	ADAMTS3	NM_014243	intron2-intron2
9	136414108	136414435	327	DEL	AC9,AC13	ADAMTSL2	NM_001145320	intron9-intron9
5	89860428	89860749	321	DEL	AC6,AC9	ADGRV1	NM_032119	intron1-intron1
5	89997830	89998001	171	INS	AC13	ADGRV1	NM_032119	intron34-intron34
10	76230336	76230486	150	INS	AC6	ADK	NM_001202450	intron6-intron6
15	86805310	86805346	36	INS	AC12	AGBL1	NM_152336	intron9-intron9
2	178287637	178288051	414	DEL	AC6	AGPS	NM_003659	intron2-intron2
2	178315692	178315763	71	INS	AC13	AGPS	NM_003659	intron8-intron8
15	101453076	101453384	308	DEL	AC13	ALDH1A3/LOC101927751	NM_000693	intron12-intron12
15	85397465	85397801	336	INS	AC2	ALPK3	NM_020778	intron5-intron5
4	113985883	113986344	461	DEL	AC9	ANK2	NM_001354269	intron1-intron1
4	114018256	114018583	327	INS	AC12	ANK2	NM_001354269	intron1-intron1
4	114064436	114064510	74	INS	AC2,AC13	ANK2	NM_001354269	intron1-intron1
4	114135100	114135584	484	DEL	AC13	ANK2	NM_001354269	intron4-intron4
3	43565778	43565929	151	INS	AC2,AC13	ANO10	NM_018075	intron11-intron11
11	26526328	26526359	31	DEL	AC9	ANO3	NM_001313726	intron5-intron5
11	26601639	26601967	328	INS	AC2	ANO3	NM_001313726	intron15-intron15
11	22262800	22262928	128	INS	AC6	ANO5	NM_001142649	intron9-intron9
4	80841015	80841053	38	DEL	AC13	ANTXR2	NM_001286781	intron16-intron16
4	80888049	80894100	6051	DEL	AC2,AC6,AC9	ANTXR2	NM_001286781	intron16-intron16
21	27374157	27374701	544	INV	AC2,AC9	APP	NM_001136016	intron6-intron6
20	47576001	47576343	342	INS	AC2	ARFGEF2	NM_006420	intron6-intron6
1	1465919	1466238	319	DEL	AC9	ATAD3A	NM_001170535	intron15-intron15
7	138421854	138421891	37	INS	AC13	ATP6V0A4	NM_130841	intron14-intron14
7	33371749	33371803	54	DEL	AC6	BBS9	NM_001348036	intron9-intron9
7	33403540	33403868	328	DEL	AC2	BBS9	NM_001348036	intron16-intron16
18	60885837	60886060	223	DUP	AC13	BCL2	NM_000633	intron2-intron2
18	60932136	60932247	111	INS	AC2,AC6,AC12	BCL2	NM_000633	intron2-intron2
7	34087396	34087535	139	INS	AC2,AC12	BMPER	NM_133468	intron8-intron8
4	95870456	95870773	317	DEL	AC2	BMPR1B	NM_001203	intron2-intron2
7	134350003	134350427	424	DEL	AC2,AC12,AC13	BPGM	NM_001293085	intron3-intron3
6	105567843	105568181	338	DEL	AC2,AC12,AC13	BVES	NM_147147	intron5-intron5

11	73777314	73777365	51	INS	AC2	C2CD3	NM_001286577	intron24-intron24
12	2364958	2365258	300	DUP	AC9,AC12,AC13	CACNA1C	NM_000719	intron3-intron3
12	2629236	2630059	823	DUP	AC9	CACNA1C	NM_000719	intron9-intron9
12	2648367	2649065	698	DUP	AC9	CACNA1C	NM_000719	intron9-intron9
17	48665719	48665834	115	INS	AC6,AC12	CACNA1G	NM_001256324	intron9-intron9
10	18731849	18731938	89	DEL	AC6	CACNB2	NM_001167945	intron3-intron3
1	7517090	7517126	36	DEL	AC6	CAMTA1	NM_001349608	intron4-intron4
1	7602626	7602688	62	DEL	AC13	CAMTA1	NM_001349608	intron5-intron5
6	17472278	17472591	313	DEL	AC2	CAP2	NM_001363533	intron4-intron4
18	57258189	57258527	338	DEL	AC2	CCBE1	NM_133459	intron2-intron2
10	73180220	73180686	466	INS	AC9	CDH23	NM_052836	intron1-intron1
9	123257031	123259737	2706	DEL	AC13	CDK5RAP2	NM_001272039	intron12-intron12
4	85520548	85520584	36	DEL	AC2,AC6,AC9	CDS1	NM_001263	intron1-intron1
5	122723705	122723763	58	DEL	AC2,AC13	CEP120	NM_001166226	intron9-intron9
12	88520871	88520943	72	INS	AC2,AC12,AC13	CEP290	NM_025114	intron11-intron11
15	93515792	93516106	314	DEL	AC2,AC6	CHD2	NM_001271	intron19-intron19
20	61982526	61982607	81	INS	AC6	CHRNA4	NM_001256573	intron4-intron4
12	120201093	120201207	114	INS	AC9	CIT	NM_001206999	intron19-intron19
1	16376516	16376597	81	INS	AC2,AC6,AC9	CLCNKB	NM_000085	intron10-intron10
3	190119027	190119095	68	DEL	AC6,AC12,AC13	CLDN16	NM_006580	intron1-intron1
16	57992228	57992270	42	INS	AC2	CNGB1	NM_001286130	intron11-intron11
8	87595253	87595287	34	INS	AC2,AC13	CNGB3	NM_019098	intron15-intron15
7	147879436	147879471	35	DEL	AC13	CNTNAP2	NM_014141	intron17-intron17
7	148072862	148076322	3460	DEL	AC2	CNTNAP2	NM_014141	intron20-intron20
7	107059074	107063156	4082	INV	AC2	COG5	NM_006348	intron6-intron6
10	105795612	105795652	40	INS	AC9	COL17A1	NM_000494	intron48-intron48
10	105817204	105817411	207	INS	AC2,AC9,AC13	COL17A1	NM_000494	intron16-intron16
21	46927370	46927413	43	DEL	AC2	COL18A1	NM_130445	intron38-intron38
9	117040669	117041002	333	INS	AC2	COL27A1	NM_032888	intron37-intron37
2	189845085	189845161	76	DEL	AC9	COL3A1	NM_000090	intron1-intron1
13	111076803	111077141	338	INS	AC2	COL4A2	NM_001846	intron4-exon5
9	137548737	137549516	779	DUP	AC13	COL5A1	NM_000093	intron1-intron1
9	137690414	137690499	85	DEL	AC2,AC9	COL5A1	NM_000093	intron37-intron37
2	238252156	238252195	39	DEL	AC13	COL6A3	NM_057167	intron35-intron35
2	3682672	3682712	40	DEL	AC13	COLEC11	NM_001255983	intron3-intron3
19	18800291	18800806	515	INS	AC2	CRTC1	NM_001098482	intron1-intron1
18	77492396	77492433	37	DEL	AC13	CTDP1	NM_004715	intron11-intron11
10	67831141	67831190	49	INS	AC12,AC13	CTNNA3	NM_001127384	intron14-intron14
10	68049103	68049356	253	INS	AC2	CTNNA3	NM_001127384	intron12-intron12
16	88779323	88779686	363	DUP	AC13	CTU2	NM_001012759	intron7-intron7
1	58343214	58343539	325	DEL	AC6,AC9	DAB1	NM_021080	intron2-intron2
1	100703691	100703735	44	DEL	AC9	DBT	NM_001918	intron2-intron2
18	50732496	50732582	86	INS	AC13	DCC	NM_005215	intron10-intron10
18	50834260	50834297	37	DEL	AC2	DCC	NM_005215	intron13-intron13
6	24325362	24327809	2447	DEL	AC2	DCDC2	NM_016356	intron2-intron2
12	125433332	125433414	82	DEL	AC6	DHX37	NM_032656	intron25-intron25
13	60611165	60611298	133	INS	AC6	DIAPH3/DIAPH3-AS1	NM_001042517	intron5-intron5
2	233111614	233111749	135	INS	AC2,AC6	DIS3L2	NM_152383	intron11-intron11
8	13364088	13364175	87	INS	AC13	DLC1	NM_182643	intron1-intron1

X	31546267	31546354	87	DEL	AC9	DMD	NM_004021	intron12-intron12
X	31577470	31577784	314	DEL	AC13	DMD	NM_004021	intron12-intron12
X	33208587	33208912	325	DEL	AC9	DMD	NM_004006	intron1-intron1
19	55671142	55671178	36	DEL	AC6	DNAAF3	NM_001256714	intron10-intron10
17	76461583	76461623	40	DEL	AC2	DNAH17	NM_173628	intron56-intron56
17	76505128	76505311	183	DEL	AC6	DNAH17	NM_173628	intron27-intron27
5	13895083	13895384	301	DEL	AC2,AC6,AC13	DNAH5	NM_001369	intron15-intron15
7	157149904	157149978	74	INS	AC13	DNAJB6	NM_005494	intron1-intron1
10	69585411	69585504	93	INS	AC6	DNAJC12	NM_021800	intron1-intron1
1	65767936	65767976	40	DEL	AC13	DNAJC6	NM_001256865	intron1-intron1
1	63151823	63152164	341	DEL	AC9,AC12	DOCK7	NM_001271999	intron1-intron1
4	3467915	3474537	6622	DUP	AC9	DOK7	NM_001164673	intron2-intron2
7	154128521	154128566	45	DEL	AC9,AC13	DPP6	NM_001364497	intron2-intron2
7	154153816	154155078	1262	DEL	AC2,AC13	DPP6	NM_001364497	intron3-intron3
7	154446115	154446170	55	INS	AC2	DPP6	NM_001364497	intron7-intron7
7	154459913	154460041	128	INS	AC2	DPP6	NM_001364497	intron7-intron7
7	154586522	154586580	58	INS	AC6,AC9,AC13	DPP6	NM_001364497	intron12-intron12
7	154594052	154594088	36	DEL	AC13	DPP6	NM_001364497	intron14-intron14
1	97599554	97599605	51	DEL	AC2,AC13	DPYD/DPYD-AS1	NM_000110	intron20-intron20
6	116750824	116751143	319	DEL	AC9	DSE	NM_001374522	intron4-intron4
18	46593458	46593900	442	DUP	AC9	DYM	NM_001353210	intron16-intron16
11	103087932	103088089	157	DEL	AC6,AC13	DYNC2H1	NM_001080463	intron55-intron55
X	68865430	68865515	85	INS	AC6,AC9	EDA	NM_001005610	intron1-intron1
4	148458956	148458999	43	DEL	AC13	EDNRA	NM_001166055	intron3-intron3
11	85983113	85984033	920	INS	AC2	EED	NM_001308007	intron10-intron10
1	231511056	231511088	32	DEL	AC2	EGLN1	NM_001377260	intron1-intron1
9	140563768	140564031	263	DUP	AC13	EHMT1	NM_001354611	intron1-intron1
2	212551251	212551549	298	DEL	AC9,AC13	ERBB4	NM_001042599	intron12-intron12
2	212879767	212879810	43	DEL	AC13	ERBB4	NM_001042599	intron2-intron2
2	212925311	212925645	334	INS	AC2	ERBB4	NM_001042599	intron2-intron2
2	213195781	213195883	102	INS	AC9	ERBB4	NM_001042599	intron1-intron1
8	27662510	27662840	330	DEL	AC2,AC13	ESCO2	NM_001017420	exon11-txEnd
15	76522854	76522942	88	INS	AC2,AC6,AC9	ETFA	NM_000126	intron10-intron10
19	51860637	51860981	344	INS	AC2	ETFB	NM_001985	intron1-intron1
12	12026415	12027153	738	DEL	AC2	ETV6	NM_001987	intron5-intron5
6	65152734	65152780	46	DEL	AC9,AC12	EYS	NM_001292009	intron26-intron26
6	65185158	65185472	314	DEL	AC2,AC9	EYS	NM_001292009	intron26-intron26
6	65342505	65342555	50	INS	AC13	EYS	NM_001292009	intron22-intron22
6	66163974	66164293	319	INS	AC2	EYS	NM_001292009	intron5-intron5
6	66260031	66262026	1995	DEL	AC2,AC6,AC12	EYS	NM_001292009	intron2-intron2
6	66274848	66275174	326	DEL	AC13	EYS	NM_001292009	intron2-intron2
6	66399046	66404687	5641	DEL	AC2	EYS	NM_001292009	intron1-intron1
13	113790943	113791271	328	DEL	AC2,AC6,AC9	F10	NM_000504	intron2-intron2
6	6277345	6277612	267	DEL	AC2,AC13	F13A1	NM_000129	intron3-intron3
1	197011863	197012161	298	INS	AC9	F13B	NM_001994	intron10-intron10
1	169524857	169525497	640	INS	AC9	F5	NM_000130	intron6-intron6
7	205219	205396	177	DUP	AC6	FAM20C	NM_020223	intron2-intron2
5	150914533	150914571	38	INS	AC9	FAT2	NM_001447	intron11-intron11
3	192063188	192063501	313	DEL	AC6,AC12,AC13	FGF12	NM_001377294	intron2-intron2

3	192358911	192359209	298	DEL	AC9,AC13	FGF12	NM_001377292	intron2-intron2
13	102813915	102814035	120	DEL	AC2	FGF14	NM_001321938	intron3-intron3
13	102886533	102886724	191	DEL	AC2	FGF14	NM_001321938	intron2-intron2
1	213039231	213039404	173	DEL	AC2,AC13	FLVCR1	NM_014053	intron2-intron2
1	240348222	240348519	297	DUP	AC2	FMN2	NM_001305424	intron4-intron4
3	71242353	71242653	300	DEL	AC2	FOXP1	NM_001244814	intron2-intron2
3	71376467	71376719	252	INS	AC13	FOXP1	NM_001244808	intron4-intron4
7	113953513	113953825	312	DEL	AC2	FOXP2	NR_033766	intron2-intron2
1	74755168	74755289	121	DEL	AC2,AC9	FPGT-TNNI3K/TNNI3K	NM_015978	intron5-intron5
4	79159508	79159856	348	INS	AC12	FRAS1	NM_025074	intron3-intron3
2	49354881	49354956	75	DEL	AC9	FSHR	NM_000145	intron1-intron1
9	71665331	71665566	235	DEL	AC2,AC6,AC9	FXN	NM_000144	intron2-intron2
9	101209337	101209650	313	DEL	AC2	GABBR2	NM_005458	intron7-intron7
9	101309046	101311668	2622	DEL	AC13	GABBR2	NM_005458	intron2-intron2
8	75375342	75375684	342	INS	AC13	GDAP1	NM_001362931	intron5-intron5
9	6629936	6629982	46	INS	AC2	GLDC	NM_000170	intron2-intron2
2	121680862	121680901	39	DEL	AC2,AC6	GLI2	NM_001371271	intron2-intron2
9	3953763	3953803	40	DEL	AC12	GLIS3	NM_001042413	intron4-intron4
18	11874564	11875042	478	INS	AC2	GNAL	NM_182978	intron10-intron10
12	102141412	102142068	656	INS	AC2	GNPTAB	NM_024312	intron20-intron20
12	65134835	65135012	177	DEL	AC9,AC13	GNS	NM_002076	intron6-intron6
13	95044890	95045027	137	DEL	AC13	GPC6	NM_005708	intron7-intron7
3	32153721	32153878	157	DEL	AC6	GPD1L	NM_015141	intron1-intron1
14	67012203	67012301	98	DEL	AC13	GPHN	NM_001024218	intron1-intron1
8	144295476	144295532	56	DEL	AC13	GPIHBP1	NM_001301772	intron1-intron1
1	240675801	240675984	183	INS	AC2,AC13	GREM2	NM_022469	intron1-intron1
1	240708772	240708808	36	DEL	AC6	GREM2	NM_022469	intron1-intron1
4	93381184	93381225	41	INS	AC13	GRID2	NM_001510	intron1-intron1
4	93393072	93393391	319	DEL	AC2	GRID2	NM_001510	intron1-intron1
4	93499194	93499254	60	DEL	AC6,AC9	GRID2	NM_001510	intron1-intron1
4	93567357	93570164	2807	DUP	AC12,AC13	GRID2	NM_001510	intron2-intron2
4	93579941	93580270	329	DEL	AC9	GRID2	NM_001510	intron2-intron2
4	94619025	94619065	40	INS	AC2	GRID2	NM_001510	intron14-intron14
4	94662315	94662348	33	DEL	AC2	GRID2	NM_001510	intron14-intron14
6	102172004	102172347	343	INS	AC13	GRIK2	NM_001166247	intron6-intron6
12	13941609	13941706	97	DEL	AC2	GRIN2B	NM_000834	intron3-intron3
15	28430091	28430340	249	INS	AC2	HERC2	NM_004667	intron56-intron56
15	72639547	72639915	368	INS	AC9	HEXA	NM_000520	intron10-intron10
7	81358335	81358612	277	DEL	AC2,AC6,AC13	HGF	NM_000601	intron8-intron8
2	191179019	191179054	35	DEL	AC9	HIBCH	NM_014362	intron1-intron1
6	143161426	143161510	84	DEL	AC2,AC9,AC13	HIVEP2	NM_006734	intron1-intron1
12	122289595	122289938	343	DEL	AC13	HPD	NM_002150	intron7-intron7
10	100496979	100497287	308	DEL	AC13	HPSE2	NM_021828	intron4-intron4
9	99062579	99062918	339	DEL	AC2	HSD17B3	NM_000197	intron1-intron1
3	129184473	129184785	312	DEL	AC6,AC12	IFT122	NM_001280545	intron8-intron8
6	160521749	160521819	70	DEL	AC2,AC9	IGF2R	NM_000876	intron45-intron45
8	42185321	42185632	311	DEL	AC9	IKBKB	NM_001242778	intron19-intron19
10	6097375	6097877	502	DEL	AC2,AC6,AC9	IL2RA	NM_000417	intron1-intron1
14	105186822	105186923	101	DEL	AC13	INF2	NM_001031714	exon22-exon22

15	40714963	40715150	187	INS	AC6	IVD	NM_001354598	intron11-intron11
16	87705207	87705258	51	DEL	AC2	JPH3	NR_073379	intron2-intron2
11	128785941	128786104	163	DEL	AC2	KCNJ5	NM_000890	intron2-intron2
21	39050079	39050152	73	INS	AC13	KCNJ6	NM_002240	intron3-intron3
21	39080016	39080521	505	INS	AC13	KCNJ6	NM_002240	intron3-intron3
21	39196237	39196334	97	INS	AC2,AC6,AC9,AC12	KCNJ6	NM_002240	intron2-intron2
21	39233185	39233234	49	DEL	AC2	KCNJ6	NM_002240	intron1-intron1
10	78800905	78801227	322	DEL	AC2,AC13	KCNMA1	NM_001322838	intron15-intron15
20	62081875	62081939	64	DUP	AC6	KCNQ2	NM_004518	intron1-intron1
16	27724967	27725198	231	INS	AC2	KIAA0556/LOC100128079	NM_015202	intron13-intron13
4	123188205	123188257	52	INS	AC2	KIAA1109	NM_015312	intron44-intron44
2	10184250	10184297	47	DEL	AC2,AC9,AC13	KLF11	NM_003597	intron1-intron1
5	137022565	137023897	1332	DEL	AC2,AC9,AC12	KLHL3	NM_001257195	intron3-intron3
12	53207592	53207637	45	INS	AC6	KRT4	NM_002272	exon1-exon1
18	21490977	21491121	144	DEL	AC6	LAMA3	NM_001127717	intron54-intron54
1	66055935	66056272	337	INS	AC12	LEPR	NM_001003680	intron5-intron5
1	66079149	66079422	273	DEL	AC6,AC13	LEPR	NM_001003680	intron14-intron14
1	66079459	66079518	59	INS	AC2,AC12	LEPR	NM_001003680	intron14-intron14
10	95545474	95546468	994	DEL	AC2,AC13	LGI1	NM_001308275	intron4-intron4
15	77910867	77911242	375	DEL	AC2	LINGO1	NM_001301199	intron3-intron3
15	77991616	77992116	500	DUP	AC9	LINGO1	NM_001301198	intron1-intron1
16	964490	964824	334	DUP	AC2	LMF1	NM_001352021	intron3-intron3
18	2997373	2997457	84	DEL	AC13	LPIN2	NM_014646	intron1-intron1
3	188304835	188305178	343	DEL	AC2,AC6,AC9	LPP	NM_001375455	intron5-intron5
3	188473595	188473635	40	DEL	AC9,AC13	LPP	NM_001375455	intron7-intron7
4	151875131	151875221	90	INS	AC6,AC13	LRBA	NM_001364905	intron2-intron2
10	78066263	78066336	73	DEL	AC6	LRMDA	NM_001305581	intron5-intron5
10	78095877	78095923	46	INS	AC6	LRMDA	NM_001305581	intron6-intron6
11	68183094	68183412	318	DEL	AC2,AC6,AC13	LRP5	NM_001291902	intron12-intron12
9	130226648	130226703	55	DEL	AC2	LRSAM1	NM_001005373	intron9-intron9
22	21343986	21344023	37	DEL	AC13	LZTR1	NM_006767	intron7-intron7
7	1927503	1927634	131	INS	AC9	MAD1L1	NM_001304525	intron3-intron3
7	2042682	2042744	62	INS	AC2,AC6	MAD1L1	NM_001013836	intron13-intron13
7	2200745	2200840	95	INS	AC6	MAD1L1	NM_001013836	intron10-intron10
11	47321173	47321229	56	INS	AC2	MADD	NM_001376651	intron22-intron22
7	77863868	77863950	82	DEL	AC2	MAGI2	NM_001301128	intron10-intron10
7	78357850	78358190	340	DEL	AC9	MAGI2	NM_001301128	intron2-intron2
7	78606783	78606824	41	DEL	AC6	MAGI2	NM_001301128	intron2-intron2
11	96001924	96003394	1470	DEL	AC2	MAML2	NM_032427	intron1-intron1
6	91237581	91237902	321	DEL	AC9	MAP3K7	NM_003188	intron12-intron12
18	32661965	32662283	318	DEL	AC6	MAPRE2	NM_001143826	intron3-intron3
6	119164813	119164861	48	INS	AC12,AC13	MCM9	NM_017696	intron7-intron7
12	116715140	116715261	121	INS	AC6	MED13L	NM_015335	exon1-exon1
5	88032019	88032354	335	DEL	AC2,AC6,AC9,AC13	MEF2C	NM_001364353	intron4-intron4
5	88043148	88043474	326	DEL	AC2	MEF2C	NM_001364353	intron4-intron4
22	42140481	42140804	323	DEL	AC6,AC12	MEI1	NM_152513	intron12-intron12
3	70013474	70013887	413	DEL	AC12	MITF	NM_001354604	intron9-intron9
21	33672992	33673079	87	INS	AC2,AC6	MRAP	NM_001285394	intron2-intron2
5	80039370	80039681	311	DEL	AC9	MSH3	NM_002439	intron11-intron11

8	15997256	15997314	58	DEL	AC9,AC13	MSR1	NM_138716	intron8-intron8
9	113463930	113464147	217	DEL	AC12	MUSK	NM_001166280	intron5-intron5
12	102030825	102030909	84	INS	AC2,AC9,AC13	MYBPC1	NM_001254718	intron8-intron8
16	15869266	15869316	50	DEL	AC9	MYH11	NM_001040113	intron9-intron9
18	47694973	47698387	3414	DEL	AC12	MYO5B	NM_001080467	intron1-intron1
2	1865365	1865769	404	DUP	AC2,AC9,AC13	MYT1L	NM_001329845	intron18-intron18
2	2239127	2239195	68	DEL	AC13	MYT1L	NM_001329845	intron2-intron2
2	2266692	2266749	57	INS	AC2	MYT1L	NM_001329845	intron2-intron2
4	140289133	140289177	44	DEL	AC6	NAA15	NM_057175	intron14-intron14
13	101894143	101896424	2281	DEL	AC12	NALCN	NM_001350748	intron11-intron11
2	240958945	240959270	325	DEL	AC2,AC9	NDUFA10	NR_136158	intron3-intron3
18	55801583	55801911	328	DEL	AC2,AC6,AC9	NEDD4L	NM_001144967	intron1-intron1
17	29662295	29662409	114	INS	AC2,AC9	NF1	NM_000267	intron39-intron39
2	69650285	69650336	51	DEL	AC12	NFU1	NM_015700	intron3-intron3
3	25802351	25802392	41	DEL	AC2,AC9,AC13	NGLY1	NM_018297	intron3-intron3
3	25824977	25825022	45	DEL	AC9	NGLY1	NM_001145294	intron1-intron1
X	17502751	17502820	69	DEL	AC6	NHS	NM_001291867	intron1-intron1
17	5464742	5465066	324	DEL	AC6	NLRP1	NM_001033053	intron3-intron3
9	139422831	139423032	201	DUP	AC2,AC13	NOTCH1	NM_017617	intron2-intron2
19	15284535	15284571	36	DEL	AC2	NOTCH3	NM_000435	intron25-intron25
2	50602635	50602974	339	INS	AC13	NRXN1	NM_001330078	intron17-intron17
8	126336808	126337124	316	DEL	AC2	NSMCE2	NM_001349485	intron4-intron4
1	156806529	156806581	52	DEL	AC2	NTRK1	NM_001007792	intron1-intron1
9	87345362	87345678	316	DEL	AC13	NTRK2	NM_001007097	intron11-intron11
17	801621	802218	597	DEL	AC2	NXN	NM_022463	intron1-intron1
15	28175796	28176205	409	INS	AC2	OCA2	NM_000275	intron18-intron18
11	132845424	132845456	32	DEL	AC2	OPCML	NM_001012393	intron1-intron1
8	107417374	107417420	46	DEL	AC9	OXR1	NM_001198533	intron2-intron2
8	107511104	107511428	324	DEL	AC2	OXR1	NM_001198533	intron2-intron2
8	107728565	107728894	329	DEL	AC13	OXR1	NM_001198533	intron11-intron11
11	93871030	93871132	102	INS	AC6	PANX1	NM_015368	intron1-intron1
1	19070924	19071004	80	INS	AC13	PAX7	NM_001135254	intron8-intron8
1	164845210	164845255	45	DEL	AC12	PBX1	NM_001353131	intron7-intron7
3	136021010	136026196	5186	DEL	AC2	PCCB	NM_001178014	intron10-intron10
10	55893677	55894125	448	DEL	AC6	PCDH15	NM_001142771	intron15-intron15
10	56762357	56762424	67	INS	AC2	PCDH15	NM_001354404	intron3-intron3
5	58327196	58327231	35	DEL	AC6	PDE4D	NM_001197221	intron2-intron2
10	95414513	95414548	35	DEL	AC9	PDE6C	NM_006204	intron15-intron15
17	79619832	79619886	54	DEL	AC6,AC13	PDE6G	NM_001365725	intron2-intron2
12	7355778	7356081	303	INS	AC13	PEX5	NM_001351124	intron8-exon9
1	64112928	64112963	35	DEL	AC9	PGM1	NM_002633	intron7-intron7
11	46036244	46036590	346	DEL	AC2	PHF21A	NM_001352028	intron6-intron6
18	10757445	10757525	80	DEL	AC2,AC12	PIEZO2	NM_022068	intron25-intron25
18	10915932	10915973	41	DEL	AC6	PIEZO2	NM_022068	intron3-intron3
18	59728660	59728768	108	DEL	AC2	PIGN	NM_012327	intron29-intron29
18	59834525	59834560	35	DEL	AC13	PIGN	NM_012327	intron1-intron1
11	17195845	17196157	312	DEL	AC13	PIK3C2A	NM_001321378	intron2-intron2
17	6422505	6422670	165	DEL	AC9	PITPNM3	NM_001165966	intron2-intron2
7	47895181	47895508	327	DEL	AC2	PKD1L1	NM_138295	intron28-intron28

6	51517356	51517682	326	DEL	AC2,AC13	PKHD1	NM_138694	intron61-intron61
6	51739569	51745614	6045	DEL	AC9,AC13	PKHD1	NM_138694	intron46-intron46
12	32963605	32963921	316	DEL	AC2,AC6,AC13	PKP2	NM_004572	intron10-intron10
20	9389098	9389164	66	INS	AC9	PLCB4	NM_001377134	intron21-intron21
12	80239678	80239721	43	INS	AC9	PPP1R12A	NM_001143886	intron2-intron2
5	146026781	146026825	44	INS	AC2,AC6,AC13	PPP2R2B	NR_073526	intron5-intron5
5	146394710	146397305	2595	DEL	AC9	PPP2R2B	NM_001271900	intron2-intron2
5	146396114	146396176	62	INS	AC6	PPP2R2B	NM_001271900	intron2-intron2
5	146396115	146396177	62	INS	AC6	PPP2R2B	NM_001271900	intron2-intron2
4	121808935	121809250	315	DEL	AC9	PRDM5	NM_001300823	intron2-intron2
5	122431837	122432027	190	INS	AC9	PRDM6	NM_001136239	intron2-intron2
12	42871291	42871330	39	DEL	AC2,AC9	PRICKLE1	NM_001144881	intron1-intron1
10	53461462	53461501	39	DEL	AC13	PRKG1	NM_001098512	intron3-intron3
10	53947968	53948034	66	DEL	AC13	PRKG1	NM_001098512	intron9-intron9
6	161795626	161795801	175	INS	AC2,AC13	PRKN	NM_004562	intron10-intron10
6	161973423	161973482	59	DEL	AC2,AC9	PRKN	NM_004562	intron8-intron8
1	214576009	214576061	52	INS	AC9,AC13	PTPN14	NM_005401	intron6-intron6
11	48120066	48120374	308	DEL	AC9	PTPRJ	NM_001098503	intron1-intron1
2	1723678	1724041	363	DEL	AC9	PXDN	NM_012293	intron1-intron1
14	51408899	51409530	631	DEL	AC13	PYGL	NM_001163940	intron1-intron1
11	64526480	64526561	81	INS	AC2,AC6	PYGM	NM_001164716	intron1-intron1
3	25030886	25032242	1356	DEL	AC2,AC13	RARB	NM_001290216	intron2-intron2
3	25508991	25509031	40	DEL	AC9	RARB	NM_001290216	intron5-intron5
8	53611229	53611262	33	DEL	AC2,AC9	RB1CC1	NM_014781	intron1-intron1
7	103463073	103463395	322	DEL	AC2,AC9	RELN	NM_173054	intron3-intron3
1	8729019	8729097	78	DEL	AC6,AC9	RERE	NM_001042681	intron1-intron1
6	151771280	151771319	39	DEL	AC2	RMND1	NM_017909	intron1-intron1
3	149593684	149593794	110	INS	AC9,AC13	RNF13	NM_007282	intron5-intron5
4	1086772	1086826	54	DEL	AC2,AC9	RNF212	NM_001366918	intron3-intron3
14	21778182	21778401	219	DEL	AC2,AC13	RPGRIP1	NM_020366	intron6-intron6
16	53713453	53713556	103	DEL	AC13	RPGRIP1L	NM_001127897	intron6-intron6
2	89001136	89001221	85	INS	AC2,AC9,AC13	RPIA	NM_144563	intron3-intron3
11	14348317	14348634	317	DEL	AC6	RRAS2	NM_001177315	intron1-intron1
1	38077349	38077420	71	DEL	AC2,AC6,AC9	RSPO1	NM_001038633	intron8-intron8
16	57231835	57231879	44	DEL	AC2	RSPRY1	NM_001305182	intron1-intron1
16	57252627	57252950	323	DEL	AC2	RSPRY1	NM_001305163	intron8-intron8
1	237449856	237450067	211	INS	AC9,AC13	RYR2	NM_001035	intron2-intron2
1	237679029	237679180	151	INS	AC6	RYR2	NM_001035	intron24-intron24
8	119165528	119165600	72	DEL	AC2	SAMD12	NR_146234	intron4-intron4
8	119447054	119447364	310	DEL	AC6	SAMD12	NR_146234	intron3-intron3
8	119618005	119618050	45	DEL	AC2,AC6	SAMD12	NR_146234	intron1-intron1
3	39011493	39011530	37	DEL	AC12	SCN11A	NM_001349253	intron2-intron2
6	108266118	108266421	303	DEL	AC9	SEC63	NM_007214	intron1-intron1
6	158548271	158549075	804	DEL	AC2,AC6,AC9,AC13	SERAC1	NM_032861	intron10-intron10
17	1654662	1655386	724	DEL	AC13	SERPINF2	NM_000934	intron8-intron8
5	155777256	155777300	44	INS	AC2,AC6	SGCD	NM_000337	intron3-intron3
5	148401441	148401598	157	DEL	AC9	SH3TC2	NM_024577	intron12-intron12
15	48550270	48550592	322	DEL	AC13	SLC12A1	NM_000338	intron16-intron16
5	36649430	36649465	35	DEL	AC13	SLC1A3	NM_001289939	intron2-intron2

14	92941046	92941091	45	DEL	AC2,AC6	SLC24A4	NM_153648	intron12-intron12
3	45831052	45831107	55	DEL	AC6,AC13	SLC6A20	NM_020208	intron1-intron1
5	492290	492351	61	DEL	AC13	SLC9A3	NM_001284351	intron1-intron1
18	2748477	2748513	36	DEL	AC2,AC9	SMCHD1	NM_015295	intron30-intron30
15	25621911	25621955	44	INS	AC2	SNHG14/UBE3A	NM_001354506	intron6-intron6
2	39306291	39306358	67	INS	AC13	SOS1	NM_005633	intron1-intron1
12	24460447	24460731	284	DEL	AC13	SOX5	NM_001261414	intron3-intron3
14	88890014	88890172	158	DEL	AC6,AC13	SPATA7	NM_001040428	intron4-intron4
22	24712801	24713504	703	DEL	AC9	SPECC1L/SPECC1L-ADORA2A	NM_001145468	intron3-intron3
5	35752763	35752928	165	INS	AC2	SPEF2	NM_024867	intron23-intron23
2	220321615	220321683	68	DEL	AC2,AC6,AC13	SPEG	NM_005876	intron6-intron6
5	147462158	147462476	318	DEL	AC9,AC12,AC13	SPINK5	NM_001127699	intron4-intron4
14	78036158	78036303	145	INS	AC6,AC13	SPTLC2	NM_004863	intron5-intron5
14	35476847	35476880	33	DEL	AC9	SRP54	NM_001146282	intron4-intron4
3	133503776	133505093	1317	DEL	AC9	SRPRB/TF	NM_001354703	exon23-exon23
2	96862806	96862859	53	INS	AC2	STARD7	NM_020151	intron1-intron1
19	7708892	7708961	69	INS	AC13	STXBP2	NM_001127396	intron13-intron13
17	80732491	80732528	37	DEL	AC9	TBCD	NM_005993	intron6-intron6
17	80856030	80856094	64	DEL	AC6	TBCD	NM_005993	intron17-intron17
4	107056577	107063362	6785	DEL	AC13	TBCK	NM_001163435	intron23-intron23
11	12943735	12944014	279	DEL	AC2	TEAD1	NM_021961	intron10-intron10
11	78516321	78516389	68	INS	AC2	TENM4	NM_001098816	exon15-intron15
3	24306049	24306093	44	DEL	AC9,AC13	THRB	NM_000461	intron2-intron2
9	71738120	71743354	5234	DEL	AC2	TJP2	NM_001170414	intron1-intron1
9	71777933	71778252	319	DEL	AC9,AC12	TJP2	NM_001170414	intron2-intron2
11	61107790	61108130	340	DEL	AC6	TKFC	NM_001351978	intron5-intron5
3	53269956	53270027	71	INS	AC2	TKT	NM_001135055	intron4-intron4
4	166987132	166987267	135	INS	AC9	TLL1	NM_012464	intron16-intron16
4	167004312	167004908	596	DEL	AC2	TLL1	NM_012464	intron18-intron18
1	165731218	165731496	278	DEL	AC13	TMCO1	NM_019026	intron2-intron2
7	12281703	12282030	327	DEL	AC6,AC12,AC13	TMEM106B	NM_001134232	exon8-exon8
3	189363420	189370899	7479	DEL	AC2	TP63	NM_001329964	intron1-intron1
3	189575904	189576229	325	DEL	AC2,AC9	TP63	NM_001329964	intron4-intron4
7	144382754	144382903	149	DEL	AC2,AC9,AC12,AC13	TPK1	NM_001350884	intron1-intron1
8	140884957	140885165	208	DEL	AC2,AC6,AC13	TRAPPC9	NM_001160372	intron21-intron21
8	141361276	141361615	339	INS	AC2	TRAPPC9	NM_001160372	intron9-intron9
6	123924305	123924403	98	INS	AC2	TRDN	NM_006073	intron1-intron1
4	154095270	154095370	100	INS	AC13	TRIM2	NM_001375517	intron1-intron1
5	14347118	14347525	407	DUP	AC9	TRIO	NM_007118	intron11-intron11
8	116488226	116488556	330	DEL	AC2,AC6,AC13	TRPS1	NM_001282902	intron4-intron4
7	98550659	98550695	36	DEL	AC2,AC12,AC13	TRRAP	NM_001244580	intron38-intron38
14	81469121	81469435	314	DEL	AC6,AC9,AC13	TSHR	NM_001018036	intron1-intron1
14	81597047	81597366	319	DEL	AC13	TSHR	NM_000369	intron8-intron8
2	166787240	166787335	95	INS	AC13	TTC21B	NM_024753	intron8-intron8
4	147730176	147731088	912	DEL	AC13	TTC29	NM_001300761	intron11-intron11
2	179621885	179621984	99	INS	AC13	TTN	NM_001256850	intron44-intron44
2	210821023	210821250	227	INS	AC6,AC9	UNC80	NM_001371986	intron48-intron48
12	109544696	109544852	156	INS	AC2	UNG	NM_080911	intron6-intron6
11	17548693	17548744	51	DEL	AC6,AC9	USH1C	NM_001297764	intron5-intron5

1	216085794	216085857	63	DEL	AC9,AC12	USH2A	NM_206933	intron38-intron38
1	216185310	216185482	172	DEL	AC2,AC12	USH2A	NM_206933	intron32-intron32
X	41057618	41057683	65	DEL	AC6	USP9X	NM_001039590	intron29-intron29
4	1366458	1366690	232	DUP	AC2	UVSSA	NM_001317934	intron8-intron8
12	6574523	6574574	51	DEL	AC6	VAMP1	NM_001297438	intron3-intron3
2	20185026	20185288	262	DUP	AC13	WDR35	NM_001006657	intron2-intron2
16	78178327	78178402	75	INS	AC2,AC9	WWOX	NM_130791	intron4-intron4
16	78227441	78227868	427	INS	AC13	WWOX	NM_130791	intron5-intron5
3	14196291	14196373	82	DEL	AC2	XPC	NM_001354727	intron9-intron9
1	180828198	180828253	55	INS	AC2,AC6,AC13	XPR1	NM_001135669	intron10-intron10
5	82482756	82483062	306	DEL	AC2	XRCC4	NM_022550	intron3-intron3
3	114466479	114466804	325	DEL	AC2,AC9	ZBTB20	NM_001164343	intron4-intron4
2	145255390	145255441	51	DEL	AC9	ZEB2	NM_001171653	intron2-intron2
8	106670527	106670846	319	DEL	AC13	ZFPM2	NM_001362836	intron4-intron4
10	80925712	80925773	61	INS	AC2,AC13	ZMIZ1	NM_020338	intron4-intron4
16	88478396	88478501	105	INS	AC2,AC6,AC9	ZNF469	NM_001367624	intron1-intron1

Supplementary Table G: 397 class-4 SVs and 320 genes found by 4 tools in 5 unaffected relatives and by at least one tool in 10 probands

Chromosome	Start	End	Length	Type	Sample	Gene	Transcript	Location
15	67534473	67534808	335	DEL	AC6,AC9,AC13	AAGAB	NM_024666	intron1-intron1
10	101587739	101587960	221	INS	AC13	ABCC2	NM_000392	intron19-intron19
12	22016216	22016263	47	DEL	AC9,AC13	ABCC9	NM_020297	intron17-intron17
12	22047472	22047576	104	DEL	AC6,AC9	ABCC9	NM_020297	intron11-intron11
3	43735558	43735609	51	INS	AC6	ABHD5	NM_001365650	intron1-intron1
2	74128660	74128710	50	DEL	AC9	ACTG2	NM_001199893	intron2-intron2
1	236876894	236878365	1471	DUP	AC13	ACTN2	NM_001103	intron1-intron1
2	158719092	158719404	312	DEL	AC6,AC13	ACVR1	NM_001105	intron1-intron1
22	17665794	17665850	56	DEL	AC13	ADA2	NM_177405	intron4-intron4
15	58912853	58913174	321	DEL	AC2,AC6,AC13	ADAM10	NM_001110	intron11-intron11
8	38902903	38903082	179	INS	AC9,AC13	ADAM9	NM_003816	intron12-intron12
15	100606982	100607180	198	DEL	AC6	ADAMTS17	NM_139057	intron15-intron15
15	100693158	100693199	41	INS	AC2	ADAMTS17	NM_139057	intron9-intron9
4	73418754	73418842	88	DEL	AC2	ADAMTS3	NM_014243	intron2-intron2
9	136414108	136414435	327	DEL	AC9,AC13	ADAMTSL2	NM_001145320	intron9-intron9
5	89860428	89860749	321	DEL	AC6,AC9	ADGRV1	NM_032119	intron1-intron1
5	89997830	89998001	171	INS	AC13	ADGRV1	NM_032119	intron34-intron34
10	76230336	76230486	150	INS	AC6	ADK	NM_001202450	intron6-intron6
15	86805310	86805346	36	INS	AC12	AGBL1	NM_152336	intron9-intron9
2	178287637	178288051	414	DEL	AC6	AGPS	NM_003659	intron2-intron2
2	178315692	178315763	71	INS	AC13	AGPS	NM_003659	intron8-intron8
15	101453076	101453384	308	DEL	AC13	ALDH1A3/LOC101927751	NM_000693	intron12-intron12
15	85397465	85397801	336	INS	AC2	ALPK3	NM_020778	intron5-intron5
4	113985883	113986344	461	DEL	AC9	ANK2	NM_001354269	intron1-intron1
4	114018256	114018583	327	INS	AC12	ANK2	NM_001354269	intron1-intron1
4	114064436	114064510	74	INS	AC2,AC13	ANK2	NM_001354269	intron1-intron1
4	114135100	114135584	484	DEL	AC13	ANK2	NM_001354269	intron4-intron4
3	43565778	43565929	151	INS	AC2,AC13	ANO10	NM_018075	intron11-intron11
11	26526328	26526359	31	DEL	AC9	ANO3	NM_001313726	intron5-intron5
11	26601639	26601967	328	INS	AC2	ANO3	NM_001313726	intron15-intron15
11	22262800	22262928	128	INS	AC6	ANO5	NM_001142649	intron9-intron9
4	80841015	80841053	38	DEL	AC13	ANTXR2	NM_001286781	intron16-intron16
4	80888049	80894100	6051	DEL	AC2,AC6,AC9	ANTXR2	NM_001286781	intron16-intron16
21	27374157	27374701	544	INV	AC2,AC9	APP	NM_001136016	intron6-intron6
20	47576001	47576343	342	INS	AC2	ARFGEF2	NM_006420	intron6-intron6
1	1465919	1466238	319	DEL	AC9	ATAD3A	NM_001170535	intron15-intron15
7	138421854	138421891	37	INS	AC13	ATP6V0A4	NM_130841	intron14-intron14
7	33371749	33371803	54	DEL	AC6	BBS9	NM_001348036	intron9-intron9
7	33403540	33403868	328	DEL	AC2	BBS9	NM_001348036	intron16-intron16
18	60885837	60886060	223	DUP	AC13	BCL2	NM_000633	intron2-intron2
18	60932136	60932247	111	INS	AC2,AC6,AC12	BCL2	NM_000633	intron2-intron2
7	34087396	34087535	139	INS	AC2,AC12	BMPER	NM_133468	intron8-intron8
4	95870456	95870773	317	DEL	AC2	BMPR1B	NM_001203	intron2-intron2
7	134350003	134350427	424	DEL	AC2,AC12,AC13	BPGM	NM_001293085	intron3-intron3
6	105567843	105568181	338	DEL	AC2,AC12,AC13	BVES	NM_147147	intron5-intron5

11	73777314	73777365	51	INS	AC2	C2CD3	NM_001286577	intron24-intron24
12	2364958	2365258	300	DUP	AC9,AC12,AC13	CACNA1C	NM_000719	intron3-intron3
12	2629236	2630059	823	DUP	AC9	CACNA1C	NM_000719	intron9-intron9
12	2648367	2649065	698	DUP	AC9	CACNA1C	NM_000719	intron9-intron9
17	48665719	48665834	115	INS	AC6,AC12	CACNA1G	NM_001256324	intron9-intron9
10	18731849	18731938	89	DEL	AC6	CACNB2	NM_001167945	intron3-intron3
1	7517090	7517126	36	DEL	AC6	CAMTA1	NM_001349608	intron4-intron4
1	7602626	7602688	62	DEL	AC13	CAMTA1	NM_001349608	intron5-intron5
6	17472278	17472591	313	DEL	AC2	CAP2	NM_001363533	intron4-intron4
18	57258189	57258527	338	DEL	AC2	CCBE1	NM_133459	intron2-intron2
10	73180220	73180686	466	INS	AC9	CDH23	NM_052836	intron1-intron1
9	123257031	123259737	2706	DEL	AC13	CDK5RAP2	NM_001272039	intron12-intron12
4	85520548	85520584	36	DEL	AC2,AC6,AC9	CDS1	NM_001263	intron1-intron1
5	122723705	122723763	58	DEL	AC2,AC13	CEP120	NM_001166226	intron9-intron9
12	88520871	88520943	72	INS	AC2,AC12,AC13	CEP290	NM_025114	intron11-intron11
15	93515792	93516106	314	DEL	AC2,AC6	CHD2	NM_001271	intron19-intron19
20	61982526	61982607	81	INS	AC6	CHRNA4	NM_001256573	intron4-intron4
12	120201093	120201207	114	INS	AC9	CIT	NM_001206999	intron19-intron19
1	16376516	16376597	81	INS	AC2,AC6,AC9	CLCNKB	NM_000085	intron10-intron10
3	190119027	190119095	68	DEL	AC6,AC12,AC13	CLDN16	NM_006580	intron1-intron1
16	57992228	57992270	42	INS	AC2	CNGB1	NM_001286130	intron11-intron11
8	87595253	87595287	34	INS	AC2,AC13	CNGB3	NM_019098	intron15-intron15
7	147879436	147879471	35	DEL	AC13	CNTNAP2	NM_014141	intron17-intron17
7	148072862	148076322	3460	DEL	AC2	CNTNAP2	NM_014141	intron20-intron20
7	107059074	107063156	4082	INV	AC2	COG5	NM_006348	intron6-intron6
10	105795612	105795652	40	INS	AC9	COL17A1	NM_000494	intron48-intron48
10	105817204	105817411	207	INS	AC2,AC9,AC13	COL17A1	NM_000494	intron16-intron16
21	46927370	46927413	43	DEL	AC2	COL18A1	NM_130445	intron38-intron38
9	117040669	117041002	333	INS	AC2	COL27A1	NM_032888	intron37-intron37
2	189845085	189845161	76	DEL	AC9	COL3A1	NM_000090	intron1-intron1
13	111076803	111077141	338	INS	AC2	COL4A2	NM_001846	intron4-exon5
9	137548737	137549516	779	DUP	AC13	COL5A1	NM_000093	intron1-intron1
9	137690414	137690499	85	DEL	AC2,AC9	COL5A1	NM_000093	intron37-intron37
2	238252156	238252195	39	DEL	AC13	COL6A3	NM_057167	intron35-intron35
2	3682672	3682712	40	DEL	AC13	COLEC11	NM_001255983	intron3-intron3
19	18800291	18800806	515	INS	AC2	CRTC1	NM_001098482	intron1-intron1
18	77492396	77492433	37	DEL	AC13	CTDP1	NM_004715	intron11-intron11
10	67831141	67831190	49	INS	AC12,AC13	CTNNA3	NM_001127384	intron14-intron14
10	68049103	68049356	253	INS	AC2	CTNNA3	NM_001127384	intron12-intron12
16	88779323	88779686	363	DUP	AC13	CTU2	NM_001012759	intron7-intron7
1	58343214	58343539	325	DEL	AC6,AC9	DAB1	NM_021080	intron2-intron2
1	100703691	100703735	44	DEL	AC9	DBT	NM_001918	intron2-intron2
18	50732496	50732582	86	INS	AC13	DCC	NM_005215	intron10-intron10
18	50834260	50834297	37	DEL	AC2	DCC	NM_005215	intron13-intron13
6	24325362	24327809	2447	DEL	AC2	DCDC2	NM_016356	intron2-intron2
12	125433332	125433414	82	DEL	AC6	DHX37	NM_032656	intron25-intron25
13	60611165	60611298	133	INS	AC6	DIAPH3/DIAPH3-AS1	NM_001042517	intron5-intron5
2	233111614	233111749	135	INS	AC2,AC6	DIS3L2	NM_152383	intron11-intron11
8	13364088	13364175	87	INS	AC13	DLC1	NM_182643	intron1-intron1

X	31546267	31546354	87	DEL	AC9	DMD	NM_004021	intron12-intron12
X	31577470	31577784	314	DEL	AC13	DMD	NM_004021	intron12-intron12
X	33208587	33208912	325	DEL	AC9	DMD	NM_004006	intron1-intron1
19	55671142	55671178	36	DEL	AC6	DNAAF3	NM_001256714	intron10-intron10
17	76461583	76461623	40	DEL	AC2	DNAH17	NM_173628	intron56-intron56
17	76505128	76505311	183	DEL	AC6	DNAH17	NM_173628	intron27-intron27
5	13895083	13895384	301	DEL	AC2,AC6,AC13	DNAH5	NM_001369	intron15-intron15
7	157149904	157149978	74	INS	AC13	DNAJB6	NM_005494	intron1-intron1
10	69585411	69585504	93	INS	AC6	DNAJC12	NM_021800	intron1-intron1
1	65767936	65767976	40	DEL	AC13	DNAJC6	NM_001256865	intron1-intron1
1	63151823	63152164	341	DEL	AC9,AC12	DOCK7	NM_001271999	intron1-intron1
7	154128521	154128566	45	DEL	AC9,AC13	DPP6	NM_001364497	intron2-intron2
7	154153816	154155078	1262	DEL	AC2,AC13	DPP6	NM_001364497	intron3-intron3
7	154446115	154446170	55	INS	AC2	DPP6	NM_001364497	intron7-intron7
7	154459913	154460041	128	INS	AC2	DPP6	NM_001364497	intron7-intron7
7	154586522	154586580	58	INS	AC6,AC9,AC13	DPP6	NM_001364497	intron12-intron12
7	154594052	154594088	36	DEL	AC13	DPP6	NM_001364497	intron14-intron14
1	97599554	97599605	51	DEL	AC2,AC13	DPYD/DPYD-AS1	NM_000110	intron20-intron20
6	116750824	116751143	319	DEL	AC9	DSE	NM_001374522	intron4-intron4
18	46593458	46593900	442	DUP	AC9	DYM	NM_001353210	intron16-intron16
11	103087932	103088089	157	DEL	AC6,AC13	DYNC2H1	NM_001080463	intron55-intron55
X	68865430	68865515	85	INS	AC6,AC9	EDA	NM_001005610	intron1-intron1
4	148458956	148458999	43	DEL	AC13	EDNRA	NM_001166055	intron3-intron3
11	85983113	85984033	920	INS	AC2	EED	NM_001308007	intron10-intron10
1	231511056	231511088	32	DEL	AC2	EGLN1	NM_001377260	intron1-intron1
9	140563768	140564031	263	DUP	AC13	EHMT1	NM_001354611	intron1-intron1
2	212551251	212551549	298	DEL	AC9,AC13	ERBB4	NM_001042599	intron12-intron12
2	212879767	212879810	43	DEL	AC13	ERBB4	NM_001042599	intron2-intron2
2	212925311	212925645	334	INS	AC2	ERBB4	NM_001042599	intron2-intron2
2	213195781	213195883	102	INS	AC9	ERBB4	NM_001042599	intron1-intron1
8	27662510	27662840	330	DEL	AC2,AC13	ESCO2	NM_001017420	exon11-txEnd
15	76522854	76522942	88	INS	AC2,AC6,AC9	ETFA	NM_000126	intron10-intron10
12	12026415	12027153	738	DEL	AC2	ETV6	NM_001987	intron5-intron5
6	65152734	65152780	46	DEL	AC9,AC12	EYS	NM_001292009	intron26-intron26
6	65185158	65185472	314	DEL	AC2,AC9	EYS	NM_001292009	intron26-intron26
6	65342505	65342555	50	INS	AC13	EYS	NM_001292009	intron22-intron22
6	66163974	66164293	319	INS	AC2	EYS	NM_001292009	intron5-intron5
6	66260031	66262026	1995	DEL	AC2,AC6,AC12	EYS	NM_001292009	intron2-intron2
6	66274848	66275174	326	DEL	AC13	EYS	NM_001292009	intron2-intron2
6	66399046	66404687	5641	DEL	AC2	EYS	NM_001292009	intron1-intron1
13	113790943	113791271	328	DEL	AC2,AC6,AC9	F10	NM_000504	intron2-intron2
6	6277345	6277612	267	DEL	AC2,AC13	F13A1	NM_000129	intron3-intron3
1	197011863	197012161	298	INS	AC9	F13B	NM_001994	intron10-intron10
1	169524857	169525497	640	INS	AC9	F5	NM_000130	intron6-intron6
7	205219	205396	177	DUP	AC6	FAM20C	NM_020223	intron2-intron2
5	150914533	150914571	38	INS	AC9	FAT2	NM_001447	intron11-intron11
3	192063188	192063501	313	DEL	AC6,AC12,AC13	FGF12	NM_001377294	intron2-intron2
3	192358911	192359209	298	DEL	AC9,AC13	FGF12	NM_001377292	intron2-intron2
13	102813915	102814035	120	DEL	AC2	FGF14	NM_001321938	intron3-intron3

13	102886533	102886724	191	DEL	AC2	FGF14	NM_001321938	intron2-intron2
1	213039231	213039404	173	DEL	AC2,AC13	FLVCR1	NM_014053	intron2-intron2
1	240348222	240348519	297	DUP	AC2	FMN2	NM_001305424	intron4-intron4
3	71242353	71242653	300	DEL	AC2	FOXP1	NM_001244814	intron2-intron2
3	71376467	71376719	252	INS	AC13	FOXP1	NM_001244808	intron4-intron4
7	113953513	113953825	312	DEL	AC2	FOXP2	NR_033766	intron2-intron2
1	74755168	74755289	121	DEL	AC2,AC9	FPGT-TNNI3K/TNNI3K	NM_015978	intron5-intron5
4	79159508	79159856	348	INS	AC12	FRAS1	NM_025074	intron3-intron3
2	49354881	49354956	75	DEL	AC9	FSHR	NM_000145	intron1-intron1
9	71665331	71665566	235	DEL	AC2,AC6,AC9	FXN	NM_000144	intron2-intron2
9	101209337	101209650	313	DEL	AC2	GABBR2	NM_005458	intron7-intron7
9	101309046	101311668	2622	DEL	AC13	GABBR2	NM_005458	intron2-intron2
8	75375342	75375684	342	INS	AC13	GDAP1	NM_001362931	intron5-intron5
9	6629936	6629982	46	INS	AC2	GLDC	NM_000170	intron2-intron2
2	121680862	121680901	39	DEL	AC2,AC6	GLI2	NM_001371271	intron2-intron2
9	3953763	3953803	40	DEL	AC12	GLIS3	NM_001042413	intron4-intron4
18	11874564	11875042	478	INS	AC2	GNAL	NM_182978	intron10-intron10
12	102141412	102142068	656	INS	AC2	GNPTAB	NM_024312	intron20-intron20
12	65134835	65135012	177	DEL	AC9,AC13	GNS	NM_002076	intron6-intron6
13	95044890	95045027	137	DEL	AC13	GPC6	NM_005708	intron7-intron7
3	32153721	32153878	157	DEL	AC6	GPD1L	NM_015141	intron1-intron1
14	67012203	67012301	98	DEL	AC13	GPHN	NM_001024218	intron1-intron1
8	144295476	144295532	56	DEL	AC13	GPIHBP1	NM_001301772	intron1-intron1
1	240675801	240675984	183	INS	AC2,AC13	GREM2	NM_022469	intron1-intron1
1	240708772	240708808	36	DEL	AC6	GREM2	NM_022469	intron1-intron1
4	93381184	93381225	41	INS	AC13	GRID2	NM_001510	intron1-intron1
4	93393072	93393391	319	DEL	AC2	GRID2	NM_001510	intron1-intron1
4	93499194	93499254	60	DEL	AC6,AC9	GRID2	NM_001510	intron1-intron1
4	93567357	93570164	2807	DUP	AC12,AC13	GRID2	NM_001510	intron2-intron2
4	93579941	93580270	329	DEL	AC9	GRID2	NM_001510	intron2-intron2
4	94619025	94619065	40	INS	AC2	GRID2	NM_001510	intron14-intron14
4	94662315	94662348	33	DEL	AC2	GRID2	NM_001510	intron14-intron14
6	102172004	102172347	343	INS	AC13	GRIK2	NM_001166247	intron6-intron6
12	13941609	13941706	97	DEL	AC2	GRIN2B	NM_000834	intron3-intron3
15	28430091	28430340	249	INS	AC2	HERC2	NM_004667	intron56-intron56
15	72639547	72639915	368	INS	AC9	HEXA	NM_000520	intron10-intron10
7	81358335	81358612	277	DEL	AC2,AC6,AC13	HGF	NM_000601	intron8-intron8
2	191179019	191179054	35	DEL	AC9	HIBCH	NM_014362	intron1-intron1
6	143161426	143161510	84	DEL	AC2,AC9,AC13	HIVEP2	NM_006734	intron1-intron1
12	122289595	122289938	343	DEL	AC13	HPD	NM_002150	intron7-intron7
10	100496979	100497287	308	DEL	AC13	HPSE2	NM_021828	intron4-intron4
9	99062579	99062918	339	DEL	AC2	HSD17B3	NM_000197	intron1-intron1
3	129184473	129184785	312	DEL	AC6,AC12	IFT122	NM_001280545	intron8-intron8
6	160521749	160521819	70	DEL	AC2,AC9	IGF2R	NM_000876	intron45-intron45
8	42185321	42185632	311	DEL	AC9	IKBKB	NM_001242778	intron19-intron19
10	6097375	6097877	502	DEL	AC2,AC6,AC9	IL2RA	NM_000417	intron1-intron1
14	105186822	105186923	101	DEL	AC13	INF2	NM_001031714	exon22-exon22
15	40714963	40715150	187	INS	AC6	IVD	NM_001354598	intron11-intron11
16	87705207	87705258	51	DEL	AC2	JPH3	NR_073379	intron2-intron2

11	128785941	128786104	163	DEL	AC2	KCNJ5	NM_000890	intron2-intron2
21	39050079	39050152	73	INS	AC13	KCNJ6	NM_002240	intron3-intron3
21	39080016	39080521	505	INS	AC13	KCNJ6	NM_002240	intron3-intron3
21	39196237	39196334	97	INS	AC2,AC6,AC9,AC12	KCNJ6	NM_002240	intron2-intron2
21	39233185	39233234	49	DEL	AC2	KCNJ6	NM_002240	intron1-intron1
10	78800905	78801227	322	DEL	AC2,AC13	KCNMA1	NM_001322838	intron15-intron15
20	62081875	62081939	64	DUP	AC6	KCNQ2	NM_004518	intron1-intron1
16	27724967	27725198	231	INS	AC2	KIAA0556/LOC100128079	NM_015202	intron13-intron13
4	123188205	123188257	52	INS	AC2	KIAA1109	NM_015312	intron44-intron44
2	10184250	10184297	47	DEL	AC2,AC9,AC13	KLF11	NM_003597	intron1-intron1
5	137022565	137023897	1332	DEL	AC2,AC9,AC12	KLHL3	NM_001257195	intron3-intron3
12	53207592	53207637	45	INS	AC6	KRT4	NM_002272	exon1-exon1
18	21490977	21491121	144	DEL	AC6	LAMA3	NM_001127717	intron54-intron54
1	66055935	66056272	337	INS	AC12	LEPR	NM_001003680	intron5-intron5
1	66079149	66079422	273	DEL	AC6,AC13	LEPR	NM_001003680	intron14-intron14
1	66079459	66079518	59	INS	AC2,AC12	LEPR	NM_001003680	intron14-intron14
10	95545474	95546468	994	DEL	AC2,AC13	LGI1	NM_001308275	intron4-intron4
15	77910867	77911242	375	DEL	AC2	LINGO1	NM_001301199	intron3-intron3
15	77991616	77992116	500	DUP	AC9	LINGO1	NM_001301198	intron1-intron1
16	964490	964824	334	DUP	AC2	LMF1	NM_001352021	intron3-intron3
18	2997373	2997457	84	DEL	AC13	LPIN2	NM_014646	intron1-intron1
3	188304835	188305178	343	DEL	AC2,AC6,AC9	LPP	NM_001375455	intron5-intron5
3	188473595	188473635	40	DEL	AC9,AC13	LPP	NM_001375455	intron7-intron7
4	151875131	151875221	90	INS	AC6,AC13	LRBA	NM_001364905	intron2-intron2
10	78095877	78095923	46	INS	AC6	LRMDA	NM_001305581	intron6-intron6
11	68183094	68183412	318	DEL	AC2,AC6,AC13	LRP5	NM_001291902	intron12-intron12
9	130226648	130226703	55	DEL	AC2	LRSAM1	NM_001005373	intron9-intron9
22	21343986	21344023	37	DEL	AC13	LZTR1	NM_006767	intron7-intron7
7	1927503	1927634	131	INS	AC9	MAD1L1	NM_001304525	intron3-intron3
7	2042682	2042744	62	INS	AC2,AC6	MAD1L1	NM_001013836	intron13-intron13
7	2200745	2200840	95	INS	AC6	MAD1L1	NM_001013836	intron10-intron10
11	47321173	47321229	56	INS	AC2	MADD	NM_001376651	intron22-intron22
7	78357850	78358190	340	DEL	AC9	MAGI2	NM_001301128	intron2-intron2
7	78606783	78606824	41	DEL	AC6	MAGI2	NM_001301128	intron2-intron2
11	96001924	96003394	1470	DEL	AC2	MAML2	NM_032427	intron1-intron1
6	91237581	91237902	321	DEL	AC9	MAP3K7	NM_003188	intron12-intron12
18	32661965	32662283	318	DEL	AC6	MAPRE2	NM_001143826	intron3-intron3
6	119164813	119164861	48	INS	AC12,AC13	MCM9	NM_017696	intron7-intron7
12	116715140	116715261	121	INS	AC6	MED13L	NM_015335	exon1-exon1
5	88032019	88032354	335	DEL	AC2,AC6,AC9,AC13	MEF2C	NM_001364353	intron4-intron4
5	88043148	88043474	326	DEL	AC2	MEF2C	NM_001364353	intron4-intron4
22	42140481	42140804	323	DEL	AC6,AC12	MEI1	NM_152513	intron12-intron12
3	70013474	70013887	413	DEL	AC12	MITF	NM_001354604	intron9-intron9
21	33672992	33673079	87	INS	AC2,AC6	MRAP	NM_001285394	intron2-intron2
5	80039370	80039681	311	DEL	AC9	MSH3	NM_002439	intron11-intron11
8	15997256	15997314	58	DEL	AC9,AC13	MSR1	NM_138716	intron8-intron8
9	113463930	113464147	217	DEL	AC12	MUSK	NM_001166280	intron5-intron5
12	102030825	102030909	84	INS	AC2,AC9,AC13	MYBPC1	NM_001254718	intron8-intron8
16	15869266	15869316	50	DEL	AC9	MYH11	NM_001040113	intron9-intron9

18	47694973	47698387	3414	DEL	AC12	MYO5B	NM_001080467	intron1-intron1
2	1865365	1865769	404	DUP	AC2,AC9,AC13	MYT1L	NM_001329845	intron18-intron18
2	2239127	2239195	68	DEL	AC13	MYT1L	NM_001329845	intron2-intron2
2	2266692	2266749	57	INS	AC2	MYT1L	NM_001329845	intron2-intron2
4	140289133	140289177	44	DEL	AC6	NAA15	NM_057175	intron14-intron14
13	101894143	101896424	2281	DEL	AC12	NALCN	NM_001350748	intron11-intron11
2	240958945	240959270	325	DEL	AC2,AC9	NDUFA10	NR_136158	intron3-intron3
18	55801583	55801911	328	DEL	AC2,AC6,AC9	NEDD4L	NM_001144967	intron1-intron1
17	29662295	29662409	114	INS	AC2,AC9	NF1	NM_000267	intron39-intron39
2	69650285	69650336	51	DEL	AC12	NFU1	NM_015700	intron3-intron3
3	25802351	25802392	41	DEL	AC2,AC9,AC13	NGLY1	NM_018297	intron3-intron3
3	25824977	25825022	45	DEL	AC9	NGLY1	NM_001145294	intron1-intron1
X	17502751	17502820	69	DEL	AC6	NHS	NM_001291867	intron1-intron1
17	5464742	5465066	324	DEL	AC6	NLRP1	NM_001033053	intron3-intron3
9	139422831	139423032	201	DUP	AC2,AC13	NOTCH1	NM_017617	intron2-intron2
19	15284535	15284571	36	DEL	AC2	NOTCH3	NM_000435	intron25-intron25
2	50602635	50602974	339	INS	AC13	NRXN1	NM_001330078	intron17-intron17
8	126336808	126337124	316	DEL	AC2	NSMCE2	NM_001349485	intron4-intron4
1	156806529	156806581	52	DEL	AC2	NTRK1	NM_001007792	intron1-intron1
9	87345362	87345678	316	DEL	AC13	NTRK2	NM_001007097	intron11-intron11
17	801621	802218	597	DEL	AC2	NXN	NM_022463	intron1-intron1
15	28175796	28176205	409	INS	AC2	OCA2	NM_000275	intron18-intron18
11	132845424	132845456	32	DEL	AC2	OPCML	NM_001012393	intron1-intron1
8	107417374	107417420	46	DEL	AC9	OXR1	NM_001198533	intron2-intron2
8	107511104	107511428	324	DEL	AC2	OXR1	NM_001198533	intron2-intron2
8	107728565	107728894	329	DEL	AC13	OXR1	NM_001198533	intron11-intron11
11	93871030	93871132	102	INS	AC6	PANX1	NM_015368	intron1-intron1
1	19070924	19071004	80	INS	AC13	PAX7	NM_001135254	intron8-intron8
1	164845210	164845255	45	DEL	AC12	PBX1	NM_001353131	intron7-intron7
3	136021010	136026196	5186	DEL	AC2	PCCB	NM_001178014	intron10-intron10
10	55893677	55894125	448	DEL	AC6	PCDH15	NM_001142771	intron15-intron15
10	56762357	56762424	67	INS	AC2	PCDH15	NM_001354404	intron3-intron3
5	58327196	58327231	35	DEL	AC6	PDE4D	NM_001197221	intron2-intron2
10	95414513	95414548	35	DEL	AC9	PDE6C	NM_006204	intron15-intron15
17	79619832	79619886	54	DEL	AC6,AC13	PDE6G	NM_001365725	intron2-intron2
12	7355778	7356081	303	INS	AC13	PEX5	NM_001351124	intron8-exon9
1	64112928	64112963	35	DEL	AC9	PGM1	NM_002633	intron7-intron7
11	46036244	46036590	346	DEL	AC2	PHF21A	NM_001352028	intron6-intron6
18	10757445	10757525	80	DEL	AC2,AC12	PIEZO2	NM_022068	intron25-intron25
18	10915932	10915973	41	DEL	AC6	PIEZO2	NM_022068	intron3-intron3
18	59728660	59728768	108	DEL	AC2	PIGN	NM_012327	intron29-intron29
18	59834525	59834560	35	DEL	AC13	PIGN	NM_012327	intron1-intron1
11	17195845	17196157	312	DEL	AC13	PIK3C2A	NM_001321378	intron2-intron2
17	6422505	6422670	165	DEL	AC9	PITPNM3	NM_001165966	intron2-intron2
6	51517356	51517682	326	DEL	AC2,AC13	PKHD1	NM_138694	intron61-intron61
6	51739569	51745614	6045	DEL	AC9,AC13	PKHD1	NM_138694	intron46-intron46
12	32963605	32963921	316	DEL	AC2,AC6,AC13	PKP2	NM_004572	intron10-intron10
20	9389098	9389164	66	INS	AC9	PLCB4	NM_001377134	intron21-intron21
12	80239678	80239721	43	INS	AC9	PPP1R12A	NM_001143886	intron2-intron2

5	146026781	146026825	44	INS	AC2,AC6,AC13	PPP2R2B	NR_073526	intron5-intron5
5	146394710	146397305	2595	DEL	AC9	PPP2R2B	NM_001271900	intron2-intron2
5	146396114	146396176	62	INS	AC6	PPP2R2B	NM_001271900	intron2-intron2
5	146396115	146396177	62	INS	AC6	PPP2R2B	NM_001271900	intron2-intron2
4	121808935	121809250	315	DEL	AC9	PRDM5	NM_001300823	intron2-intron2
5	122431837	122432027	190	INS	AC9	PRDM6	NM_001136239	intron2-intron2
12	42871291	42871330	39	DEL	AC2,AC9	PRICKLE1	NM_001144881	intron1-intron1
10	53947968	53948034	66	DEL	AC13	PRKG1	NM_001098512	intron9-intron9
6	161795626	161795801	175	INS	AC2,AC13	PRKN	NM_004562	intron10-intron10
6	161973423	161973482	59	DEL	AC2,AC9	PRKN	NM_004562	intron8-intron8
1	214576009	214576061	52	INS	AC9,AC13	PTPN14	NM_005401	intron6-intron6
11	48120066	48120374	308	DEL	AC9	PTPRJ	NM_001098503	intron1-intron1
2	1723678	1724041	363	DEL	AC9	PXDN	NM_012293	intron1-intron1
14	51408899	51409530	631	DEL	AC13	PYGL	NM_001163940	intron1-intron1
11	64526480	64526561	81	INS	AC2,AC6	PYGM	NM_001164716	intron1-intron1
3	25030886	25032242	1356	DEL	AC2,AC13	RARB	NM_001290216	intron2-intron2
3	25508991	25509031	40	DEL	AC9	RARB	NM_001290216	intron5-intron5
8	53611229	53611262	33	DEL	AC2,AC9	RB1CC1	NM_014781	intron1-intron1
7	103463073	103463395	322	DEL	AC2,AC9	RELN	NM_173054	intron3-intron3
1	8729019	8729097	78	DEL	AC6,AC9	RERE	NM_001042681	intron1-intron1
6	151771280	151771319	39	DEL	AC2	RMND1	NM_017909	intron1-intron1
3	149593684	149593794	110	INS	AC9,AC13	RNF13	NM_007282	intron5-intron5
4	1086772	1086826	54	DEL	AC2,AC9	RNF212	NM_001366918	intron3-intron3
14	21778182	21778401	219	DEL	AC2,AC13	RPGRIP1	NM_020366	intron6-intron6
16	53713453	53713556	103	DEL	AC13	RPGRIP1L	NM_001127897	intron6-intron6
2	89001136	89001221	85	INS	AC2,AC9,AC13	RPIA	NM_144563	intron3-intron3
11	14348317	14348634	317	DEL	AC6	RRAS2	NM_001177315	intron1-intron1
1	38077349	38077420	71	DEL	AC2,AC6,AC9	RSPO1	NM_001038633	intron8-intron8
16	57231835	57231879	44	DEL	AC2	RSPRY1	NM_001305182	intron1-intron1
16	57252627	57252950	323	DEL	AC2	RSPRY1	NM_001305163	intron8-intron8
1	237449856	237450067	211	INS	AC9,AC13	RYR2	NM_001035	intron2-intron2
1	237679029	237679180	151	INS	AC6	RYR2	NM_001035	intron24-intron24
8	119165528	119165600	72	DEL	AC2	SAMD12	NR_146234	intron4-intron4
8	119447054	119447364	310	DEL	AC6	SAMD12	NR_146234	intron3-intron3
8	119618005	119618050	45	DEL	AC2,AC6	SAMD12	NR_146234	intron1-intron1
3	39011493	39011530	37	DEL	AC12	SCN11A	NM_001349253	intron2-intron2
6	108266118	108266421	303	DEL	AC9	SEC63	NM_007214	intron1-intron1
6	158548271	158549075	804	DEL	AC2,AC6,AC9,AC13	SERAC1	NM_032861	intron10-intron10
17	1654662	1655386	724	DEL	AC13	SERPINF2	NM_000934	intron8-intron8
5	155777256	155777300	44	INS	AC2,AC6	SGCD	NM_000337	intron3-intron3
5	148401441	148401598	157	DEL	AC9	SH3TC2	NM_024577	intron12-intron12
15	48550270	48550592	322	DEL	AC13	SLC12A1	NM_000338	intron16-intron16
5	36649430	36649465	35	DEL	AC13	SLC1A3	NM_001289939	intron2-intron2
14	92941046	92941091	45	DEL	AC2,AC6	SLC24A4	NM_153648	intron12-intron12
3	45831052	45831107	55	DEL	AC6,AC13	SLC6A20	NM_020208	intron1-intron1
5	492290	492351	61	DEL	AC13	SLC9A3	NM_001284351	intron1-intron1
18	2748477	2748513	36	DEL	AC2,AC9	SMCHD1	NM_015295	intron30-intron30
15	25621911	25621955	44	INS	AC2	SNHG14/UBE3A	NM_001354506	intron6-intron6
2	39306291	39306358	67	INS	AC13	SOS1	NM_005633	intron1-intron1

12	24460447	24460731	284	DEL	AC13	SOX5	NM_001261414	intron3-intron3
14	88890014	88890172	158	DEL	AC6,AC13	SPATA7	NM_001040428	intron4-intron4
22	24712801	24713504	703	DEL	AC9	SPECC1L/SPECC1L-ADORA2A	NM_001145468	intron3-intron3
5	35752763	35752928	165	INS	AC2	SPEF2	NM_024867	intron23-intron23
2	220321615	220321683	68	DEL	AC2,AC6,AC13	SPEG	NM_005876	intron6-intron6
5	147462158	147462476	318	DEL	AC9,AC12,AC13	SPINK5	NM_001127699	intron4-intron4
14	78036158	78036303	145	INS	AC6,AC13	SPTLC2	NM_004863	intron5-intron5
14	35476847	35476880	33	DEL	AC9	SRP54	NM_001146282	intron4-intron4
3	133503776	133505093	1317	DEL	AC9	SRPRB/TF	NM_001354703	exon23-exon23
2	96862806	96862859	53	INS	AC2	STARD7	NM_020151	intron1-intron1
19	7708892	7708961	69	INS	AC13	STXBP2	NM_001127396	intron13-intron13
17	80856030	80856094	64	DEL	AC6	TBCD	NM_005993	intron17-intron17
4	107056577	107063362	6785	DEL	AC13	TBCK	NM_001163435	intron23-intron23
11	12943735	12944014	279	DEL	AC2	TEAD1	NM_021961	intron10-intron10
11	78516321	78516389	68	INS	AC2	TENM4	NM_001098816	exon15-intron15
3	24306049	24306093	44	DEL	AC9,AC13	THRB	NM_000461	intron2-intron2
9	71738120	71743354	5234	DEL	AC2	TJP2	NM_001170414	intron1-intron1
9	71777933	71778252	319	DEL	AC9,AC12	TJP2	NM_001170414	intron2-intron2
11	61107790	61108130	340	DEL	AC6	TKFC	NM_001351978	intron5-intron5
3	53269956	53270027	71	INS	AC2	TKT	NM_001135055	intron4-intron4
4	166987132	166987267	135	INS	AC9	TLL1	NM_012464	intron16-intron16
4	167004312	167004908	596	DEL	AC2	TLL1	NM_012464	intron18-intron18
1	165731218	165731496	278	DEL	AC13	TMCO1	NM_019026	intron2-intron2
7	12281703	12282030	327	DEL	AC6,AC12,AC13	TMEM106B	NM_001134232	exon8-exon8
3	189363420	189370899	7479	DEL	AC2	TP63	NM_001329964	intron1-intron1
3	189575904	189576229	325	DEL	AC2,AC9	TP63	NM_001329964	intron4-intron4
7	144382754	144382903	149	DEL	AC2,AC9,AC12,AC13	TPK1	NM_001350884	intron1-intron1
8	140884957	140885165	208	DEL	AC2,AC6,AC13	TRAPPC9	NM_001160372	intron21-intron21
8	141361276	141361615	339	INS	AC2	TRAPPC9	NM_001160372	intron9-intron9
6	123924305	123924403	98	INS	AC2	TRDN	NM_006073	intron1-intron1
4	154095270	154095370	100	INS	AC13	TRIM2	NM_001375517	intron1-intron1
5	14347118	14347525	407	DUP	AC9	TRIO	NM_007118	intron11-intron11
8	116488226	116488556	330	DEL	AC2,AC6,AC13	TRPS1	NM_001282902	intron4-intron4
7	98550659	98550695	36	DEL	AC2,AC12,AC13	TRRAP	NM_001244580	intron38-intron38
14	81469121	81469435	314	DEL	AC6,AC9,AC13	TSHR	NM_001018036	intron1-intron1
14	81597047	81597366	319	DEL	AC13	TSHR	NM_000369	intron8-intron8
2	166787240	166787335	95	INS	AC13	TTC21B	NM_024753	intron8-intron8
4	147730176	147731088	912	DEL	AC13	TTC29	NM_001300761	intron11-intron11
2	179621885	179621984	99	INS	AC13	TTN	NM_001256850	intron44-intron44
2	210821023	210821250	227	INS	AC6,AC9	UNC80	NM_001371986	intron48-intron48
12	109544696	109544852	156	INS	AC2	UNG	NM_080911	intron6-intron6
11	17548693	17548744	51	DEL	AC6,AC9	USH1C	NM_001297764	intron5-intron5
1	216085794	216085857	63	DEL	AC9,AC12	USH2A	NM_206933	intron38-intron38
1	216185310	216185482	172	DEL	AC2,AC12	USH2A	NM_206933	intron32-intron32
X	41057618	41057683	65	DEL	AC6	USP9X	NM_001039590	intron29-intron29
4	1366458	1366690	232	DUP	AC2	UVSSA	NM_001317934	intron8-intron8
12	6574523	6574574	51	DEL	AC6	VAMP1	NM_001297438	intron3-intron3
2	20185026	20185288	262	DUP	AC13	WDR35	NM_001006657	intron2-intron2
16	78178327	78178402	75	INS	AC2,AC9	WWOX	NM_130791	intron4-intron4

16	78227441	78227868	427	INS	AC13	WWOX	NM_130791	intron5-intron5
3	14196291	14196373	82	DEL	AC2	XPC	NM_001354727	intron9-intron9
1	180828198	180828253	55	INS	AC2,AC6,AC13	XPR1	NM_001135669	intron10-intron10
5	82482756	82483062	306	DEL	AC2	XRCC4	NM_022550	intron3-intron3
3	114466479	114466804	325	DEL	AC2,AC9	ZBTB20	NM_001164343	intron4-intron4
2	145255390	145255441	51	DEL	AC9	ZEB2	NM_001171653	intron2-intron2
8	106670527	106670846	319	DEL	AC13	ZFPM2	NM_001362836	intron4-intron4
10	80925712	80925773	61	INS	AC2,AC13	ZMIZ1	NM_020338	intron4-intron4
16	88478396	88478501	105	INS	AC2,AC6,AC9	ZNF469	NM_001367624	intron1-intron1

Supplementary Table H: 7 class-4 SVs and 7 genes found in 5 unaffected relatives but not in 10 probands

Chromosome	Start	End	Length	Type	Sample	Gene	Transcript	Location
4	3467915	3474537	6622	DUP	AC9	DOK7	NM_001164673	intron2-intron2
19	51860637	51860981	344	INS	AC2	ETFB	NM_001985	intron1-intron1
10	78066263	78066336	73	DEL	AC6	LRMDA	NM_001305581	intron5-intron5
7	77863868	77863950	82	DEL	AC2	MAGI2	NM_001301128	intron10-intron10
7	47895181	47895508	327	DEL	AC2	PKD1L1	NM_138295	intron28-intron28
10	53461462	53461501	39	DEL	AC13	PRKG1	NM_001098512	intron3-intron3
17	80732491	80732528	37	DEL	AC9	TBCD	NM_005993	intron6-intron6
